# Novel *N*-chloroacetyl-2-pyrazoline analogs with 2-naphthyl and ferrocenyl groups: targeting histamine receptor H1 to overcome colorectal cancer drug resistance

**DOI:** 10.20517/cdr.2025.203

**Published:** 2026-04-30

**Authors:** Dileepkumar Veeragoni, Hindole Ghosh, Affan Ansari, Sangita Bhattacharyya, Ahraar Azaz, Ojaswitha Ommi, Obdulia Covarrubias-Zambrano, Leonhard H. F. Köhler, Justus F. Ködel, Ameer Hamza, Anup Kasi, Stefan H. Bossmann, Rainer Schobert, Bernhard Biersack, Prasad Dandawate

**Affiliations:** ^1^Cancer Biology, University of Kansas Medical Center, Kansas City, KS 66160, USA.; ^2^Organische Chemie I, Universität Bayreuth, Bayreuth 95447, Germany.; ^3^Pathology and Laboratory Medicine, University of Kansas Medical Center, Kansas City, KS 66160, USA.; ^4^Medical Oncology, University of Kansas Medical Center, Kansas City, KS 66160, USA.; ^#^Authors contributed equally.

**Keywords:** 2-Pyrazoline, multi-component reaction, drug resistance, apoptosis, xenograft model, colon cancer, HRH1, histamine

## Abstract

**Aim:** This study investigates the anticancer activity of naphthalene- and ferrocene-based 2-pyrazolines against colorectal cancer (CRC) cell lines and assesses their potential to overcome chemotherapy resistance.

**Methods:** Several 2-pyrazoline derivatives were synthesized and tested for anticancer activity across various cell lines, including p53 wild-type (WT) and knockout (KO) colon cancer cells and vinblastine-resistant KB-V1 cervix carcinoma cells. Compounds **Clac10** (**5c**) and **Clac12** (**5e**) were studied for their effects on colony and spheroid formation, cell cycle, and apoptosis. Molecular docking and cellular thermal shift assays explored their binding to histamine receptor H1 (HRH1). *In vivo* antitumor efficacy was tested on HCT116 xenografts in NSG mice.

**Results:** Compounds **Clac10** and **Clac12** significantly inhibited the proliferation of both p53 WT and KO colon cancer cells, as well as drug-resistant KB-V1 cells. When combined with 5-fluorouracil (5-FU), they showed synergistic antiproliferative effects in HCT and DLD1 cells. These compounds reduced colony and spheroid formation, induced cell cycle arrest, and promoted apoptosis by downregulating cyclin D1 and antiapoptotic proteins [B-cell lymphoma-extra large (Bcl-XL), B-cell lymphoma 2 (Bcl-2), myeloid cell leukemia 1 (Mcl-1)]. Molecular docking and thermal shift assays confirmed binding to HRH1, affecting histamine-induced extracellular signal-regulated kinase (ERK) and glycogen synthase kinase 3 beta (GSK3B) signaling. *In vivo*, **Clac10** significantly reduced HCT116 xenograft growth, decreased Ki-67 and phosphorylated-glycogen synthase kinase 3 beta (p-GSK3B) levels, and increased cleaved caspase-3.

**Conclusion:** Naphthalene-based 2-pyrazoline compounds **Clac10** and **Clac12** showed a potent anticancer activity against colon cancer lines. They inhibit tumor growth by targeting HRH1 signaling, indicating potential as CRC therapies and resistance-overcoming agents. Further studies are needed to explore their clinical potential.

## INTRODUCTION

Colorectal cancer (CRC) remains one of the leading causes of cancer-related morbidity and mortality worldwide^[1]^. While advances in surgical techniques and chemotherapy have been made, the development of resistance to primary drugs such as 5-fluorouracil (5-FU), oxaliplatin, and irinotecan often compromises treatment efficacy^[2]^. This resistance is a significant challenge in CRC treatment, often resulting in disease progression and poor patient outcomes^[2]^. Moreover, genetic alterations, including the loss of the tumor suppressor gene p53, further exacerbate the problem by diminishing the effectiveness of standard therapies^[3]^. Chemoresistance in CRC is also driven by tumor-promoting oncogenic mutations [e.g., Rat sarcoma (Ras), B-Raf proto-oncogene, serine/threonine kinase (BRAF)], anti-apoptotic mechanisms, and sophisticated drug-detoxifying systems (such as DNA repair pathways, drug transporters, and cytochromes)^[4]^. Additionally, colon cancer stem cells (CSCs) have the capacity to resist and survive chemotherapy, leading to disease relapse with drug-resistant tumors^[5]^. Therefore, there is an urgent need for the development of new and more effective therapies to treat CRC, particularly for patients with relapsed or refractory disease, to achieve significant improvements in survival rates and patient outcomes.

Histamine receptor H1 (HRH1) promotes CRC cell proliferation, while HRH2 inhibits tumor growth in inflammation-related CRC in mice^[6]^. Activating HRH2 could offer anticancer benefits but may increase gastric acid secretion, raising the risk of reflux and ulcers. Therefore, targeting HRH1 is a safer strategy. Overexpression of HRH1 has been linked to tumor progression and poor prognosis in hepatocellular carcinoma (HCC)^[7]^. Recent studies also suggest that HRH1 may contribute to resistance against anti-PD1 immunotherapy in cancers such as melanoma^[8]^ and pancreatic cancer^[9]^. Blocking HRH1 could improve the effectiveness of this therapy. Therefore, HRH1 might be a potential target for CRC treatment. Some antihistamines can control tumor growth, but long-term use of first-generation drugs causes side effects, such as neurological effects, including drowsiness. Second-generation drugs are better for long-term use, but dose-response studies for cancer treatment are not well established^[10]^. Hence, there is a need to identify novel compounds that selectively target HRH1 for CRC treatment.

**Scheme 1:**
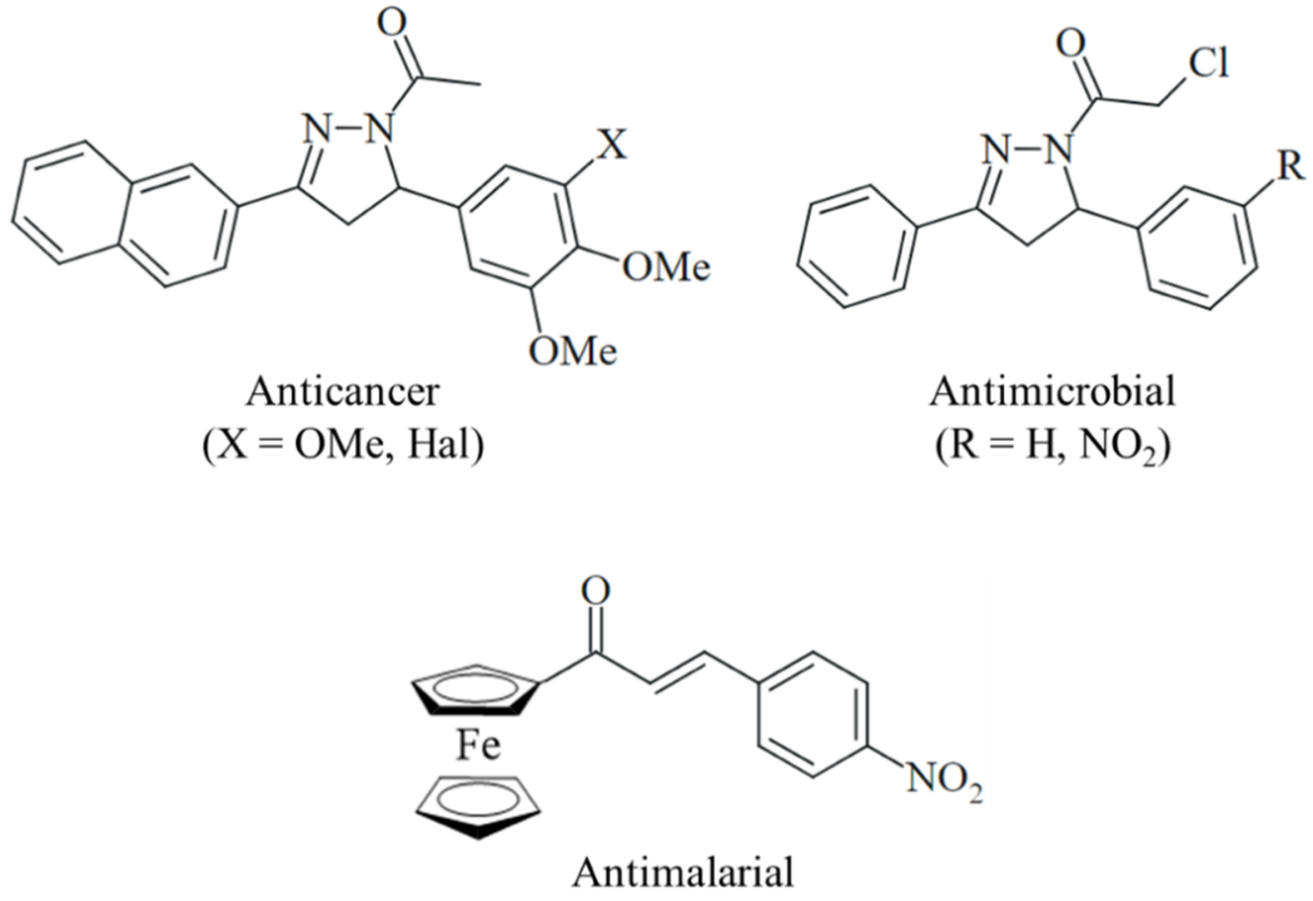
Examples of biologically active *N*-acyl 2-pyrazolines (Hal = Cl, Br, or I) and an antimalarial ferrocene-based chalcone.

Nitrogen heterocycles (*N*-heterocycles) are a prominent and growing class of U.S. Food and Drug Administration (FDA)-approved chemotherapeutic drugs^[11]^. In particular, 5-membered *N*-heterocycles, such as pyrazoles and pyrazolines, have been repeatedly described as potent anticancer drug candidates^[12,13]^. 3,5-Diaryl-substituted 2-pyrazolines are especially attractive from a chemical point of view because they can be easily prepared using one-pot procedures from chalcone-based starting compounds^[14,15]^. *N*-acyl substituted pyrazolines are available from three-component reactions using aqueous hydrazine and acyl reagents as solvents [Scheme 1]^[16-18]^. Various chloroacetyl-2-pyrazolines exhibited considerable antimicrobial activities [Scheme 1]^[19]^. The chloroacetamide-functionalized fumagillin derivative TNP-470 was identified early as a potent angiogenesis inhibitor^[20]^. In addition, alkylating chloroacetamide moieties were investigated as anticancer drug candidates, some of them with proven plasma stability and amenable bioavailability, that interact with crucial cysteines and selenocysteines of protein kinases [epidermal growth factor receptor (EGFR), fibroblast growth factor receptor (FGFR)], glutathione-modifying enzymes [glutathione S-transferase omega 1 (GSTO1), glutathione peroxidase 4 (GPX4)], and methyltransferases [nicotinamide N-methyltransferase (NNMT), protein arginine methyltransferase 1 (PRMT1)]^[21-28]^. Moreover, anticancer-active curcuminoids modified with alkylating chloroacetamide moieties were described^[29]^. Promising anticancer-active chloroacetamides, which target DNA and G-quadruplexes in bladder cancers^[30]^, were also disclosed.

Naphthalene-based ring systems are important scaffolds in various drugs and drug candidates, including anticancer agents^[31]^. Naphthyl-substituted *N*-acetyl-2-pyrazolines showed tubulin-interacting and ATP-binding cassette (ABC)-transporter inhibitory activities [Scheme 1]^[32,33]^. Isoxazole- and pyrazole-bridged naphthyl-combretastatins with pronounced anticancer activities were described, and the replacement of the 3-hydroxy-4-methoxyphenyl ring of the natural tubulin polymerization inhibitor combretastatin A-4 (C-A4) by a 2-naphthyl group conserved the high activity of C-A4^[34]^. Ferrocene is also an interesting biologically active scaffold used for the synthesis of antiparasitic chalcones [Scheme 1] and as a benzene/arene isostere in anticancer-active tamoxifen analogs (ferrocifens)^[35,36]^. The promising anticancer activities of ferrocenes have been described for conjugates with hydroxamic acids, indoles, naphthoquinones, and a kinase inhibitor^[37-39]^. Both naphthalene and ferrocene can undergo oxidation processes that contribute to their biological properties, including their interactions with vital biomolecules and the formation of toxic reactive oxygen species (ROS)^[31,33]^. Although 2-naphthyl- and ferrocenyl-based *N*-chloroacetyl-substituted 2-pyrazolines have been investigated for their anticancer properties, they haven’t been extensively examined in cancer mouse models, and their potential to target histamine-HRH1 axes remains unexplored. This represents a novel target to counteract the upregulation of anti-apoptotic proteins and overcome drug resistance in colon cancer. This work details the synthesis and anticancer testing of new naphthyl- and ferrocenyl-substituted 2-pyrazolines with *N*-chloroacetyl groups at the pyrazoline ring. It focuses on their effects *in vitro* and *in vivo* in CRC models, their impact on histamine-HRH1 signaling and tumor growth, and their potential to inhibit drug resistance.

## METHODS

### Chemistry

Starting compounds and reagents were purchased from abcr (Karlsruhe, Germany), Aldrich/Merck (Darmstadt, Germany), Alfa Aesar (Kandel, Germany), and TCI (Zwijndrecht, Belgium). Chalcones **2a-c**, **2h**, and **3a-g** were prepared according to literature procedures^[32,33,37,40]^. The synthesis of the new ferrocene-based chalcones **2d-g** is described in the Supplementary Materials. Compound **5a** was described as a reagent for the synthesis of an isatin conjugate without disclosing synthetic details or analytical data^[41]^. Column chromatography used silica gel 60 (230-400 mesh). Melting points (uncorrected) measured by Electrothermal 9100; IR [attenuated total reflectance (ATR)] spectra recorded with Perkin-Elmer Spectrum One Fourier-transform infrared (FT-IR) spectrophotometer; nuclear magnetic resonance (NMR) spectra obtained with Bruker Avance 300 spectrometer; chemical shifts given in ppm (δ) downfield from internal standard tetramethylsilane (TMS); Mass spectra acquired with Thermo Finnigan MAT 8500 [electron ionization (EI)] and UPLC/Orbitrap [electrospray ionization - high-resolution mass spectrometry (ESI-HRMS)]; Elemental analyses performed using Perkin-Elmer 2400 CHN elemental analyzer [Supplementary Materials].

### Hydrolytic stability study

The hydrolytic stability study was used to assess the stability of compounds to hydrolysis^[42]^. Stock solutions of compounds **5c** and **5e** were prepared in dimethyl sulfoxide (DMSO) at a concentration of 20 mM. Subsequently, 2 μL of a stock solution of compounds **5c** and **5e**, or DMSO as a vehicle control, was added to 2 mL of phosphate-buffered saline (PBS). Absorption spectra were recorded by sampling aliquots at specified intervals over a 24-hour period.

### Cell line and culture conditions

HCT116 and DLD1 colon cancer, THP-1, Jurkat and cell lysates of FAP (cells from Familial Adenomatous Polyposis tissues and its adjacent uninvolved tissue (non-FAP) and CRC cell lines were gift from Anant lab, Het1a epithelial cells (Sigma), HCT116 p53-knockout (KO) colon carcinoma, 518A2 melanoma (Department of Radiotherapy, Medical University of Vienna, Austria), MCF7 (ACC-115) breast carcinoma, HeLa and vinblastine-resistant KB-V1(Vbl) cervix carcinoma (ACC-149), U87 glioblastoma, and EaHy.926 (ATCC® CRL-2922™) endothelial hybrid cells were grown in complete Dulbecco’s Modified Eagle Medium (DMEM) with 10% fetal bovine serum and 1% antibiotic-antimycotic solution at 37 °C, 5% CO_2_, and 95% humidity. Vinblastine-resistant KB-V1(Vbl) cells were treated with 340 nM vinblastine to maintain resistance and P-glycoprotein (P-gp) expression.

### Proliferation assays

Cancer cells (5 × 10^3^ cells/well, 100 μL/well) were seeded in 96-well plates for 24 h. After treatment with 0-100 μM of test compounds **4a-e** and **5b-f** for 72 h, 12.5 μL of 0.5% MTT in PBS was added and incubated 2-4 h at 37 °C. DMSO was the negative control. Plates were centrifuged (300 g, 5 min, 4 °C), medium discarded, and formazan dissolved in 25 μL DMSO with 10% sodium dodecyl sulfate (SDS) and 0.6% acetic acid for 1 h in the dark at 37 °C. Absorbance at 570 nm (formazan) and 630 nm (background) measured with Tecan Infinite F200^[43,44]^. The background absorbance was subtracted from the formazan absorbance readings. A cell counting kit-8 (CCK-8) assay (Abcam, Cat no. ab228554, Burlingame, CA, USA) was used to assess cell viability in THP-1 and Jurkat cells. THP-1 and Jurkat cells were seeded at 5,000 cells/well in 96-well plates. After 24 h, cells were treated with compounds **5c** and **5e**. Then, 10 μL of CCK-8 reagent was added to the well, and the plates were incubated for 1 h at 37 °C. Absorbance at 450 nm was measured to assess viability^[45]^. An enzymatic hexosaminidase assay^[46,47]^ was used to assess Het1a cell viability at different time points. The percent change in proliferation was calculated by comparing treated cell viability to untreated controls (< 1% DMSO). Half maximal inhibitory concentration (IC_50_) values were derived from dose-inhibition curves, with vehicle controls set to 100%. The “log(inhibitor) *vs.* normalized response-Variable slope” curve fitting was used (GraphPad Prism 9).

### Proliferation assay of 5-FU combined with compounds 5c and 5e

Cancer cells HCT116 and DLD1 (5 × 10^3^ cells/well, 100 μL/well) were seeded in 96-well plates. After 24 h, the cells were treated with compound **5c** or **5e** and with a combination of 5-FU at increasing concentrations from 0 to 5 μM. After 72 h of treatment, an enzymatic hexosaminidase assay^[46,47]^ was used to assess cell viability. The percent change in proliferation was calculated by comparing treated cell viability to untreated controls (< 1% DMSO). The expected drug-combination responses were calculated using the ZIP model in SynergyFinder^[48]^. Deviations between observed and expected responses with positive and negative values denote synergy and antagonism, respectively. For estimating outlier measurements, the consensus non-negative matrix factorization (cNMF) algorithm^[49]^, implemented in SynergyFinder, was used.

### Colony formation assay

Initially, 500 CRC cells were seeded per well in 6-well plates and incubated for 24 h. They were then exposed to compounds **5c** and **5e** at their respective IC_50_ and semi-IC_50_ concentrations. After 48 h, the media was replaced with fresh DMEM to remove remaining compounds. Cells were incubated for 10-12 days for colonies to form. Once formed, colonies were washed, fixed with 10% formalin for 15-20 min, then stained with 1% crystal violet in 10% ethanol. Excess dye was washed away, and the colonies were dried^[50,51]^. The stained and dried 6-well plates were scanned with a Canon ImageRUNNER Advance scanner. Colonies were counted and compared to the vehicle control (< 1% DMSO) to assess treatment effects.

### Cell cycle analysis

2 × 10^5^ HCT116 and DLD1 cells per well were seeded into 6-well dishes containing complete DMEM and cultured for 24 h. Subsequently, the cells were treated with compounds **5c** and **5e** at IC_50_ doses or a vehicle for 24 h. Cells were trypsinized, centrifuged, and fixed in 70% ethanol in PBS at 4 °C. The next day, the fixed cells were washed with PBS and incubated with FxCycle™ PI/RNase Staining Solution (ThermoFisher Scientific) for 10 min^[52]^. Flow cytometry was performed using a FACS Calibur flow cytometer (Becton, Dickinson), which captured 10,000 events per sample, and was evaluated using ModFit LT™ software (Verity Software House).

### Apoptosis assay

HCT116 and DLD1 lines were seeded at a density of 2 × 10^5^ cells per well into 6-well plates containing complete DMEM and allowed to grow for 24 h. Subsequently, the cells were treated with compounds **5c** and **5e** at their respective IC_50_ concentrations or with a vehicle control (< 1% DMSO) for 24 h. Post-treatment, the cells were detached using trypsinization, washed with PBS, and stained with the Annexin V-FITC Early Apoptosis Detection Kit (Cell Signaling Technology #6592) according to the supplier’s protocol. Finally, flow cytometry analysis was performed to assess apoptotic cells.

### Caspase 3/7 activation assay

10,000 CRC cells (HCT116 and DLD1) per well were plated into an opaque black plate with a clear bottom using complete culture media. After 24 h, the HCT116 and DLD1 cells were treated with compounds **5c** (0.8 and 0.6 µM, respectively) and **5e** (1.8 and 1.6 µM, respectively), and with an equivalent dose of DMSO (< 1% concentration) to the control group. After 48 h of incubation, Caspase-Glo® 3/7 reagent (Promega, G8090) was added as per the manufacturer’s instructions. Following an additional hour of incubation, luminescent readings were recorded using a plate reader.

### Western blots

HCT116 and DLD1 cells at 5 × 10^5^ cells per dish were seeded in 10 cm dishes for Western blot analysis. After 24 h of growth in complete DMEM medium, they were treated with vehicle (< 1% DMSO) or compounds **5c** and **5e** at IC_50_ for 48 h. The medium was removed, cells were washed with PBS, then lysed and sonicated in protease inhibitor-containing buffer. The histamine time-course experiment involved plating cells in 10-cm dishes and growing them until they reached 70%-80% confluence, then incubating them for 24 h in serum-free conditions. Cells were subsequently exposed to 10 μM histamine at time points of 1, 5, 15, 30, 60, and 120 min. To examine the impact of **Clac10** and **Clac12** on histamine signaling, we cultured HCT116 and DLD1 cells until they reached 70%-80% confluence, then incubated them for 24 h in serum-free media. Next, the cells were treated with **Clac10** and **Clac12** for 2 h, after which histamine was added for 15 min. At each time point, the reaction was stopped by removing the media, washing the cells with PBS, and collecting the cells by scraping. Lysates were centrifuged at 6,000 rpm for 10 min at 4 °C. Protein concentrations were measured with the bicinchoninic acid (BCA) assay. Equal protein samples were separated by sodium dodecyl sulfate - polyacrylamide gel electrophoresis (SDS-PAGE), transferred to polyvinylidene fluoride (PVDF) membranes at 90 V for 2 h, blocked with 5% skim milk in tris-buffered saline with Tween 20 (TBST) for 1 h, washed, and then incubated overnight at 4 °C with primary antibodies. After washing, membranes were incubated with secondary antibodies for 1 h, then washed again with TBST^[53]^. Protein bands were detected using enhanced chemiluminescence (ECL) chemiluminescence reagents (Cytiva, MA, USA). Protein band visualization was executed using the ChemiDoc-XRS+ system (Bio-Rad) and Image Lab software. The primary antibodies used here included MCL1 (Cat no. 94296S), BCL2 (Cat no. 4223), Bax (Cat no. 2772), B-cell lymphoma-extra large (BCL-XL; Cat no. 2762), p-GSK3B (Cat no. 9323), GSK3B (Cat no. 9315), phosphorylated-extracellular signal-regulated kinase (p-ERK; Cat no. 9101), extracellular signal-regulated kinase (ERK; Cat no. 4696), and cyclin D1 (Cat no. 2922S) from Cell Signaling Technology (Beverly, MA, USA); HRH1 antibody (Cat no. bs-6663R) from Bioss; HRH2 (Cat no. PA5142779) from ThermoFisher, and β-actin antibody (sc-47778) from Santa Cruz Biotechnology (Santa Cruz, CA, USA).

### Spheroid formation assay

To study the effects of compounds on CRC spheroids, single-cell suspensions of HCT116 and DLD1 cell lines were prepared and plated in ultralow attachment plates (Corning, Lowell, MA). Cells were seeded at a density of 5 × 10^2^ cells per well. The cells were cultured in a serum-free growth medium supplemented with epidermal growth factor (EGF; 20 ng/mL), fibroblast growth factor (FGF; 20 ng/mL), B27 (10 mL in 500 mL of 50X), heparin (4 µg/mL), and penicillin-streptomycin (1% v/v) (Invitrogen) to support spheroid formation^[54]^. After 2 days of incubation, spheroids formed and were then treated with either vehicle control (< 1% DMSO) or compounds **5c** and **5e** at their IC_50_ and half-IC_50_ concentrations. Spheroid growth was monitored, and spheroids were counted and imaged 5 days post-plating.

### Immunohistochemistry

Formalin-fixed CRC tissue samples were cut into 4 μm sections and mounted on slides. For immunohistochemistry (IHC), the slides were deparaffinized and then subjected to antigen retrieval. First, the tissue sections were blocked with UltraVision Hydrogen Peroxide Block (Thermo Scientific) for 10 min. Then, the slides were washed with PBS and incubated overnight at 4 °C in the dark using HRH1 antibody (Bioss, Cat#bs-6663R). The following day, the primary antibody was washed off, and the tissues were incubated with HRP Polymer Quanto for 10 min, then developed with a DAB Quanto Chromogen-Substrate mixture. The slides were finally counterstained with hematoxylin and eosin, fixed using mounting media, and examined using a Nikon Eclipse Ti microscope with a 20X objective. For immunofluorescence staining, the tissue sections were incubated overnight with Ki67 antibody (Invitrogen, Cat#53-5698-82) and Cleaved Caspase-3 antibody (Cell Signaling Technology, Cat#9661). The slides were washed three times with PBS for 10 min each. The Ki67 antibody, conjugated to Alexa Fluor™ 488, required no additional processing after washing. For Cleaved Caspase-3, slides were further incubated with Alexa Fluor™ 488 goat anti-rabbit secondary antibody (Invitrogen, Cat#A-11008). Both sets of slides were then stained with 4′,6-diamidino-2-phenylindole (DAPI; Thermo Fisher, Cat#62248) for nuclear visualization, prepared in a solution containing 0.3% bovine serum albumin (BSA) and 0.01% Triton X at a 1:500 dilution, and incubated for 2 h at room temperature in the dark. After three PBS washes, slides were mounted using aqueous-based mounting media (Fisher Scientific, Cat#50-247-04) and placed in a dark to dry. The slides were imaged using a Nikon 80i fluorescent microscope at 20X and 40X magnification.

### Molecular docking

The interaction of compounds **5c** and **5e** with the ligand-binding site of HRH1 (PDB ID: 3RZE)^[55]^ was studied using AutoDock Vina (Molecular Graphics Lab, Scripps Research Institute)^[56]^. AutoDock tools were employed, utilizing default settings to configure both proteins and ligands. The docking preparation included adding polar hydrogens and assigning Kollman and Gasteiger charges to both the HRH1 protein and the compounds. For the docking process, a 60 × 60 × 60 unit grid was set, centered on the ligand-binding region. The Lamarckian Genetic Algorithm (GA) was used to explore binding possibilities, generating approximately 10 distinct conformations per compound for detailed analysis. The conformations were evaluated based on binding affinity, selecting the ones with the lowest binding energy and the optimal number of hydrogen-bond interactions for further examination. Visualization and analysis of the most stable protein-ligand complexes were performed using PyMOL (https://pymol.org/2/)^[57]^, enabling detailed examination of interaction patterns and structural features.

### Cellular thermal shift assay

Cellular thermal shift assay (CETSA)^[58]^ was used to assess how compounds **5c** and **5e** affect the stability of HRH1 in CRC cells, specifically in the HCT116 and DLD1 cell lines, and *in vivo* HCT116 tumor xenograft tissues. Initially, the cells were cultured until they reached 70% to 80% confluency. Following growth, cell and tissue lysates were prepared using a lysis buffer. The HCT116 tumor xenograft tissues were first cut into small pieces and then homogenized in lysis buffer using a tissue homogenizer. Further, cell and tissue lysates were centrifuged at 4 °C for 10 min at 6,000 rpm. The lysates (at a concentration of 4 µg/µL) were then treated with either DMSO (as a control, < 1% concentration) or compounds **5c** and **5e** at 20 µM for 4 h. Post-treatment, the lysates were transferred to polymerase chain reaction (PCR) tubes and heated for 3 min at various temperature gradients to denature the proteins. This was followed by centrifugation for 20 min to separate the soluble fractions from the precipitated proteins. The supernatant was carefully collected, mixed with 2X Laemmli buffer, and then boiled at 70 °C for 10 min to ensure complete denaturation. A western blot was performed using an HRH1 antibody (Bioss, Cat#bs-6663R, 1:1,000 dilution) or an HRH2 antibody (ThermoFisher, Cat no. PA5142779).

### Tumor xenograft model

*In vivo* studies of compounds **5c** and **5e** used HCT116 xenografts in NSG mice, following University of Kansas IACUC guidelines and approved protocols (IACUC# 2021-2610). Male NSG mice aged 4-6 weeks were obtained from Jax Laboratory and maintained under standard conditions. To establish xenografts, 5 × 10^6^ HCT116 cells were injected subcutaneously into both flanks of the mice (*n* = 5/group). Once tumors (each mouse carrying 2 xenograft tumors, totaling 10 tumors per group) were palpable, mice were assigned to vehicle control and treatment groups, and compounds **5c** (**Clac10**) and **5e** (**Clac12**) were administered intraperitoneally at 20 mg/kg body weight once daily for 24 days. Tumor volumes were measured weekly with calipers. After the end of treatment, mice were euthanized, and tumors excised, weighed, and analyzed histologically, immunohistochemically, and by western blot to assess structural and molecular effects^[50,59]^.

### Statistical analysis

All data values are given either as mean ± standard deviation (SD) or mean ± standard error of the mean (SEM). Compared with the control group, Experimental data were analyzed using ordinary one-way or two-way analysis of variance (ANOVA). A *P*-value of less than 0.05 was considered significant.

## RESULTS

### Chemistry

**Scheme 2:**
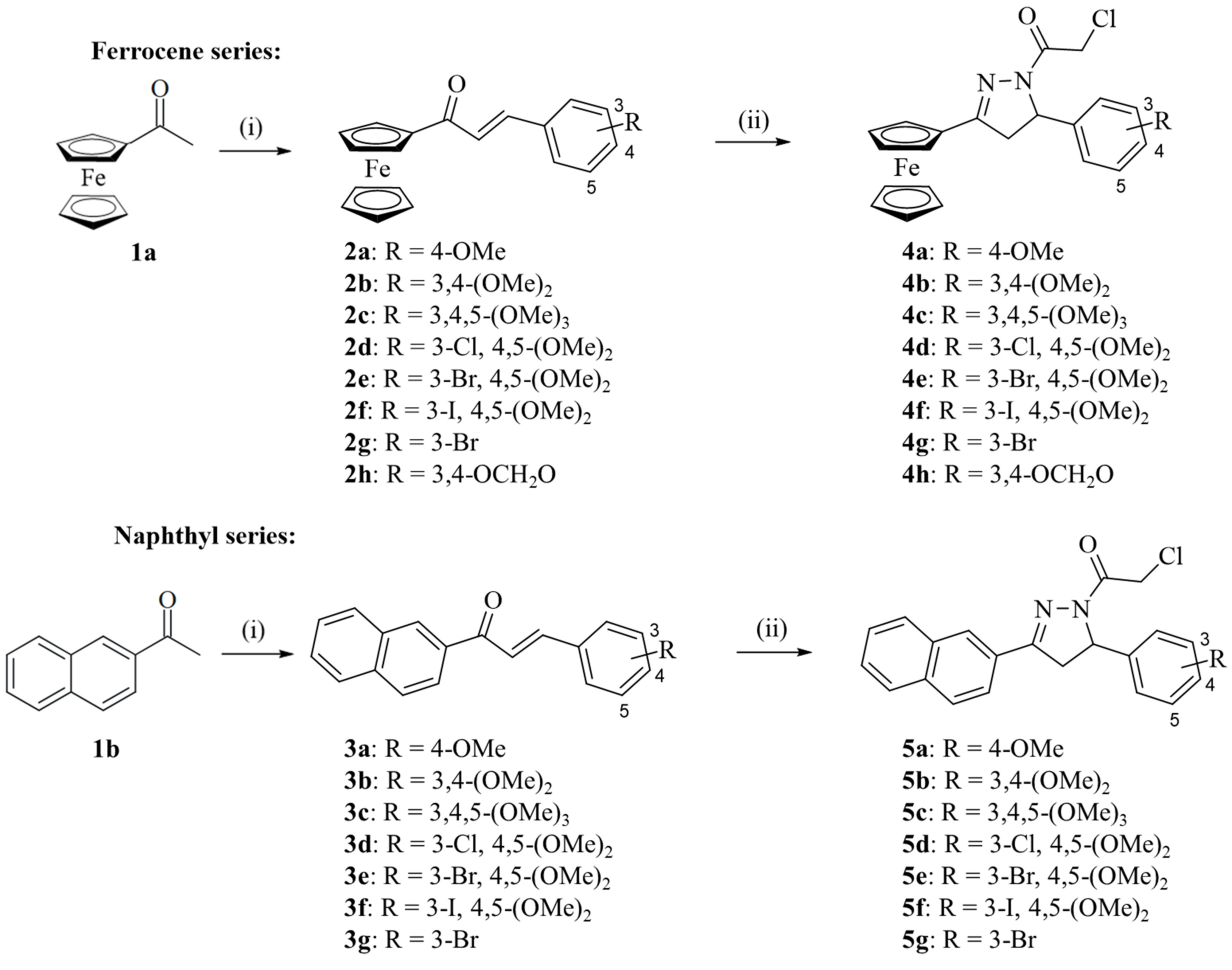
Reagents and conditions: (i) NaOH, H_2_O, EtOH, r.t., 24 h, 63%-77%; (ii) N_2_H_4_ x H_2_O, chloroacetic acid, reflux, 2-3 h, 14%-24% for **4a-h**, 30%-59% for **5a-g**.

The previously described compound **5a**^[43]^ and the new racemic 2-pyrazolines **4a-h** and **5b-g** were prepared from the corresponding chalcones **2a-h** and **3a-g** treated in one pot with hydrazine hydrate and chloroacetic acid. The chalcone precursors **2a-h** were obtained from acetylferrocene (**1a**), and chalcones **3a-g** from 2-acetylnaphthalene (**1b**) and the respective aryl aldehydes by Claisen-Schmidt condensation [Scheme 2]^[32,33,37,40]^. In general, the yields of the ferrocene derivatives **4a-h** were lower than those of the naphthalene derivatives **5a-g**. The low yields of the ferrocene-based 2-pyrazolines may be attributed to the degradation of the ferrocene moiety under the harsh reaction conditions described, i.e., in a mixture of hot chloroacetic acid and hydrazine hydrate [Supplementary Materials]. The new 2-pyrazolines **4** and **5** were analyzed by NMR, IR and mass spectrometry (MS) techniques. ^1^H NMR spectra of the 2-pyrazolines displayed the characteristic ABX pattern of 3,5-diaryl-substituted 2-pyrazoline ring protons and the methylene signal of the chloroacetamide moiety^[60]^. ESI-HRMS spectra showed the molecular peaks of the target compounds plus one proton (M^+^ + H) [Supplementary Figures 3-40].

### Cell viability and colony formation assays

The compounds were initially tested for their ability to inhibit the growth of HCT116 (p53-WT) and HCT116^-/-^ (p53-KO) cells, as well as DLD1 CRC cells [Table 1]. Compounds with 3,4,5-trimethoxyphenyl (**4c, 5c**) or 3-bromo-4,5-dimethoxyphenyl (**5e**) substituents showed the strongest activity against CRC cell lines, with **5c** and **5e** reaching nanomolar IC_50_ values in DLD1 cells. In contrast, anisyl (**4a, 5a**), 3,4-methylenedioxyphenyl (**4h**), and 3-bromophenyl (**4g, 5g**) derivatives were less active. Overall, 3,4-dimethoxy, 3,4,5-trimethoxy, and 3-halo-4,5-dimethoxy patterns were most effective. But also, the naphthalene and ferrocene rings play a role in antiproliferative activity. A considerable difference in activity was observed for the couple of **4e** and **5e**, and naphthyl **5e** was distinctly more active than its close ferrocene analog **4e**. Slight differences were observed among the 3-halo-4,5-dimethoxyphenyl derivatives. Among the ferrocenes, the 3-chloro substituent of **4d** was superior to the 3-bromo and 3-iodo derivatives **4e** and **4f**, while the 3-bromo-4,5-dimethoxy analog **5e** was superior to the 3-chloro and 3-iodo analogs **5d** and **5f** among the naphthalene derivatives. Notably, compounds **4c**, **5c,** and **5e** were likewise active against the p53-KO HCT116 cells, indicating a p53-independent mechanism of action for these compounds and a key attribute for overcoming drug resistance due to impaired p53 activity^[61]^. In contrast, the 3-chloro/bromo derivatives **4d** and **4e** were distinctly less active against the p53-KO cells, indicating a disadvantage of 3-halogen substitution in the ferrocene series here. Remarkably, **4e** displayed by far the lowest activity against the HCT116 p53-KO cells of all test compounds, while the wild-type HCT116 cells and the DLD1 cells were distinctly more sensitive to treatment with **4e**. Additionally, the DLD1 cells were often slightly more sensitive to 2-pyrazoline treatment than the HCT116 cells. It is noteworthy that the 3-iodo derivative **4f** was more active than the 3-bromo analog **4e** against DLD-1. Naphthyl **5c** and its ferrocene analog **4c** were slightly more active against HCT116 cells than the best *N*-acetyl analog published by us before (a naphthalene-based 3-iodo-4,5-dimethoxyphenyl derivative, IC_50_ = 1.6 µM), while the activity of **5e** was identical to that of the known analog in these cells^[32]^. Notably, the analogous 3-iodo-4,5-dimethoxyphenyl derivative **5f** was slightly less active against HCT116 cells.

**Table 1:** Inhibitory concentrations IC_50_ (in µM) of compounds 4a-h and 5a-g when applied to HCT116 and DLD1 (colon cancer) cell lines at 72 h

**Compounds**	**HCT116**	**DLD1**	**HCT116 (p53^-/-^)**
**4a**	3.50 ± 1.75	2.03 ± 0.64	4.5 ± 0.3
**4b**	2.20 ± 0.71	1.43 ± 0.45	2.2 ± 0.2
**4c**	1.40 ± 0.06	1.33 ± 0.29	1.8 ± 0.06
**4d**	2.30 ± 0.51	1.30 ± 0.31	5.4 ± 0.4
**4e**	3.13 ± 0.69	3.30 ± 0.64	11.4 ± 1.0
**4f**	3.27 ± 0.12	1.93 ± 0.34	-
**4g**	4.57 ± 0.72	2.53 ± 1.01	-
**4h**	3.93 ± 1.39	4.07 ± 2.08	-
**5a**	5.10 ± 1.20	3.50 ± 1.20	-
**5b**	2.83 ± 0.75	1.03 ± 0.35	2.2 ± 0.2
**5c** (**Clac10**)	1.27 ± 0.27	0.90 ± 0.38	1.5 ± 0.04
**5d**	2.50 ± 0.56	1.37 ± 0.43	2.6 ± 0.06
**5e** (**Clac12**)	1.60 ± 0.29	0.93 ± 0.15	1.3 ± 0.04
**5f**	2.53 ± 0.58	1.80 ± 0.12	2.5 ± 0.06
**5g**	5.33 ± 1.53	2.87 ± 0.70	-
**Oxaliplatin**	1.10 ± 0.13	1.03 ± 0.08	-
**5-FU**	1.23 ± 0.38	1.60 ± 0.62	-

Values are the means of at least 3 independent experiments (± SD). 5-FU and oxaliplatin were used as a positive control. IC_50_: Half maximal inhibitory concentration; 5-FU: 5-fluorouracil; SD: standard deviation.

Compounds **4a-e** and **5b-f** were further selected to assess their antiproliferative activity against a panel of non-CRC cancer cell lines (melanoma, breast carcinoma, cervix carcinoma, and glioblastoma cells) to determine their broad-spectrum anticancer activity [Table 2]. **4c**, **5c** and **5e** were among the most active compounds again underlining the beneficial effects of 3,4,5-trimethoxy and 3-bromo-4,5-dimethoxy substitution patterns in these cancer cell lines, too. Yet, the 3-chloro and 3-iodo analogs **5d** and **5f** of the naphthalene series also exhibited considerable activities and were more active than **5c** and **5e** against specific cell lines (e.g., 518A2 and U87 cells). The U87 glioblastoma cells were particularly sensitive, with IC_50_ values reaching the nanomolar concentration range. In addition, **5c** was four times more active against vinblastine-resistant KB-V1(Vbl) cervix carcinoma cells, which overexpress the drug-efflux ABC transporter P-gp, than against cells of the ancestral cell line HeLa. Treatment of KB-V1(Vbl) cells with the P-gp inhibitor verapamil only slightly sensitized cells to some compounds (e.g., **4b** and **4c**) by a factor of 2, while the activity of **5c** and **5e** remained unchanged. Thus, the activity of **5c** and **5e** against KB-V1(Vbl) cells appeared to be independent of P-gp, which is essential for developing **5c** and **5e** as anticancer agents. In comparison with the *N*-acetyl 2-pyrazoline compounds from our previous study, the new ferrocenes **4c** and **4d** and the naphthalene-based 3-iodo-4,5-dimethoxyphenyl derivative **5f** appeared to be more active against 518A2 melanoma cells^[32]^.

**Table 2:** Inhibitory concentrations IC_50_ (in µM) of test compounds 4a-e and 5b-f when applied to 518A2 melanoma, HeLa, and vinblastine-resistant KB-V1(Vbl) cervix carcinoma, U87 glioblastoma, and MCF-7 breast carcinoma cells^a^

**Compound**	**518A2**	**HeLa**	**KB-V1(Vbl)**	**KB-V1(Vbl)^b^**	**U87**	**MCF-7**
**4a**	5.1 ± 0.06	5.1 ± 0.1	8.2 ± 0.3	5.1 ± 0.1	-	-
**4b**	4.6 ± 0.2	3.0 ± 0.08	5.7 ± 0.1	2.3 ± 0.3	-	-
**4c**	1.7 ± 0.2	1.9 ± 0.09	6.1 ± 0.3	2.7 ± 0.2	-	-
**4d**	1.6 ± 0.08	4.9 ± 0.1	5.5 ± 0.4	6.0 ± 0.3	-	-
**4e**	5.0 ± 0.6	5.7 ± 0.3	9.8 ± 0.4	6.5 ± 0.3	-	-
**5b**	3.3 ± 0.5	7.2 ± 0.7	5.6 ± 0.9	3.0 ± 0.04	1.6 ± 0.08	5.1 ± 0.06
**5c** (**Clac10**)	2.5 ± 0.3	4.9 ± 0.3	1.2 ± 0.3	1.7 ± 0.1	0.6 ± 0.1	1.6 ± 0.2
**5d**	2.4 ± 0.2	2.1 ± 0.1	2.0 ± 0.2	5.0 ± 0.2	0.4 ± 0.03	-
**5e** (**Clac12**)	2.3 ± 0.2	2.3 ± 0.2	2.4 ± 0.2	2.4 ± 0.2	1.4 ± 0.06	2.1 ± 0.1
**5f**	1.4 ± 0.02	5.9 ± 0.2	5.5 ± 0.2	5.0 ± 0.04	0.7 ± 0.07	-

^a^Values are the means of at least three experiments (± SD). ^b^Cells treated with P-gp inhibitor verapamil. IC_50_: Half maximal inhibitory concentration; SD: standard deviation; P-gp: P-glycoprotein.

**Figure 1:**
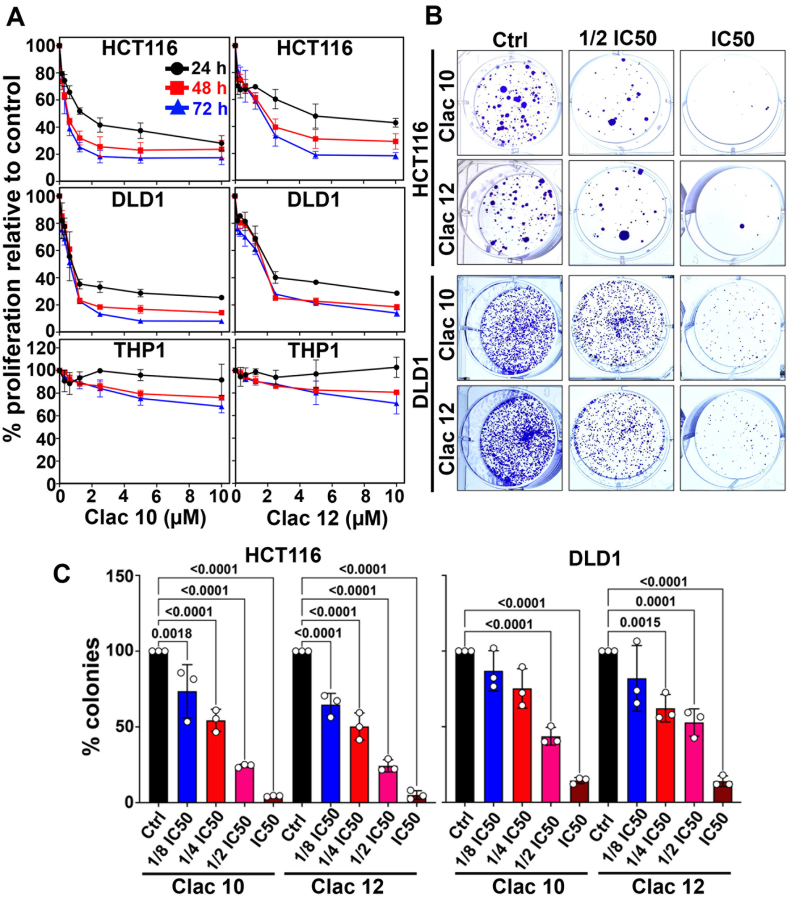
**Clac10** (**5c**) and **Clac12** (**5e**) inhibit proliferation and colony formation in CRC cell lines HCT116 and DLD1. (A) Cells treated with 0-10 μM of each compound for up to 72 h showed a dose- and time-dependent reduction in proliferation, indicating antiproliferative effects; (B) Cells treated with various concentrations of **5c** (**Clac10**) and **5e** (**Clac12**) for 48 h, followed by 10-14 days of culture in compound-free media, revealed that both compounds significantly reduced colony formation, indicating suppressed clonogenic potential; (C) Quantification confirmed a significant decrease in colony numbers in treated cells compared with vehicle-treated cells. The data were expressed as mean ± SD and analyzed by one-way ANOVA. CRC: Colorectal cancer; SD: standard deviation; ANOVA: analysis of variance; IC_50_: half maximal inhibitory concentration.

Moreover, compounds **5c** and **5e** showed dose- and time-dependent cytotoxicity against the HCT116 and DLD1 cells [Figure 1A]. These compounds began to exhibit cytotoxicity at 24 h, showing a significant increase in activity at 48 h, and reached maximum effectiveness at 72 h. Notably, compounds **5c** and **5e** did not inhibit the growth of non-cancerous THP-1 monocyte cells [Figure 1A], Jurkat (a human T-lymphocyte cell), and Het-1A (an immortalized human esophageal epithelial cell line) (IC_50_ > 10 µM) [Supplementary Figure 1], indicating selective cytotoxicity towards tumor cells. We utilized the IC_50_ doses of compounds **5c** and **5e** at 48 h for subsequent experiments. The long-term effects of these compounds on the clonogenic potential of CRC cells (HCT116 and DLD1) were evaluated using the colony formation assay. Both compounds significantly reduced colony size and number in CRC cell lines [Figure 1B and C], demonstrating an irreversible anticancer effect. These findings indicate that compounds **5c** (**Clac10**) and **5e** (**Clac12**) exert strong antineoplastic effects by not only inhibiting cell proliferation but also impairing CRC cells’ ability to form new colonies. This suggests their cytotoxic effects on colon cancer cells are long-lasting and may help control cancer recurrence, highlighting their potential as therapeutic agents in cancer treatment.

To evaluate the effectiveness of **Clac10** and **Clac12** in combination with the chemotherapeutic drug 5-FU^[62]^ for treating CRC, we assessed their antiproliferative effects on CRC cells across 49 different combinations at 0-5 μM doses. Our findings indicate that, individually, 5-FU, **Clac10**, and **Clac12** exhibited dose-dependent antiproliferative activity against HCT116 and DLD1 cells. When combined with 5-FU, both **Clac10** and **Clac12** significantly reduced CRC cell proliferation compared with the individual compounds alone, demonstrating synergistic effects. Analysis of these combination effects using Synergy Finder suggested the most potent synergy at doses of 0.16-0.62 µM of **Clac10** and 0.16-0.62 µM of 5-FU in HCT116 cells, as well as 0.07-0.31 µM of **Clac12** and 0.16-0.62 µM of 5-FU in DLD1 cells [Supplementary Figures 2 and 3]. These results suggest that combining **Clac10** and **Clac12** with 5-FU could offer a more effective treatment strategy for CRC, warranting further investigation into their clinical applications.

To evaluate the hydrolytic stability of **Clac10** and **Clac12**, we conducted hydrolysis assays in PBS and monitored the compounds by ultraviolet (UV)-visible spectroscopy. The results showed that both **Clac10** and **Clac12** exhibited minimal hydrolysis over 24 h, as evidenced by a slight decrease in the intensity of their characteristic absorption bands. This suggests that **Clac10** and **Clac12** (designated as **5c** and **5e**, respectively) are stable under the tested conditions [Supplementary Figure 4]. The significance of these findings lies in the stability of these compounds under physiological conditions, indicating their potential suitability for further *in vivo* studies and therapeutic applications.

### Cell cycle arrest and apoptosis

**Figure 2:**
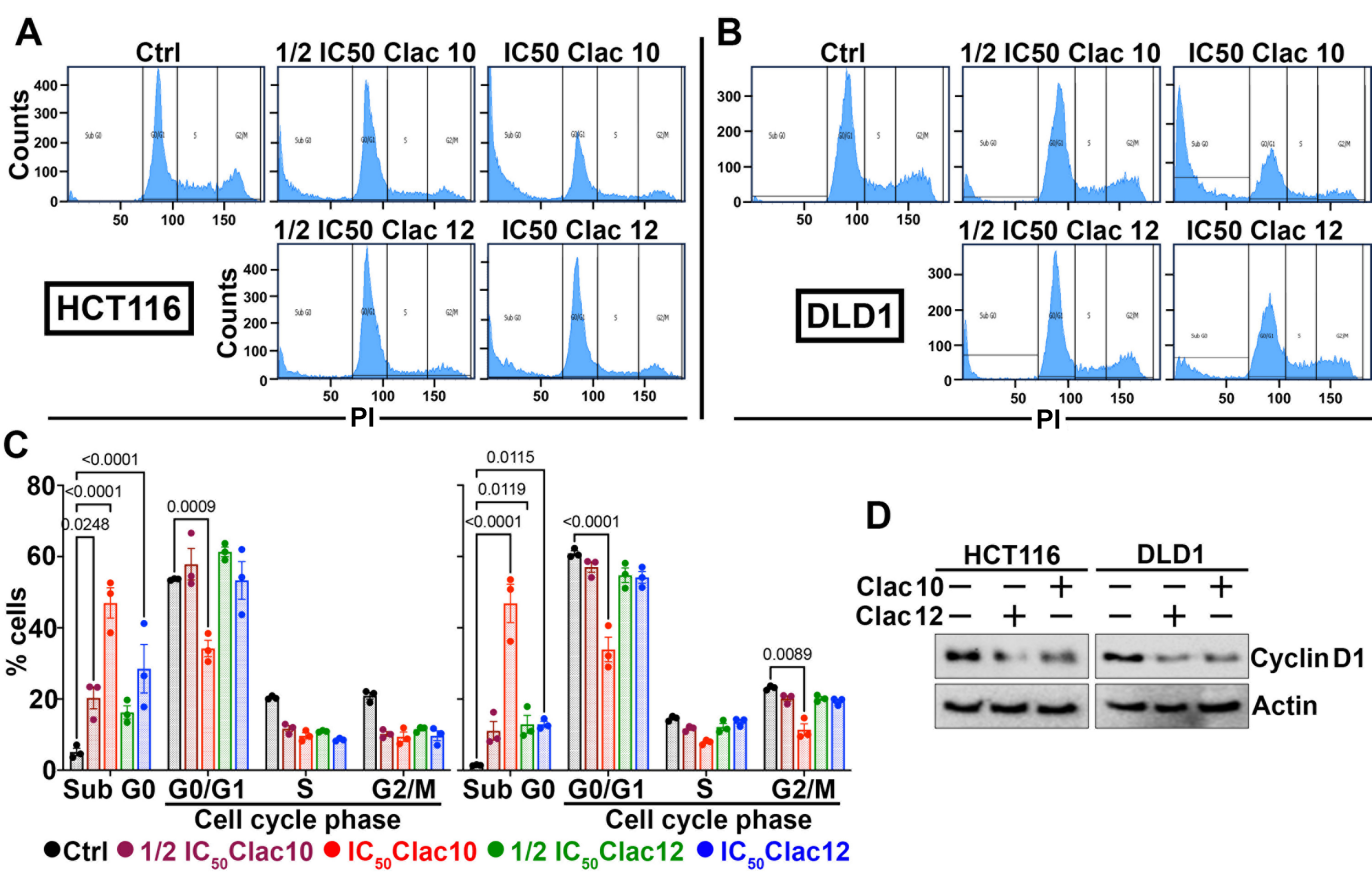
**Clac10** (**5c**) and **Clac12** (**5e**) cause cell cycle arrest in CRC cells. (A) HCT116 and (B) DLD1 cells were treated with IC_50_ and ½IC_50_ concentrations of **5c** (**Clac10**) and **5e** (**Clac12**) for 48 h and analyzed by flow cytometry after propidium iodide staining for DNA content; (C) The quantification figure shows that both compound treatments led to cell cycle arrest, as evidenced by an increase in the Sub-G0 cell population; (D) Lysates from HCT116 and DLD1 cells treated with IC_50_ concentrations of **5c** (**Clac10**) and **5e** (**Clac12**) for 48 h were examined by western blotting. Both compounds significantly reduced cyclin D1 expression. Data is presented as mean ± SD. An ordinary two-way ANOVA was used for statistical comparison. CRC: Colorectal cancer; IC_50_: half maximal inhibitory concentration; SD: standard deviation; ANOVA: analysis of variance; PI: propodium iodide.

**Figure 3:**
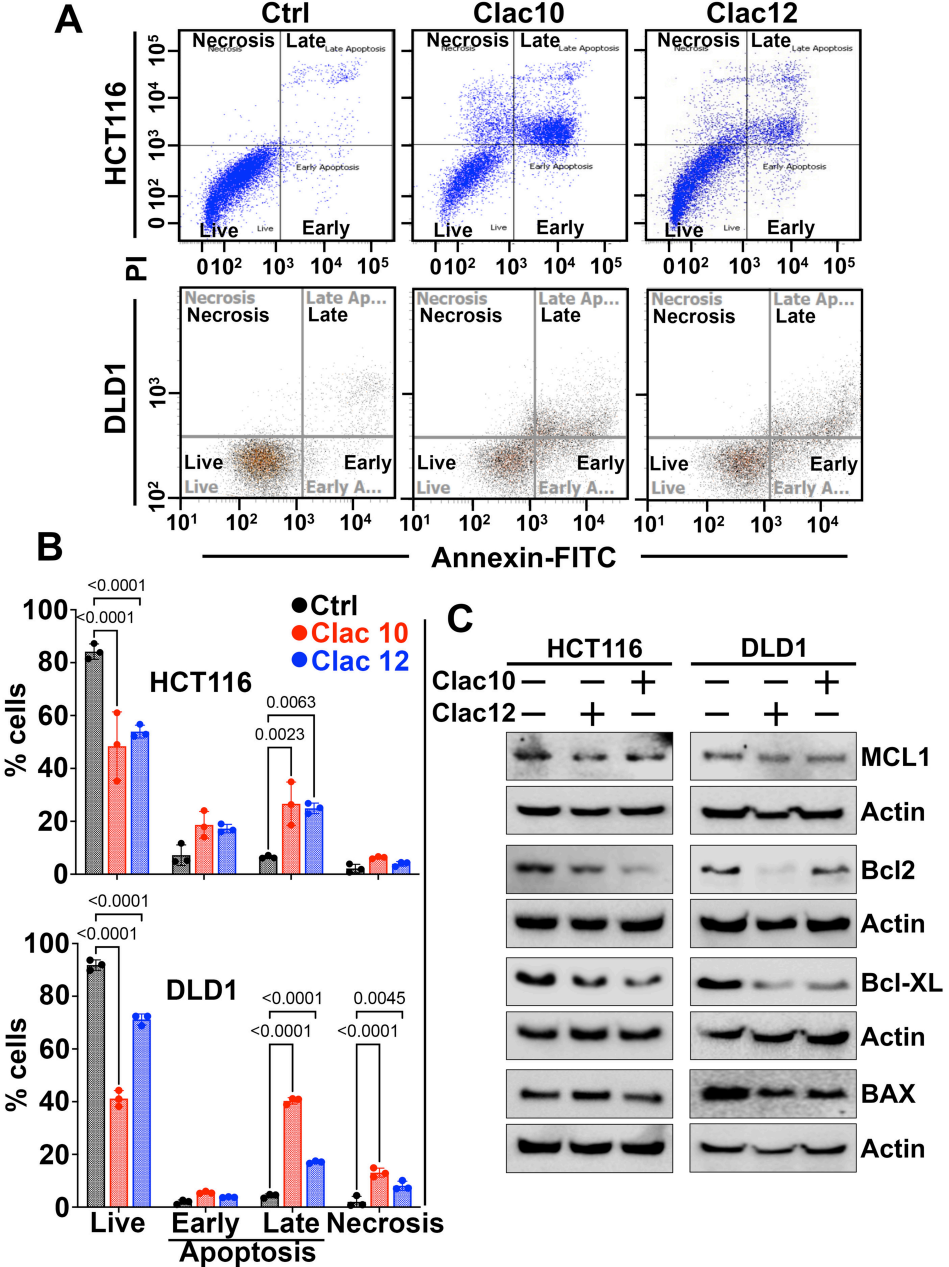
**Clac10** (**5c**) and **Clac12** (**5e**) trigger apoptosis. (A) HCT116 and DLD1 cells were treated with the IC_50_ concentration of **Clac10** (**5c**) and **Clac12** (**5e**) for 48 h, stained with Annexin V (FITC) and PI, and analyzed by flow cytometry; (B) Quantification shows that both compounds significantly induced late apoptosis in HCT116 and DLD1 cells; (C) Lysates from HCT116 and DLD1 cells treated with **Clac10** (**5c**) and **Clac12** (**5e**) showed reduced levels of anti-apoptotic marker proteins BCL-XL, MCL-1, and BCL-2 compared to untreated controls. Data is presented as mean ± SD. An ordinary two-way ANOVA was used for statistical comparison. IC_50_: Half maximal inhibitory concentration; FITC: fluorescein isothiocyanate; PI: propidium iodide; BCL-XL: B-cell lymphoma-extra large; MCL-1: myeloid cell leukemia 1; BCL-2: B-cell lymphoma 2; SD: standard deviation; ANOVA: analysis of variance; BAX: BCL2-associated X protein.

Compounds **5c** and **5e** were selected for further experiments. Next, we examined whether the suppression of cell growth by compounds **5c** and **5e** was due to their effects on cell cycle progression. We treated HCT116 and DLD1 cells with compounds **5c** and **5e** and analyzed them using flow cytometry. Our findings revealed a substantial increase in cell numbers in the sub-G0 phase, suggesting cell-cycle disruption [Figure 2A-C]. Since cyclin D1 is essential for cell cycle progression, we examined its expression. We found that the expression of cyclin D1 was suppressed in both CRC cell lines after treatment with compounds **5c** and **5e** [Figure 2D and Supplementary Figure 5]. These datasets suggest that compounds **5c** and **5e** induced cell cycle arrest in CRC cells, thereby augmenting their growth. The increase in cells in the sub-G0 stage, due to fragmented DNA, suggests that compounds **5c** and **5e** exhibit cytotoxicity. Further, the induction of apoptosis by **5c** and **5e** was studied in HCT116 and DLD1 CRC cells using the Annexin-PI assay via flow cytometry at the IC_50_ concentration at the 48-hour time point. **5c** and **5e** induced late apoptosis in HCT116 cells, whereas in DLD1 cells, late apoptosis and a distinct necrotic effect were observed [Figure 3A and B]. Consequently, viable CRC cells were reduced upon treatment with 2-pyrazoline derivatives **5c** and **5e**. As another indicator of apoptosis induction, the activation of caspases 3 and 7 by complexes **5c** and **5e** was studied [Supplementary Figure 6]. Both compounds **5c** (0.8 and 0.6 µM, respectively) and **5e** (1.8 and 1.6 µM, respectively) exhibited ~3-4-fold increase in caspase 3 and 7 activity in HCT116 and DLD1 cells. Mechanistically, both **5c** and **5e** suppressed anti-apoptotic MCL1, BCL2, and BCL-XL protein^[63]^ expression in HCT116 and DLD1 cells, while pro-apoptotic BAX expression remained unchanged or slightly reduced when compared with untreated cells [Figure 3C and Supplementary Figure 7]. These datasets suggest that these compounds induced apoptosis in CRC cells by inhibiting the expression of antiapoptotic proteins.

### Spheroid formation assay

**Figure 4:**
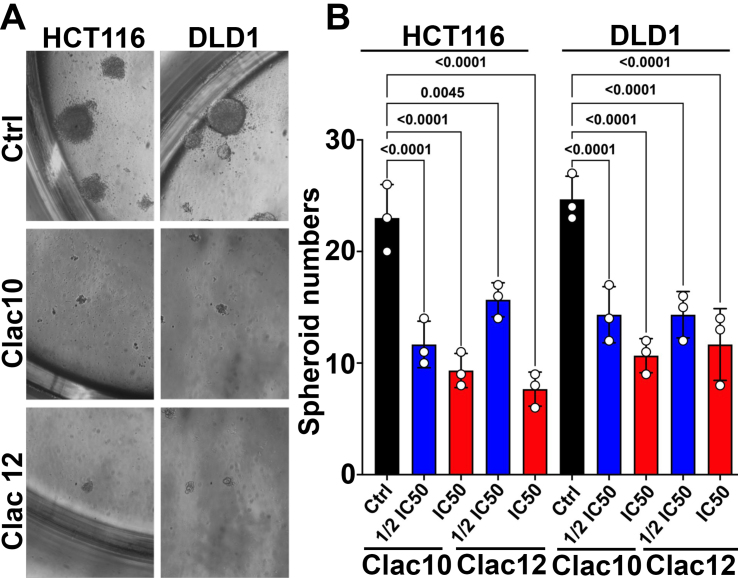
**Clac10** (**5c**) and **Clac12** (**5e**) suppress spheroid formation. (A) HCT116 and DLD1 cells were cultured in specialized spheroid media in ultra-low attachment plates and treated with IC_50_ and ½IC_50_ concentrations of **Clac10** (**5c**) and **Clac12** (**5e**). The spheroids were imaged at 10X magnification. After 5 days, colonospheres were imaged at 10X magnification; (B) Spheroid count from the spheroid formation assay. **Clac10** (**5c**) and **Clac12** (**5e**) treatments significantly reduced the number of colonospheres. Data is shown as mean ± SD. An ordinary one-way ANOVA was used for statistical analysis. IC_50_: Half maximal inhibitory concentration; SD: standard deviation; ANOVA: analysis of variance.

CSCs are known to form spheroids in ultra-low attachment plates, making spheroid formation assays^[64]^ a valuable tool for assessing the impact of treatments on CSCs. We used this assay to evaluate the effects of compounds **5c** and **5e** on CRC CSCs and found that both compounds significantly inhibited spheroid formation, both in size and number, in CRC cells [Figure 4A and B]. These datasets suggest that compounds **5c** and **5e** impede the growth of both proliferating and CSCs and can be used to overcome tumor recurrence, inhibit drug resistance, and tumor progression.

### HRH1 is upregulated in CRC, and compounds 5c and 5e target it to inhibit histamine-induced downstream signaling

**Figure 5:**
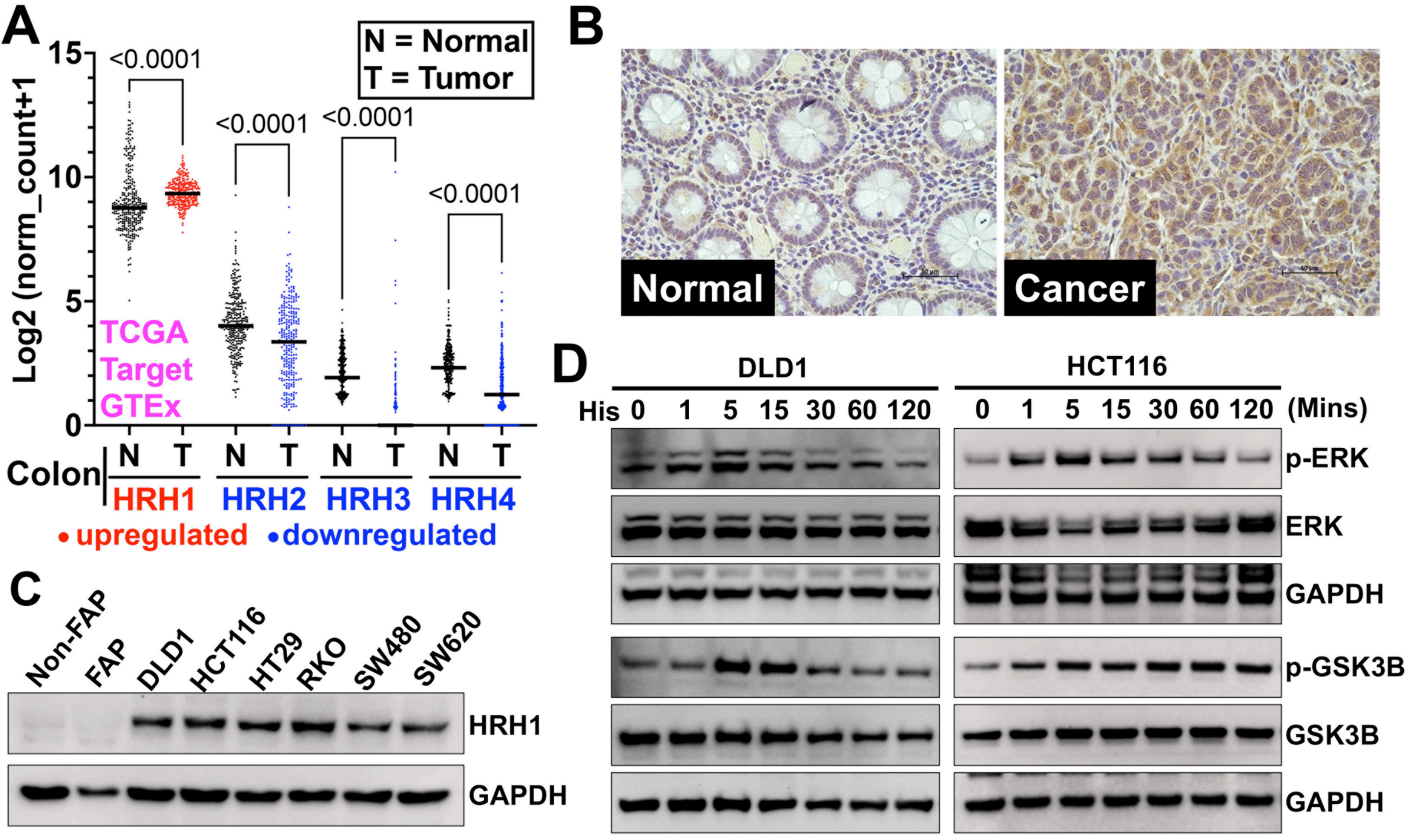
HRH1 is overexpressed and functional in CRC tissues and cell lines. (A) We analyzed HRH1-HRH4 gene expression in colon cancer patient tissues from the TCGA and TARGET databases using the Xena browser. We then compared it to adjacent or normal colon tissues from the TCGA and GTEx databases. HRH1 expression was significantly upregulated, while HRH2-H4 expression was significantly downregulated in colon cancer tissues compared to adjacent normal colon tissues. Ordinary one-way ANOVA was used for statistical analysis; (B) Immunohistochemistry of a CRC tumor microarray (adjacent Normal = 15, colon adenocarcinoma = 15 tissues), obtained from Tissue microarray cat no. CO243b and BRCF of KUMC showed overexpression of HRH1 in colon cancer compared to its adjacent normal colonic tissue (scale bar = 50 µm); (C) Western blot analysis revealed that HRH1 was upregulated in CRC cell lines compared to non-cancerous colonic cells. HCT116 and DLD1 were selected for further study due to their high expression levels among the tested CRC cell lines; (D) Western blot of lysates from CRC cells, previously synchronized in serum-free media for 24 h, treated with 10 µM of histamine for up to 2 h in serum-free media, showed a time-dependent increase in ERK1/2 (Thr202/Tyr204) and GSK3B (Ser-9) phosphorylation. HRH1: Histamine receptor H1; CRC: colorectal cancer; HRH4: Histamine receptor H4; ANOVA: analysis of variance; BRCF: Biospecimen Repository Core Facility; KUMC: University of Kansas Medical Center; ERK1/2: extracellular signal-regulated kinase 1/2; GSK3B: glycogen synthase kinase 3 beta; p-ERK: phosphorylated-extracellular signal-regulated kinase; p-GSK3B: phosphorylated-glycogen synthase kinase 3 beta; GAPDH: glyceraldehyde-3-phosphate dehydrogenase.

We first analyzed the gene expression of HRH1-HRH4 in CRC patient tissues using the Xena browser^[65]^. We compared HRH1-HRH4 expression in CRC patients from the TCGA-TARGET database with that in adjacent normal colonic tissues from the TCGA and GTEx databases. We found that only HRH1 is overexpressed in CRC tissues, while Histamine receptor H2-H4 are downregulated in CRC compared to adjacent normal tissues [Figure 5A]. Next, we examined HRH1 expression in CRC tissues by immunohistochemistry. We observed higher HRH1 expression in CRC tissues compared with adjacent normal colonic tissue [Figure 5B]. Next, we examined HRH1 expression in CRC cell lines using western blot analysis. We found higher HRH1 expression in CRC cell lines compared to adjacent normal colonic cells [Figure 5C]. Histamine binds to histamine receptors, activating downstream signaling in cancer cells to promote cancer progression^[66,67]^. Hence, to understand the receptor’s functionality, we treated high HRH1-expressing CRC cells (HCT116 and DLD1) with histamine and observed a time-dependent increase in the phosphorylation of GSK3B (Ser-9) and ERK (Thr202/Tyr204) [Figure 5D and Supplementary Figure 8]. These data suggest that HRH1 is overexpressed in CRC cells and tissues, and its activation by histamine induces downstream signaling involved in CRC proliferation and tumor progression.

**Figure 6:**
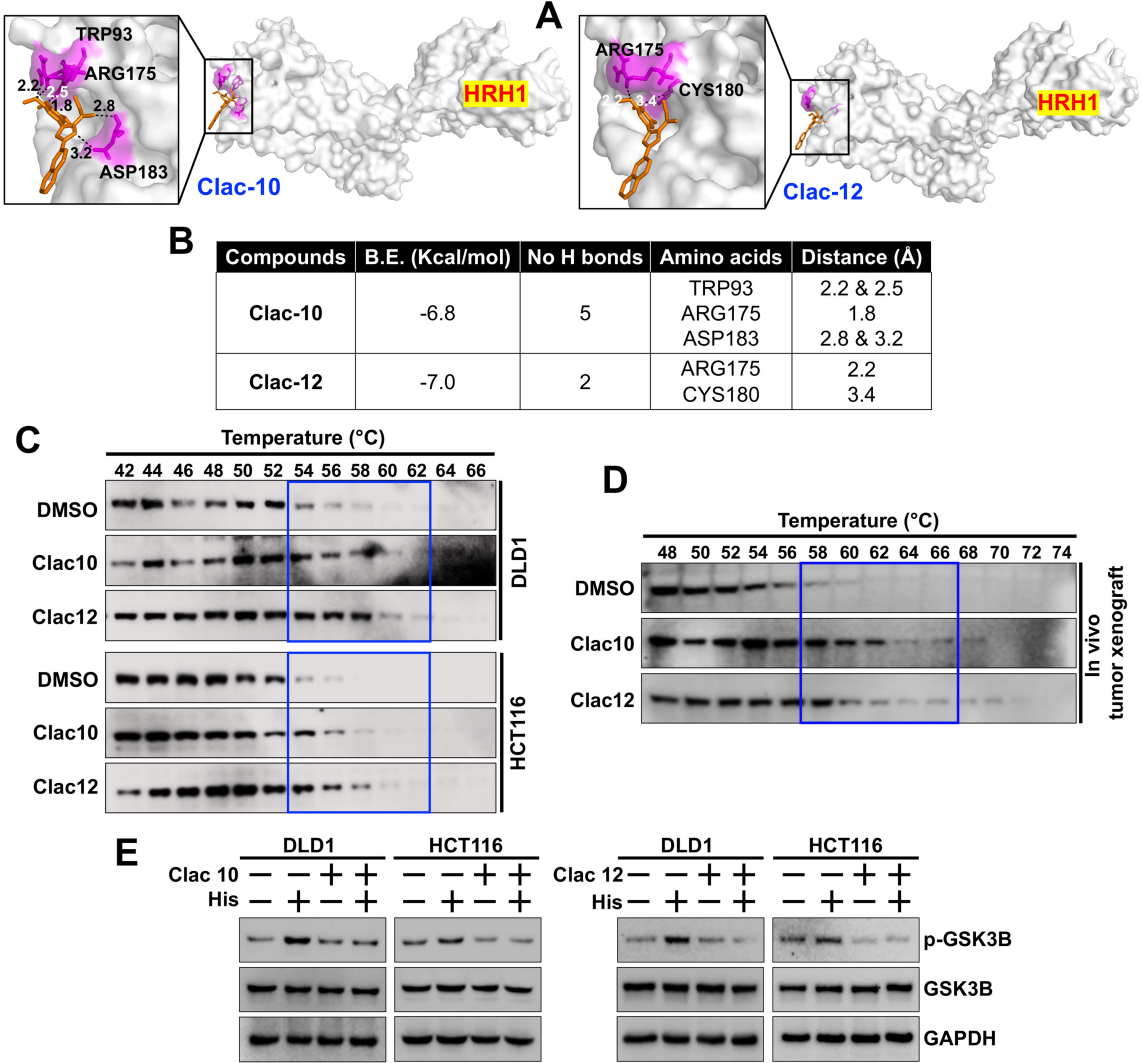
**Clac10** (**5c**) and **Clac12** (**5e**) bind to HRH1. (A) Molecular docking was used to assess the binding of **5c** (**Clac10**) and **5e** (**Clac12**) to the HRH1 protein cavity. Both compounds bind to the protein with binding energies of -6.8 and -7.0 kcal/mol, respectively. The figure shows cartoon and surface models for **5c** (**Clac10**) and **5e** (**Clac12**); (B) A summary of the docking results and consensus scores for **5c** (**Clac10**) and **5e** (**Clac12**) binding to HRH1 is provided; (C) The CETSA shows that **5c** (**Clac10**) and **5e** (**Clac12**) stabilize the HRH1 protein, suggesting potential binding; The compounds were incubated with cell lysates from HCT116 and DLD1 cell lines (C) or HCT116 Tumor xenograft lysates (D) for 4 h, followed by thermal denaturation and western blot analysis; (E) Preincubation of **5c** (**Clac10**) and **5e** (**Clac12**) inhibits histamine-induced GSK3B phosphorylation in CRC cells. HRH1: Histamine receptor H1; CETSA: cellular thermal shift assay; GSK3B: glycogen synthase kinase 3 beta; CRC: colorectal cancer; DMSO: dimethyl sulfoxide; p-GSK3B: phosphorylated-glycogen synthase kinase 3 beta; GAPDH: glyceraldehyde-3-phosphate dehydrogenase.

To understand if pyrazoline analogs can target HRH1, we used molecular docking to determine whether compounds **5c** (**Clac10**) and **5e** (**Clac12**) bind to HRH1 with the Autodock Vina software. The docking analysis predicted that both compounds interact with the extracellular domain (ECD) of HRH1 with binding energies of -6.8 and -7.0 kcal/mol, respectively, and stabilize themselves by forming hydrogen bonds with key amino acid residues [Figure 6A and B]. **Clac10** interacts with two ECDs of HRH1 by forming hydrogen bonds with TRP93, ARG175, and ASP183, while **Clac12** interacts with ARG175 and CYS180, which belong to a single ECD of HRH1 [Figure 6B]. Therefore, we propose that **Clac10** will be a more effective HRH1 antagonist. Blocking the histamine binding site on HRH1 with compounds **5c** or **5e** may prevent histamine-HRH1-mediated downstream signaling, thereby inhibiting tumor progression. Next, we performed a CETSA in CRC cells (HCT116 and DLD1) and *in vivo* HCT116 xenograft tissues to confirm the binding of compounds **5c** or **5e** to HRH1 protein. CETSA showed that the HRH1 protein began to denature at 54 °C, while compounds **5c** and **5e** stabilize HRH1 and provide protection against thermal denaturation up to 58 °C (~4 °C), indicating potential binding of these compounds to HRH1 [Figure 6C and D]. Furthermore, to understand the selectivity of compounds **5c** and **5e** for binding with HRH1 *vs.* HRH2, we performed CETSA combined with a western blot for HRH2 in CRC cells (HCT116 and DLD1) and HCT116 xenograft tissues. We observed HRH2 denaturation at 60 °C, while compounds **5c** and **5e** did not stabilize HRH2 against thermal denaturation, indicating no binding with HRH2 [Supplementary Figure 9]. Next, to assess how compounds **5c** and **5e** bind to HRH1 and block histamine-induced downstream signaling, we pre-treated CRC cells with these compounds and then exposed them to histamine. The pretreatment with compounds **5c** and **5e** (for 4 h) inhibited histamine-induced phosphorylation of GSK3B (measured at 15 min)v in HCT116 and DLD1 cells [Figure 6E and Supplementary Figure 10]. This data confirms that compounds **5c** and **5e** bind to the ECD of HRH1 and thus prevent histamine from binding to HRH1 on cancer cells, thereby inhibiting downstream signaling in CRC cells.

### Tumor xenograft model in mice

**Figure 7:**
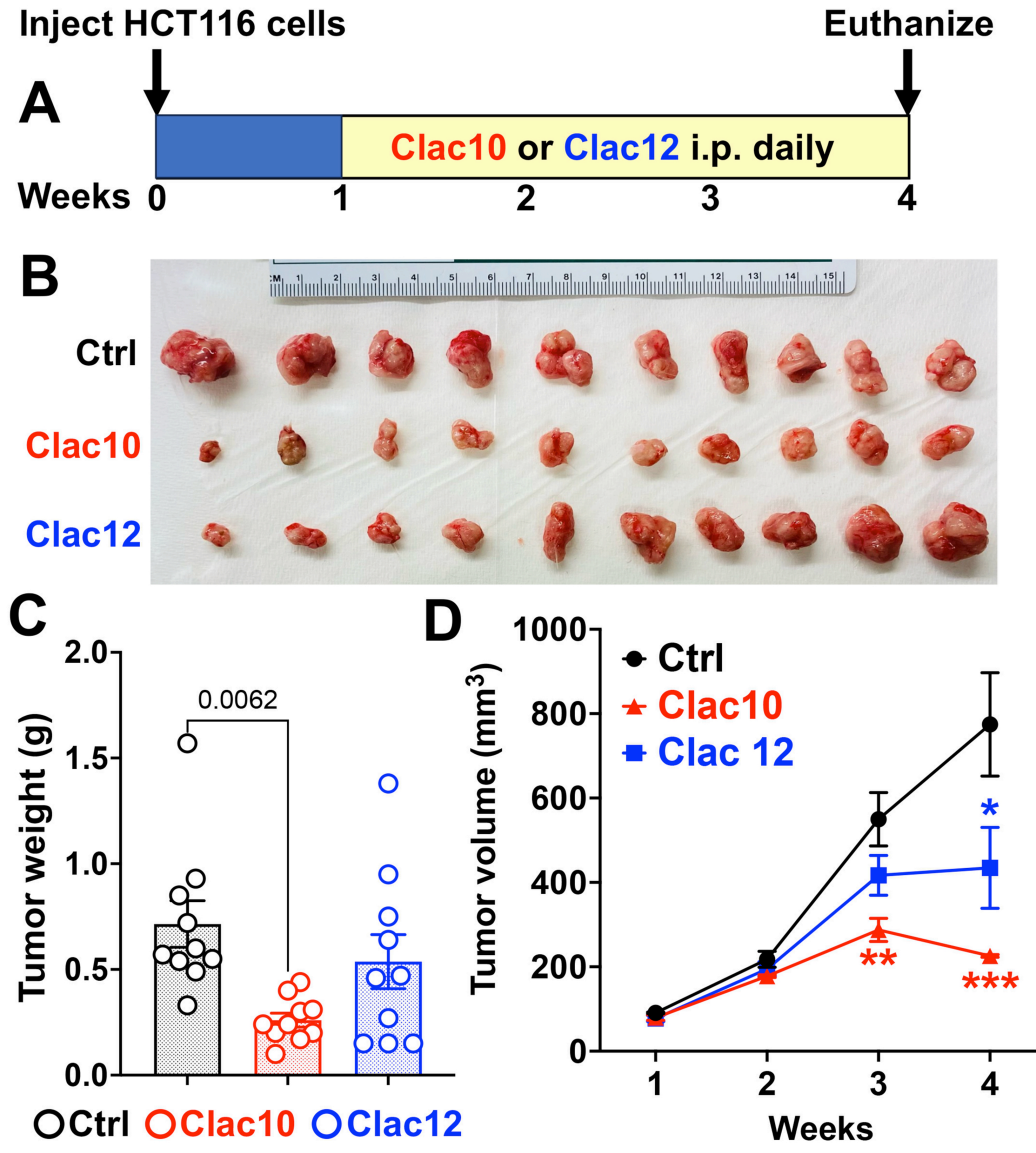
**Clac10** (**5c**) and **Clac12** (**5e**) reduce the growth of colon cancer xenografts in NSG mice. (A) HCT116 cells were injected into both flanks of NSG mice (*n* = 5), and tumors (*n* = 10; each mouse carrying 2 xenograft tumors, totaling 10 tumors per group) were allowed to develop until palpable. Then, **Clac10** and **Clac12** (20 mg/kg body weight) were injected intraperitoneally daily for 24 days; At the end of the study, the tumors were removed, imaged (B), and analyzed further; (C) The tumors in **Clac10**-treated mice were smaller than those in the control group; (D) Treatment with **Clac10** and **Clac12** resulted in significantly lower tumor volumes than in the control group. Tumor volume was measured weekly. The data are shown as mean ± SEM. We used ordinary one-way ANOVA for statistical comparisons. ^*^P < 0.05, ^**^P < 0.01, ^***^P < 0.001. SEM: Standard error of the mean; ANOVA: analysis of variance.

Compounds **5c** (**Clac10**) and **5e** (**Clac12**) were analyzed for their *in vivo* activity against HCT116 CRC xenografts in NSG mice [Figure 7A and B]. Mice were randomly assigned to control and treatment groups once the tumor volume reached approximately 90 mm^3^. Mice with HCT116 xenografts were treated with 20 mg/kg **5c** or **5e** (intraperitoneally, once daily) for 24 days, and the tumor volumes were measured weekly. At the end of the study, the mice were euthanized, and the tumors were removed, weighed, and subsequently analyzed. We found that compound **5e** treatment resulted in moderate tumor growth inhibition; treatment with **5c** significantly suppressed the xenograft tumor weight [Figure 7C] and volume [Figure 7D] compared to vehicle-treated controls. Moreover, we did not observe any gross changes in organ morphology or body weight, suggesting the compounds are nontoxic. These data indicate that compounds **5c** or **5e** could be potential therapeutic agents for treating CRC without causing toxicity.

**Figure 8:**
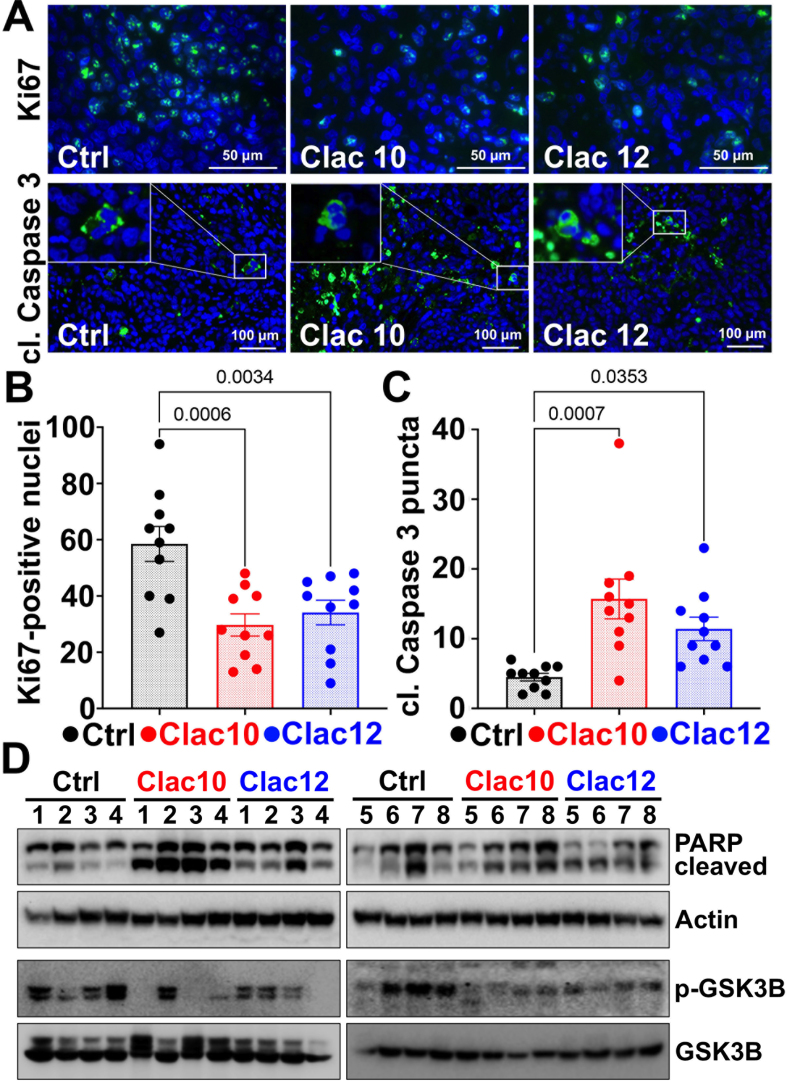
**Clac10** (**5c**) and **Clac12** (**5e**) suppress the growth of HCT116-tumor xenografts in mice. (A) Immunofluorescence reveals that compounds **5c** (**Clac10**) and **5e** (**Clac12**) reduce the number of proliferating cells (Ki67-positive nuclei) and increase the expression of cleaved caspase-3 in HCT116-xenograft tumors in mice; Quantification of Ki-67-positive cells (B) and cleaved caspase-3 staining (C) was performed; (D) Western blot analyses of tissue lysates from animals treated with **5c** (**Clac10**) and **5e** (**Clac12**) show significantly higher levels of cleaved PARP, indicating apoptosis, and reduced p-GSK3B expression, indicating inhibition of histamine-signaling. Data is presented as mean ± SEM. A statistical comparison was performed using an ordinary one-way ANOVA. PARP: Poly (ADP-ribose) polymerase; p-GSK3B: phosphorylated-glycogen synthase kinase 3 beta; SEM: standard error of the mean; ANOVA: analysis of variance.

Ki-67 is a proliferation marker protein^[68]^ and is widely used to assess the levels of proliferating cells in tumor xenograft models. Our immunohistochemical analysis (immunofluorescence) of tumors revealed a significant reduction in Ki-67 expression after treatment with compounds **5c** and **5e** [Figure 8A and B], suggesting antiproliferative effects *in vivo*. Our IHC analysis showed increased levels of the apoptotic marker protein cleaved caspase-3 in tumors treated with compounds **5c** and **5e** [Figure 8A and C]. Western blot analysis confirmed apoptosis by increased poly (ADP-ribose) polymerase (PARP) cleavage and showed inhibition of histamine signaling, as evidenced by decreased p-GSK3B levels in tumors treated with compounds **5c** and **5e** compared to untreated controls [Figure 8D and Supplementary Figure 11]. These datasets suggest that compounds **5c** and **5e** inhibited CRC tumor growth by inducing tumor cell apoptosis.

## DISCUSSION

Recent research has focused on targeting specific chemoresistance mechanisms to develop more effective treatments for CRC. Traditional strategies include inhibiting efflux pumps, such as P-gp, to enhance intracellular drug concentrations; modulating apoptotic pathways by targeting anti-apoptotic proteins, such as B-cell lymphoma 2 (Bcl-2) and Bcl-XL; and disrupting signaling pathways in CSCs, such as Wnt, Notch, and Hedgehog, to prevent tumor recurrence. Additionally, inhibiting DNA repair mechanisms with drugs like PARP inhibitors can impair cancer cells’ ability to repair chemotherapy-induced DNA damage. Combination therapies and adaptive strategies are often required, which complicate management and increase costs, necessitating ongoing research to improve treatment safety, specificity, and efficacy. Our study identified compounds **5c** and **5e** as promising derivatives that inhibit colon cancer cell proliferation in both p53 WT and KO cells, as well as in vinblastine-resistant KB-V1 cervix carcinoma cells overexpressing P-gp. This suggests their potential effectiveness against drug-resistant colon cancer, offering a valuable new approach to combating CRC chemoresistance. Notably, the anticancer activity of irinotecan was reduced by P-gp in KB-derived cells and thus the antiproliferative effects of **5c** and **5e** on KB-V1(Vbl) cells might also be of relevance for the treatment of irinotecan-resistant CRC^[69]^. Certain CRC cells showed irinotecan resistance based on P-gp expression and the combination with P-gp inhibitors sensitized the resistant CRC cells to irinotecan treatment^[70]^.

CSCs play a critical role in tumor survival, as they can self-renew and undergo asymmetric division, producing progenitor cells that differentiate into various cell types within the tumor^[71]^. CSC populations may also play a role in therapeutic resistance, thereby contributing to tumor recurrence and progression. Several surface markers have been identified for colorectal CSCs, such as CD44 and LGR5, *etc.*; however, nearly all of them appear to enrich for a subset of cells exhibiting stem cell-associated properties^[72]^. Therefore, targeting CSCs is a promising strategy for cancer treatment. Combination therapy with different chemotherapeutic agents has been shown to be effective in previous studies, especially in CRC^[73]^. The combination of our compounds **5c** and **5e** with 5-FU has shown significant synergy, indicating enhanced chemotherapy effectiveness. These findings imply that the combination of compounds **5c** and **5e** with 5-FU may represent a more efficacious therapeutic approach for CRC, warranting additional clinical investigation. Compounds **5c** and **5e** effectively target CSCs, which are vital for tumor growth and resistance, as evidenced by their ability to inhibit colony and spheroid formation. Further research is needed to understand how these compounds affect CSCs’ growth.

Causing cancer cells to undergo apoptosis has become a promising approach to treating the disease. Apoptosis can occur in response to stress, radiation, growth factor deficiency, or DNA damage caused by cytotoxic chemotherapeutic agents^[74]^. A decrease in Bcl-2 levels has been reported to induce apoptosis in CRC^[75]^. A reduction in the BCL-2 family of proteins may result in the loss of mitochondrial membrane potential (MMP) and the concomitant release of cytochrome c from mitochondria. This released cytochrome c subsequently induces an increase in caspase 3 activity and facilitates its dissociation PARP^[76]^. Our study observed an increase in caspase-3/7 levels following treatment with Compounds **5c** and **5e**, suggesting a caspase-mediated apoptotic mechanism. We observed not only a reduction in Bcl-2 after treatment with the 2-pyrazoline derivatives **5c** and **5e**, but also in other anti-apoptotic members of the Bcl-2 family, namely myeloid cell leukemia 1 (Mcl-1) and Bcl-XL. Given that these two proteins are recognized for their crucial roles in cancer cell survival and resistance to Bcl-2-based therapies, compounds **5c** and **5e** may be combined with standard-of-care treatment modalities to enhance therapeutic efficacy^[77]^. Furthermore, cyclins are crucial for cell transition between various phases of the cycle. Compounds **5c** and **5e** inhibited cyclin D1 expression, a critical protein overexpressed in CRC^[78]^, thereby promoting cell cycle progression and tumor growth^[79]^. 2-Pyrazoline derivatives **5c** and **5e** also modulate apoptotic pathways, overcoming apoptosis resistance by inducing cell cycle arrest and apoptosis.

Given these compounds’ effectiveness against both drug-resistant and sensitive cell lines, we explored non-traditional mechanistic targets behind these findings. We used molecular docking to study the binding of compounds **5c** and **5e** to HRH1. Both bind to the ECD of HRH1, thereby preventing histamine binding and inhibiting histamine-driven downstream signaling. We confirmed this binding using CETSA, which showed a stabilization in the HRH1 denaturation temperature upon compound binding. Furthermore, we observed that these compounds preferentially bind to HRH1 over HRH2. This is significant because HRH1 promotes tumor growth, whereas HRH2 inhibits tumor growth in CRC. Our study showed that compounds **5c** and **5e** bind to HRH1, which is upregulated in CRC, thereby disrupting histamine binding and downstream signaling pathways, including GSK3B and ERK. However, X-ray co-crystallographic data are needed to further confirm this binding. This interference with oncogenic signaling may restore chemosensitivity by suppressing pro-survival pathways. HRH1 overexpression has been demonstrated to be involved in tumor progression and is associated with poor prognosis in HCC^[7]^. More recently, histamine-HRH1 axes have been shown to confer resistance to anti-PD1 immunotherapy in melanoma^[8]^ patients, and blocking HRH1 has improved the effectiveness of anti-PD1 therapy in pancreatic cancer^[9]^. Based on these reports, we believe that HRH1 is partially involved in resistance to anti-PD1 therapy in cancer patients^[7-9]^. However, further research is necessary to elucidate its role in drug resistance mechanisms. Although some current antihistamine drugs have shown effectiveness in controlling the tumor growth, the long-term usage of first-generation antihistamines is associated with side effects such as drowsiness, cognitive effects, *etc.* While the second generation is better for long-term use, the dose-response studies for cancer treatment are not well established clinically^[10]^. Hence, HRH1 is a potential target for drug discovery to inhibit CRC tumor growth.

Tumor xenograft models have been successfully used in the past to test and develop anticancer drugs, including chemotherapeutic agents such as 5-FU^[80]^, and to assess dose calculations, survival rates, and tumor growth inhibition^[81]^. Considerable plasma stability and bioavailability were described for chloroacetamide-based drugs in mice, which support the significance and validity of our *in vivo* experiments with anticancer active *N*-chloroacetyl pyrazoline derivatives such as **5c** and **5e** in a suitable CRC model^[27]^. Treatment with compounds **5c** and **5e** suppressed the growth (tumor volume and weight) of HCT116 tumor xenografts in NSG mice by reducing Ki-67-positive cells and increasing PARP and cleaved-caspase 3 levels. Ki-67 is a proliferation marker protein^[68]^ and is widely used to assess the levels of proliferating cells in tumor xenograft models. Antiproliferative compounds have been shown to inhibit tumor growth by reducing the number of Ki67-positive cells. Caspase-3 cleaves PARP during apoptosis, rendering it inactive and thereby preventing DNA repair while promoting cell death^[82]^. The presence of cleaved caspase-3^[83]^ and PARP is often regarded as a hallmark of the execution phase of apoptosis, making them key indicators of apoptotic cells. These studies provide a rationale for targeting HRH1 and for further lead development of the pyrazoline class of compounds for the treatment of CRC, including their potential to effectively counteract drug-resistant CRC. For instance, optimizing the chloroacetamide side chain might be a useful strategy to improve the compounds’ anticancer and HRH1-binding properties. To prepare further promising derivatives, the presented chloroacetamide derivatives might already serve as suitable starting compounds for reactions with nucleophilic reagents (e.g., thiols, amines) and for the development of hybrid molecules^[13]^.

While our study offers promising insights into overcoming drug resistance in CRC, several limitations should be considered. Firstly, the *in vitro* findings, although significant, may not fully translate to clinical efficacy in human patients due to inherent differences between cell lines and complex tumor microenvironments. We primarily examined HRH1 binding, CSC inhibition, the apoptotic pathway, and interactions with efflux pumps. However, resistance mechanisms in CRC are complex and involve other pathways such as autophagy, metabolic changes, and microRNA regulation, which were not investigated here. Additionally, we did not assess the compounds’ long-term effectiveness or their potential to induce resistance, as cancer cells may adapt over time, necessitating combination therapies or further refinement of the compounds. Our study used specific CRC cell lines and NSG mice, which may not reflect the genetic diversity of the broader patient population. Future research using various CRC models, patient-derived xenografts, or spontaneous CRC models could yield more human CRC-relevant findings. Long-term studies and clinical trials will be essential to evaluate the safety, optimal dosing, pharmacokinetics, and efficacy of these compounds in a human population. Furthermore, understanding the detailed molecular mechanisms and interactions of these compounds is crucial, along with comprehensive toxicity and safety evaluations.

In conclusion, various new 2-pyrazoline derivatives with chloroacetamide appendages were successfully identified as potent anticancer agents. While this study primarily focused on anti-CRC effects, several compounds were also active against non-CRC tumors, indicating broad-spectrum anticancer activity independent of P-gp-mediated detoxification. The antiproliferative activity against CRC cells appeared to be p53-independent; thus, the 2-pyrazolines circumvent a possible resistance mechanism. Moreover, these compounds bind to the ECD of HRH1, preventing histamine binding and thereby inhibiting histamine-mediated downstream signaling and CRC tumor growth. Together with the observed pro-apoptotic properties, the described *in vivo* CRC growth inhibition by **5c,** which inhibits histamine-HRH1 axes, is noteworthy and supports the development of **5c** as a new anticancer drug targeting HRH1 in future studies.

## DECLARATIONS

### Availability of data and materials

The raw data supporting the findings of this study are available within this Article and its Supplementary Materials. Further data are available from the corresponding authors upon request.

### Copyright

© The Author(s) 2026.
