# Supplementary material for: Novel N-chloroacetyl-2-pyrazoline analogs with 2-naphthyl and ferrocenyl groups: targeting histamine receptor H1 to overcome colorectal cancer drug resistance
Source: Cancer Drug Resist. 2026 Apr 30;9:15. doi: 10.20517/cdr.2025.203 (PMC13174198; doi:10.20517/cdr.2025.203)
Supplement: Supplementary file 1 [file cdr-9-15-SupplementaryMaterials.pdf]

Cancer Drug Resistance

Open Access with Excellence

Supplementary Materials

Novel *N*-chloroacetyl-2-pyrazoline analogs with 2-naphthyl and ferrocenyl groups: targeting histamine receptor H1 to overcome colorectal cancer drug resistance

Dileepkumar Veeragoni[1,#], Hindole Ghosh[1,#], Affan Ansari[1], Sangita Bhattacharyya[1], Ahraar Azaz[1], Ojaswitha Ommi[1], Obdulia Covarrubias-Zambrano[1], Leonhard H. F. Köhler[2], Justus F. Ködel[2], Ameer Hamza[3], Anup Kasi[4], Stefan H. Bossmann[1], Rainer Schobert[2], Bernhard Biersack[2], Prasad Dandawate[1]

[1]Cancer Biology, University of Kansas Medical Center, Kansas City, KS 66160, USA.
[2]Organische Chemie I, Universität Bayreuth, Bayreuth 95447, Germany.
[3]Pathology and Laboratory Medicine, University of Kansas Medical Center, Kansas City, KS 66160, USA.
[4]Medical Oncology, University of Kansas Medical Center, Kansas City, KS 66160, USA.
[#]Authors contributed equally.

Correspondence to: Dr. Prasad Dandawate, Cancer Biology, University of Kansas Medical Center, Kansas City, KS 66160, USA. E-mail: pdandawate@kumc.edu; Dr. Bernhard Biersack, Organische Chemie 1, Universität Bayreuth, Bayreuth 95447, Germany. E-mail: bernhard.biersack@yahoo.com

Supplementary Methods

Synthesis of 2-pyrazolines

1-Chloroacetyl-5-(4-methoxyphenyl)-3-(ferrocenyl)-2-pyrazoline (4a) – Typical procedure

Chloroacetic acid (7 g) and hydrazine hydrate (0.6 mL) were added to 5-ferrocenyl-3-(4-methoxyphenyl)-prop-2-en-1-one (437 mg, 1.0 mmol). The reaction mixture was stirred under reflux for 2 h. The solution was poured on ice and the formed precipitate was dissolved in CH_2Cl_2, washed with water and dried over Na_2SO_4. The suspension was filtered and the filtrate was concentrated in vacuum. The residue was purified by column chromatography (silica gel 60, ethyl acetate / *n*-hexane 1:3). Yield: 85 mg (0.20 mmol, 20%); red-brown oil; υ_{max}(ATR)/cm^{-1} 3088, 2957, 2836, 1660, 1512, 1418, 1244, 1177, 1028, 821, 727; ^{1}H-NMR (300 MHz, $CDCl_3$): δ 2.95 (1 H, dd, J = 17.5 Hz, 4.6 Hz), 3.62 (1 H, dd, J = 17.5 Hz, 11.4 Hz), 3.76 (3 H, s), 4.1-4.2 (5 H, m), 4.4-4.5 (2 H, m), 4.49 (2 H, s), 4.5-4.6 (1 H, m), 4.6-4.7 (1 H, m), 5.48 (1 H, dd, J = 11.4 Hz, 4.6 Hz), 6.84 (2 H, d, J = 8.4 Hz), 7.16 (2 H, d, J = 8.4 Hz); ^{13}C NMR (75 MHz, $CDCl_3$): δ 42.2, 43.5, 55.2, 59.3, 67.5, 67.9, 69.5, 70.7, 74.3, 114.3, 126.7, 133.2, 157.7, 159.2, 163.2; HRMS for $C_{22}H_{22}ClFeN_2O_2$ [M$^+$ + H] calcd. 437.07137, found 437.07046.

1-Chloroacetyl-5-(3,4-dimethoxyphenyl)-3-(ferrocenyl)-2-pyrazoline (4b)

Analogously to the synthesis of **4a**, compound **4b** was obtained from chloroacetic acid (7 g), hydrazine hydrate (0.6 mL), and 5-ferrocenyl-3-(3,4-dimethoxyphenyl)-prop-2-en-1-one (376 mg, 1.0 mmol). Yield: 110 mg (0.24 mmol, 24%); yellow oil; υ_{max}(ATR)/cm^{-1} 3090, 2999, 2957, 2937, 2836, 1659, 1595, 1516, 1497, 1464, 1418, 1378, 1353, 1312, 1257, 1234, 1164, 1138, 1106, 1086, 1025, 1006, 911, 840, 819, 783, 763, 726; ^{1}H-NMR (300 MHz, $CDCl_3$): δ 2.96 (1 H, dd, J = 17.5 Hz, 4.6 Hz), 3.63 (1 H, dd, J = 17.5 Hz, 11.4 Hz), 3.82 (3 H, s), 3.85 (3 H, s), 4.1-4.2 (5 H, m), 4.4-4.5 (3 H, m), 4.5-4.6 (2 H, m), 4.6-4.7 (1 H, m), 5.49 (1 H, dd, J = 11.4 Hz, 4.6 Hz), 6.7-6.8 (3 H, m); ^{13}C NMR (75 MHz, $CDCl_3$): δ 42.1, 43.6, 55.9, 59.5, 67.4, 67.9, 69.5, 70.6, 70.7, 74.5, 108.9, 111.5, 117.4, 133.7, 148.6, 149.3, 157.7, 163.3; *m/z* (EI, %) 468 (35) [M$^+$], 466 (100) [M$^+$], 209 (22), 121 (36); Anal calcd for $C_{23}H_{23}ClFeN_2O_3$ (%): C, 59.19; H, 4.97. Found (%): C, 59.27; H, 5.02.

1-Chloroacetyl-5-(3,4,5-trimethoxyphenyl)-3-(ferrocenyl)-2-pyrazoline (4c)

Analogously to the synthesis of **4a**, compound **4c** was obtained from chloroacetic acid (7 g), hydrazine hydrate (0.6 mL), and 5-ferrocenyl-3-(3,4,5-trimethoxyphenyl)-prop-2-en-1-one (406 mg, 1.0 mmol). Yield: 70 mg (0.14 mmol, 14%); red-brown oil; υ_{max}(ATR)/cm^{-1} 3093, 2937, 2838, 1660, 1591, 1496, 1122, 1003, 820; ^{1}H-NMR (300 MHz, $CDCl_3$): δ 2.97 (1 H, dd, J = 4.6 Hz, 17.7 Hz), 3.03 (1 H, dd, J = 12 Hz, 17.7 Hz), 3.78 (3 H, s), 3.84 (6 H, s), 4.1-4.2 (6 H, m), 4.3-4.7 (5 H, m), 4.8-4.9 (2 H, m), 5.50 (1 H, d, J = 12.0 Hz, 4.6 Hz), 6.41 (2 H, s); ^{13}C NMR (75 MHz, $CDCl_3$): δ 42.0, 43.7, 56.1, 56.3, 59.8, 60.7, 67.5, 67.8, 68.3, 69.4, 70.7, 70.8, 74.3, 76.6, 77.0, 77.4, 102.1, 136.7, 137.5, 153.7, 158.8, 163.5; HRMS for $C_{24}H_{26}ClFeN_2O_4$ [M$^+$ + H] calcd. 497.09250, found 497.09206.

1-Chloroacetyl-5-(3-chloro-4,5-dimethoxyphenyl)-3-(ferrocenyl)-2-pyrazoline (4d)

Analogously to the synthesis of **4a**, compound **4d** was obtained from chloroacetic acid (7 g), hydrazine hydrate (0.6 mL), and 5-ferrocenyl-3-(3-chloro-4,5-dimethoxyphenyl)-prop-2-en-1-one (411 mg, 1.0 mmol). Yield: 85 mg (0.19 mmol, 19%); red-brown oil. υ_{max}(ATR)/cm^{-1} 3093, 3002, 2940, 2831, 1711, 1661, 1599, 1576, 1492, 1417, 1379, 1355, 1307, 1290, 1272, 1235, 1183, 1159, 1133, 1106, 1086, 1050, 1029, 1000, 907, 822, 785, 728, 687; ^{1}H NMR (300 MHz, $CDCl_3$) δ 2.9-3.0 (1 H, m), 3.6-3.7 (1 H, m), 3.81 (3 H, s), 3.84 (3 H, s), 4.1-4.2 (5 H, m), 4.4-4.7 (6 H, m), 5.42 (1 H, m), 6.70 (1 H, s), 6.80 (1 H, s); ^{13}C NMR (75.5 MHz, $CDCl_3$) δ 41.9, 43.5, 56.1, 59.2, 60.6, 67.5, 68.0, 69.5, 70.8, 70.9, 74.0, 108.5, 118.3, 128.9, 137.4, 144.9, 154.1, 158.4, 163.7; HRMS for $C_{23}H_{23}O_3N_2Cl_2Fe$ [M$^+$ + H] calcd. 501.04297, found 501.04258.

1-Chloroacetyl-5-(3-bromo-4,5-dimethoxyphenyl)-3-(ferrocenyl)-2-pyrazoline (4e)

Analogously to the synthesis of **4a**, compound **4e** was obtained from chloroacetic acid (7 g), hydrazine hydrate (0.6 mL), and 5-ferrocenyl-3-(3-bromo-4,4-dimethoxyphenyl)-prop-2-en-1-one (176 mg, 0.39 mmol). Yield: 30 mg (0.062 mmol, 16%); red-brown oil; υ_{max}(ATR)/cm^{-1} 3095, 3002, 2935, 2834, 1661, 1597, 1568, 1488, 1414, 1378, 1343, 1302, 1270, 1236, 1183, 1157, 1133, 1106, 1086, 1045, 999, 906, 820, 784, 768, 728, 686; ^{1}H NMR (300 MHz, $CDCl_3$) δ 3.00 (1 H, dd, J = 17.6 Hz, 4.1 Hz), 3.63 (1 H, dd, J = 17.6 Hz, 11.5 Hz), 3.80 (3 H, s), 3.84 (3 H, s), 4.1-4.2 (5H, m), 4.4-4.7 (6 H, m), 5.43 (1 H, dd, J = 11.5 Hz, 4.1 Hz), 6.75 (1 H, s), 6.97 (1 H, s); ^{13}C NMR (75.5 MHz, $CDCl_3$) δ 42.1, 43.6, 56.1, 59.1, 60.6,

67.5, 68.0, 69.6, 70.8, 71.0, 74.1, 109.3, 118.3, 121.2, 138.2, 146.1, 154.0, 158.1, 163.6; HRMS for $C_{23}H_{23}O_3N_2BrClFe$ [M^+ + H] calcd. 544.99245 and 546.99040, found 544.99222 and 546.99000.

1-Chloroacetyl-5-(3,4-dimethoxy-5-iodophenyl)-3-(ferrocenyl)-2-pyrazoline (4f)

Analogously to the synthesis of **4a**, compound **4f** was obtained from chloroacetic acid (7 g), hydrazine hydrate (0.6 mL), and 5-ferrocenyl-3-(3,4-dimethoxy-5-iodophenyl)-prop-2-en-1-one (232 mg, 0.46 mmol). Yield: 40 mg (0.075 mmol, 16%); red-brown oil; υ_{max}(ATR)/cm^{-1} 3097, 2999, 2967, 2937, 2831, 1736, 1660, 1593, 1562, 1497, 1481, 1412, 1379, 1316, 1297, 1267, 1236, 1182, 1134, 1106, 1086, 1042, 999, 906, 822, 785, 764, 729, 686; ^{1}H NMR (300 MHz, $CDCl_3$) δ 3.00 (1 H, dd, J = 17.6 Hz, 4.3 Hz), 3.62 (1 H, dd, J = 17.6 Hz, 11.5 Hz), 3.78 (3 H, s), 3.83 (3 H, s), 4.1-4.2 (5H, m), 4.4-4.7 (6 H, m), 5.42 (1 H, dd, J = 11.5 Hz, 4.3 Hz), 6.77 (1 H, s), 7.17 (1 H, s); ^{13}C NMR (75.5 MHz, $CDCl_3$) δ 42.0, 43.5, 56.0, 58.8, 60.4, 67.5, 68.0, 69.5, 70.8, 71.0, 74.1, 93.1, 110.5, 126.8, 138.9, 148.5, 152.8, 158.3, 163.7; HRMS for $C_{23}H_{23}O_3N_2ClFeI$ [M^+ + H] calcd. 592.97858, found 592.97733.

1-Chloroacetyl-5-(3-bromophenyl)-3-(ferrocenyl)-2-pyrazoline (4g)

Analogously to the synthesis of **4a**, compound **4g** was obtained from chloroacetic acid (7 g), hydrazine hydrate (0.6 mL), and 5-ferrocenyl-3-(3-bromophenyl)-prop-2-en-1-one (397 mg, 1.0 mmol). Yield: 66 mg (0.14 mmol, 14%); red-brown oil; υ_{max}(ATR)/cm^{-1} 3091, 2955, 1742, 1665, 1596, 1570, 1496, 1476, 1421, 1378, 1316, 1246, 1197, 1166, 1132, 1106, 1086, 1072, 1027, 998, 961, 911, 866, 821, 783, 728, 692, 667; ^{1}H NMR (300 MHz, $CDCl_3$) δ 2.9-3.0 (1 H, m), 3.6-3.7 (1 H, m), 4.1-4.2 (5H, m), 4.4-4.5 (2 H, m), 4.5-4.6 (2 H, m), 4.67 (1 H, s), 7.2-7.3 (2 H, m), 7.3-7.4 (2 H, m); ^{13}C NMR (75.5 MHz, $CDCl_3$) δ 42.0, 43.5, 59.2, 67.5, 68.0, 69.5, 70.8, 71.0, 74.1, 123.1, 124.2, 128.4, 130.6, 131.1, 143.1, 158.4, 163.7; HRMS for $C_{21}H_{19}ON_2BrClFe$ [M^+ + H] calcd. 484.97132 and 486.96928, found 484.97057 and 486.96805.

1-Chloroacetyl-5-(3,4-methylenedioxyphenyl)-3-(ferrocenyl)-2-pyrazoline (4h)

Analogously to the synthesis of **4a**, compound **4h** was obtained from chloroacetic acid (7 g), hydrazine hydrate (0.6 mL), and 5-ferrocenyl-3-(3,4-methylenedioxyphenyl)-prop-2-en-1-one (394 mg, 1.0 mmol). Yield: 98 mg (0.22 mmol, 22%), red-brown solid; υ_{max}(ATR)/cm^{-1} 3081, 2887, 1735, 1663, 1487, 1243, 1032, 818, 710, 632; ^{1}H-NMR (300 MHz, $CDCl_3$): δ 2.98 (1 H, dd, J

= 4.6 Hz, 17.5 Hz), 3.62 (1 H, dd, J = 17.5 Hz, 11.4 Hz), 4.1-4.2 (5 H, m), 4.4-4.5 (2 H, m), 4.50 (2 H, s), 4.5-4.6 (1 H, m), 4.6-4.7 (1 H, m), 5.44 (1 H, dd, J = 11.4 Hz, 4.6 Hz), 5.91 (2 H, s), 6.7-6.8 (3 H, m); ^{13}C NMR (75 MHz, $CDCl_3$): δ 42.0, 43.6, 59.5, 66.5, 68.3, 69.5, 70.6, 70.8, 74.5, 77.0, 101.2, 105.8, 108.5, 118.8, 135.0, 148.2, 157.7, 163.3; HRMS for $C_{22}H_{20}ClFeN_2O_3$ [M^+ + H] calcd. 451.05064, found 451.04945.

1-Chloroacetyl-3-(naphth-2-yl)-5-(4-methoxyphenyl)-2-pyrazoline (5a) – Typical procedure

Chloroacetic acid (8 g) and hydrazine hydrate (0.5 mL) were added to 5-(naphth-2-yl)-3-(4-methoxyphenyl)-prop-2-en-1-one (200 mg, 0.67 mmol). The reaction mixture was stirred under reflux for 3 h. The solution was poured on ice and the formed precipitate was collected and purified by column chromatography (silica gel 60, ethyl acetate / *n*-hexane 1:1). Yield: 106 mg (0.28 mmol, 42%); colorless gum; υ_{max}(ATR)/cm^{-1} 3003, 2964, 2924, 2833, 1735, 1685, 1612, 1514, 1480, 1419, 1402, 1368, 1329, 1291, 1243, 1179, 1135, 1110, 1035, 986, 949, 921, 884, 858, 825, 816, 803, 782, 752, 722, 704; ^{1}H NMR (300 MHz, $CDCl_3$) δ 3.32 (1 H, dd, J = 17.8 Hz, 4.6 Hz), 3.75 (3 H, s), 3.82 (1 H, dd, J = 17.8 Hz, 11.6 Hz), 4.61 (2 H, s), 5.57 (1 H, dd, J = 11.6 Hz, 4.6 Hz), 6.84 (2 H, d, J = 8.8 Hz), 7.19 (2 H, d, J = 8.8 Hz), 7.5-7.6 (2 H, m), 7.8-7.9 (3 H, m), 7.95 (1 H, s), 8.03 (1 H, d, J = 8.6 Hz); ^{13}C NMR (75.5 MHz, $CDCl_3$) δ 42.2, 55.2, 60.1, 114.3, 123.2, 126.9, 127.1, 127.5, 127.9, 128.3, 128.4, 128.6, 132.9, 134.3, 155.7, 159.2, 164.1; HRMS for $C_{22}H_{20}O_2N_2Cl$ [M^+ + H] calcd. 379.12078, found 379.12036.

1-Chloroacetyl-5-(3,4-dimethoxyphenyl)-3-(naphthalen-2-yl)-2-pyrazoline (5b)

Analogously to the synthesis of **5a**, compound **5b** was obtained from chloroacetic acid (8 g), hydrazine hydrate (0.5 mL), and 5-(naphthalene-2-yl)-3-(3,4-dimethoxyphenyl)-prop-2-en-1-one (529 mg, 1.294 mmol). Yield: 161 mg (0.39 mmol, 30%); yellow oil; υ_{max}(ATR)/cm^{-1} 3062, 2999, 2957, 2957, 2936, 2835, 1667, 1594, 1516, 1479, 1419, 1361, 1331, 1311, 1256, 1235, 1194, 1139, 1024, 896, 859, 819, 783, 750, 709; ^{1}H NMR (300 MHz, $CDCl_3$) δ 3.36 (1 H, dd, J = 17.8 Hz, 4.7 Hz), 3.8-3.9 (7 H, m), 4.0-4.1 (1 H, m), 4.5-4.7 (2 H, m), 5.58 (1 H, dd, J = 11.6 Hz, 4.7 Hz), 6.7-6.8 (3 H, m), 7.5-7.6 (2 H, m), 7.8-7.9 (3 H, m), 7.96 (1 H, s), 8.04 (1 H, d, J = 8.6 Hz); ^{13}C NMR (75.5 MHz, $CDCl_3$) δ 42.2, 56.0, 60.4, 109.2, 111.6, 117.9, 123.2, 126.9, 127.6, 127.6, 127.9, 128.4, 128.5, 128.7, 132.9, 133.5, 134.4, 148.8, 149.3, 155.6, 164.1; *m/z* (EI, %) 408 (93) [M^+], 331 (43), 255 (34), 220 (44), 206

(100), 164 (23); Anal calcd for $C_{23}H_{21}ClN_2O_3$ (%): C, 67.56; H, 5.18. Found (%): C, 67.66; H, 5.26.

1-Chloroacetyl-3-(naphth-2-yl)-5-(3,4,5-trimethoxyphenyl)-2-pyrazoline (5c)

Analogously to the synthesis of **5a**, compound **5c** was obtained from chloroacetic acid (10 g), hydrazine hydrate (1 mL), and 5-(naphth-2-yl)-3-(3,4,5-trimethoxyphenyl)-prop-2-en-1-one (500 mg, 1.44 mmol). Yield: 350 mg (0.80 mmol, 56%); off-white solid of mp 163-164°C; υ_{max}(ATR)/cm^{-1} 3042, 2944, 2836, 1681, 1592, 1508, 1481, 1463, 1449, 1427, 1419, 1394, 1365, 1351, 1328, 1292, 1241, 1189, 1159, 1122, 1042, 1004, 962, 928, 914, 892, 865, 855, 845, 832, 820, 773, 750, 703, 691, 657; ^{1}H NMR (300 MHz, $CDCl_3$) δ 3.33 (1 H, dd, J = 17.7 Hz, 4.8 Hz), 3.7-3.9 (10 H, m), 4.4-4.8 (2 H, m), 5.54 (1 H, dd, J = 11.7 Hz, 4.8 Hz), 7.5-7.6 (2 H, m), 7.8-7.9 (3 H, m), 7.94 (1 H, s), 8.03 (1 H, dd, J = 8.6 Hz, 1.7 Hz); ^{13}C NMR (75.5 MHz, $CDCl_3$) δ 42.0, 42.4, 56.1, 60.6, 60.7, 102.4, 123.2, 126.9, 127.5, 127.6, 127.8, 128.2, 128.4, 128.6, 132.8, 134.3, 136.5, 137.5, 153.7, 155.5, 164.1; HRMS for $C_{24}H_{24}O_4N_2$ [M^+ + H] calcd. 439.14191, found 439.14052.

1-Chloroacetyl-3-(naphth-2-yl)-5-(3-chloro-4,5-dimethoxyphenyl)-2-pyrazoline (5d)

Analogously to the synthesis of **5a**, compound **5d** was obtained from chloroacetic acid (8 g), hydrazine hydrate (0.5 mL), and 5-(naphth-2-yl)-3-(3-chloro-4,5-dimethoxyphenyl)-prop-2-en-1-one (200 mg, 0.49 mmol). Yield: 130 mg (0.29 mmol, 59%); colorless solid of mp 86-88°C; υ_{max}(ATR)/cm^{-1} 3057, 3004, 2938, 2829, 1667, 1600, 1575, 1480, 1418, 1362, 1331, 1307, 1272, 1234, 1135, 1050, 999, 911, 858, 819, 784, 770, 749, 714, 678, 652; ^{1}H NMR (300 MHz, $CDCl_3$) δ 3.34 (1 H, dd, J = 17.8 Hz, 4.9 Hz), 3.81 (3 H, s), 3.83 (3 H, s), 3.9-4.0 (1 H, m), 4.5-4.7 (2 H, m), 5.53 (1 H, dd, J = 11.7 Hz, 4.9 Hz), 6.73 (1 H, s), 6.83 (1 H, s), 7.5-7.6 (2 H, m), 7.8-7.9 (3 H, m), 7.95 (1 H, s), 8.03 (1 H, dd, J = 8.6 Hz, 1.7 Hz); ^{13}C NMR (75.5 MHz, $CDCl_3$) δ 42.1, 42.2, 56.2, 60.1, 60.6, 108.8, 118.7, 123.2, 127.0, 127.7, 127.9, 128.1, 128.5, 128.8, 128.9, 132.9, 134.4, 137.3, 145.1, 154.1, 155.5, 164.2; HRMS for $C_{23}H_{21}O_3N_2Cl_2$ [M^+ + H] calcd. 443.09237, found 443.09155.

1-Chloroacetyl-3-(naphth-2-yl)-5-(3-bromo-4,5-dimethoxyphenyl)-2-pyrazoline (5e)

Analogously to the synthesis of **5a**, compound **5e** was obtained from chloroacetic acid (8 g), hydrazine hydrate (0.5 mL), and 5-(naphth-2-yl)-3-(3-bromo-4,5-dimethoxyphenyl)-prop-2-en-1-one (200 mg, 0.44

mmol). Yield: 100 mg (0.21 mmol, 48%); colorless solid of mp 95-97°C; υ_{max}(ATR)/cm^{-1} 3051, 3002, 2935, 2876, 2831, 1667, 1598, 1568, 1480, 1427, 1362, 1331, 1302, 1271, 1249, 1232, 1183, 1135, 1046, 999, 910, 859, 843, 819, 784, 767, 749, 714; ^{1}H NMR (300 MHz, CDCl$_3$) δ 3.32 (1 H, dd, J = 17.8 Hz, 4.9 Hz), 3.80 (3 H, s), 3.8-3.9 (4 H, m), 4.6-4.7 (2 H, m), 5.5-5.6 (1 H, m), 6.76 (1 H, s), 6.99 (1 H, s), 7.5-7.6 (2 H, m), 7.8-7.9 (3 H, m), 7.94 (1 H, s), 8.03 (1 H, dd, J = 8.6 Hz, 1.7 Hz); ^{13}C NMR (75.5 MHz, CDCl$_3$) δ 42.1, 42.2, 56.1, 59.9, 60.5, 109.5, 118.2, 121.4, 123.5, 126.9, 127.6, 127.7, 127.9, 128.1, 128.5, 128.7, 132.9, 134.4, 137.9, 146.0, 153.9, 155.5, 164.1; HRMS for $C_{23}H_{21}O_3N_2{}^{81}BrCl$ [M$^+$ + H] calcd. 489.03981, found 489.03843.

1-Chloroacetyl-3-(naphth-2-yl)-5-(3-iodo-4,5-dimethoxyphenyl)-2-pyrazoline (5f)

Analogously to the synthesis of **5a**, compound **5f** was obtained from chloroacetic acid (8 g), hydrazine hydrate (0.5 mL), and 5-(naphth-2-yl)-3-(3-iodo-4,5-dimethoxyphenyl)-prop-2-en-1-one (200 mg, 0.40 mmol). Yield: 112 mg (0.21 mmol, 53%); colorless solid of mp 89-90°C; υ_{max}(ATR)/cm^{-1} 3055, 3005, 2959, 2935, 2828, 1667, 1591, 1563, 1479, 1426, 1362, 1330, 1297, 1269, 1249, 1232, 1182, 1135, 1043, 998, 910, 858, 844, 818, 784, 749, 733, 715, 685, 647; ^{1}H NMR (300 MHz, CDCl$_3$) δ 3.30 (1 H, dd, J = 17.8 Hz, 5.0 Hz), 3.78 (3 H, s), 3.8-3.9 (4 H, m), 4.5-4.7 (2 H, m), 5.4-5.5 (1 H, m), 6.78 (1 H, s), 7.19 (1 H, s), 7.5-7.6 (2 H, m), 7.8-7.9 (3 H, m), 7.92 (1 H, s), 8.02 (1 H, dd, J = 8.6 Hz, 1.7 Hz); ^{13}C NMR (75.5 MHz, CDCl$_3$) δ 42.1, 55.9, 59.6, 60.3, 93.0, 110.5, 123.1, 126.9, 127.1, 127.6, 127.7, 127.8, 128.0, 128.4, 128.6, 132.8, 134.3, 138.7, 148.5, 152.8, 155.4, 164.1; HRMS for $C_{23}H_{21}O_3N_2ClI$ [M$^+$ + H] calcd. 535.02799, found 535.02701.

1-Chloroacetyl-3-(naphth-2-yl)-5-(3-bromophenyl)-2-pyrazoline (5g)

Analogously to the synthesis of **5a**, compound **5g** was obtained from chloroacetic acid (8 g), hydrazine hydrate (0.5 mL), and 5-(naphth-2-yl)-3-(3-bromophenyl)-prop-2-en-1-one (337 mg, 1.0 mmol). Yield: 170 mg (0.40 mmol, 40%); colorless gum; υ_{max}(ATR)/cm^{-1} 3054, 1677, 1596, 1570, 1480, 1429, 1407, 1364, 1331, 1273, 1252, 1227, 1201, 1141, 1092, 1069, 1035, 998, 955, 911, 891, 863, 835, 826, 782, 754, 732, 707, 691, 677; ^{1}H NMR (300 MHz, CDCl$_3$) δ 3.30 (1 H, dd, J = 17.8 Hz, 4.9 Hz), 3.86 (1 H, dd, J = 17.8 Hz, 11.8 Hz), 4.6-4.7 (2 H, m), 5.55 (1 H, dd, J = 11.8 Hz, 4.9 Hz), 7.1-7.2 (2 H, m), 7.3-7.4 (2 H, m), 7.5-7.6 (2 H, m), 7.8-7.9 (3 H, m), 8.01 (1 H, d, J = 8.6 Hz); ^{13}C NMR (75.5 MHz, CDCl$_3$) δ 42.3,

60.0, 123.2, 124.0, 126.9, 127.6, 127.9, 128.5, 128.8, 129.8, 130.6, 131.1, 132.9, 134.4, 143.0, 155.4, 164.1; Anal calcd for $C_{21}H_{16}BrClN_2O$ (%): C, 58.97; H, 3.77. Found (%): C, 59.10; H, 3.82.

Synthesis of new ferrocenyl chalcone precursors

1-Ferrocenyl-3-(3-chloro-4,5-dimethoxyphenyl)-prop-2-en-1-one 2d

Acetylferrocene (1.023 g, 4.49 mmol) and 3-chloro-4,5-dimethoxybenzaldehyde (900 mg, 4.49 mmol) were dissolved in EtOH (25 mL) and aqueous NaOH (40%, 10 mL) was added. The reaction mixture was stirred at room temperature for 24 h. The formed precipitate was collected, washed with EtOH and dried. Yield: 1.16 g (2.83 mmol, 63%); red-brown solid, m.p. = 172-173 °C; υ_{max}(ATR)/cm^{-1} 3076, 2964, 2936, 2834, 1651, 1591, 1562, 1492, 1453, 1412, 1374, 1353, 1339, 1274, 1247, 1231, 1192, 1153, 1107, 1077, 1052, 1028, 988, 912, 848, 836, 821, 730; ^{1}H NMR (300 MHz, $CDCl_3$) δ 3.90 (3 H, s), 3.92 (3 H, s), 4.1-4.2 (5H, m), 4.5-4.6 (2 H, m), 4.8-4.9 (2 H, m), 6.9-7.0 (2 H, m), 7.31 (1, H, s), 7.63 (1 H, d, J = 15.6 Hz); ^{13}C NMR (75.5 MHz, $CDCl_3$) δ 56.2, 60.9, 69.7, 70.1, 72.9, 80.4, 111.4, 121.2, 123.3, 128.9, 131.7, 139.3, 146.9, 154.0, 192.5; HRMS for $C_{21}H_{20}O_3ClFe$ [M^+ + H] calcd. 411.04449, found 411.04347.

1-Ferrocenyl-3-(3-bromo-4,5-dimethoxyphenyl)-prop-2-en-1-one 2e

Acetylferrocene (372 mg, 1.63 mmol) and 3-bromo-4,5-dimethoxybenzaldehyde (400 mg, 1.63 mmol) were dissolved in EtOH (25 mL) and aqueous NaOH (40%, 10 mL) was added. The reaction mixture was stirred at room temperature for 24 h. The formed precipitate was collected, washed with EtOH and dried. Yield: 728 mg (1.16 mmol, 71%); red-brown solid of m.p. 167 °C; υ_{max}(ATR)/cm^{-1} 3073, 2936, 2834, 1649, 1588, 1554, 1488, 1451, 1432, 1410, 1374, 1353, 1333, 1269, 1229, 1192, 1180, 1151, 1106, 1075, 1047, 1028, 985, 912, 835, 820, 765, 728; ^{1}H NMR (300 MHz, $CDCl_3$) δ 3.89 (3 H, s), 3.92 (3 H, s), 4.1-4.2 (5H, m), 4.5-4.6 (2 H, m), 4.8-4.9 (2 H, m), 6.98 (1 H, d, J = 15.6 Hz), 7.04 (1 H, s), 7.47 (1 H, s), 7.63 (1 H, d, J = 15.6 Hz), 7.77 (1 H, s); ^{13}C NMR (75.5 MHz, $CDCl_3$) δ 56.2, 60.8, 69.8, 70.1, 72.9, 80.4, 112.1, 118.2, 123.3, 124.0, 132.3, 139.2, 147.9, 153.8, 192.5; HRMS for $C_{21}H_{20}O_3BrFe$ [M^+ + H] calcd. 454.99398 and 456.99193, found 454.99226 and 456.98999.

1-Ferrocenyl-3-(3-iodo-4,5-dimethoxyphenyl)-prop-2-en-1-one 2f

Acetylferrocene (629 mg, 2.76 mmol) and 3,4-dimethoxy-5-iodobenzaldehyde (805 mg, 2.76 mmol) were dissolved in EtOH (25 mL) and aqueous NaOH (40%, 10 mL) was

added. The reaction mixture was stirred at room temperature for 24 h. The formed precipitate was collected, washed with EtOH and dried. Yield: 929 mg (1.85 mmol, 67%); red-brown solid, m.p. 159°C; υ_{max}(ATR)/cm^{-1} 3097, 2929, 2869, 2823, 1648, 1586, 1549, 1480, 1452, 1426, 1407, 1375, 1353, 1329, 1299, 1266, 1228, 1194, 1138, 1106, 1079, 1044, 1028, 997, 972, 912, 881, 835, 818, 793, 763, 727; ^{1}H NMR (300 MHz, $CDCl_3$) δ 3.87 (3 H, s), 3.91 (3 H, s), 4.1-4.2 (5H, m), 4.5-4.6 (2 H, m), 4.8-4.9 (2 H, m), 6.97 (1 H, d, J = 15.6 Hz), 7.06 (1 H, s),7.62 (1 H, d, J = 15.6 Hz), 7.67 (1 H, s); ^{13}C NMR (75.5 MHz, $CDCl_3$) δ 56.1, 60.6, 69.8, 70.1, 72.9, 80.4, 93.0, 113.2, 123.2, 129.9, 133.2, 139.0, 150.5, 152.6, 192.5; HRMS for $C_{21}H_{20}O_3FeI$ [M^+ + H] calcd. 502.98011, found 502.97791.

1-Ferrocenyl-3-(3-bromophenyl)-prop-2-en-1-one 2g

Acetylferrocene (629 mg, 2.76 mmol) and 3-bromobenzaldehyde (511 mg, 2.76 mmol) were dissolved in EtOH (25 mL) and aqueous NaOH (40%, 10 mL) was added. The reaction mixture was stirred at room temperature for 24 h. The formed precipitate was collected, washed with EtOH and dried. Yield: 842 mg (2.13 mmol, 77%); red-brown solid, m.p. 159 °C; υ_{max}(ATR)/cm^{-1} 3118, 3090, 1654, 1601, 1560, 1480, 1455, 1409, 1377, 1340, 1323, 1308, 1242, 1200, 1105, 1084, 1072, 1030, 995, 984, 917, 900, 880, 864, 838, 827, 791, 783, 732, 677, 669; ^{1}H NMR (300 MHz, $CDCl_3$) δ 4.1-4.2 (5H, m), 4.5-4.6 (2 H, m), 4.8-4.9 (2 H, m), 7.07 (1 H, d, J = 15.6 Hz), 7.2-7.3 (1 H, m), 7.5-7.6 (2 H, m), 7.68 (1 H, d, J = 15.6 Hz), 7.77 (1 H, s); ^{13}C NMR (75.5 MHz, $CDCl_3$) δ 69.8, 70.1, 73.0, 80.4, 123.1, 124.2, 127.2, 130.5, 132.8, 137.4, 139.1, 192.5; HRMS for $C_{19}H_{16}OBrFe$ [M^+ + H] calcd. 394.97285 and 396.97080, found 394.97127 and 396.96894.

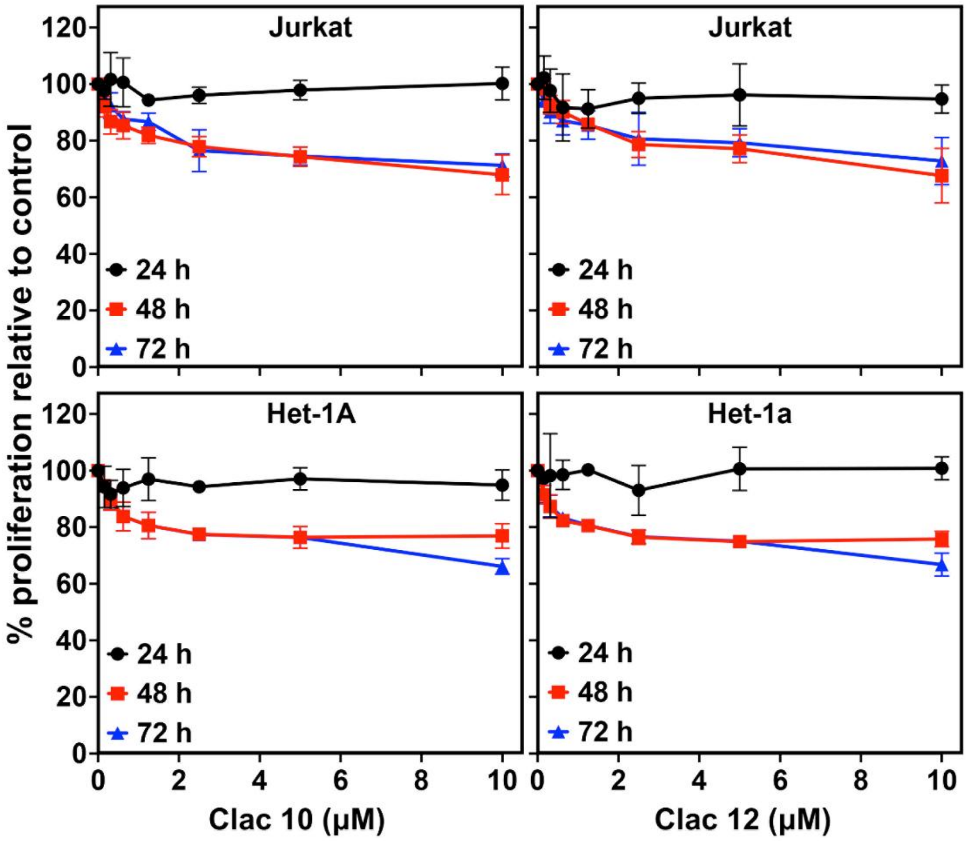

Supplementary Figure 1. Compounds **5c** (**Clac10**) and **5e** (**Clac12**) did not inhibit the growth of non-cancerous Jurkat and Het-1A cell lines. (A) Cells treated with 0-10 μM of each compound for up to 72 hours did not inhibit the growth of non-cancerous Jurkat (a human T lymphocyte cell) and Het-1A (an immortalized human esophageal epithelial cell line) cell lines ($IC_{50} > 10$ μM), indicating selective cytotoxicity towards tumor cells.

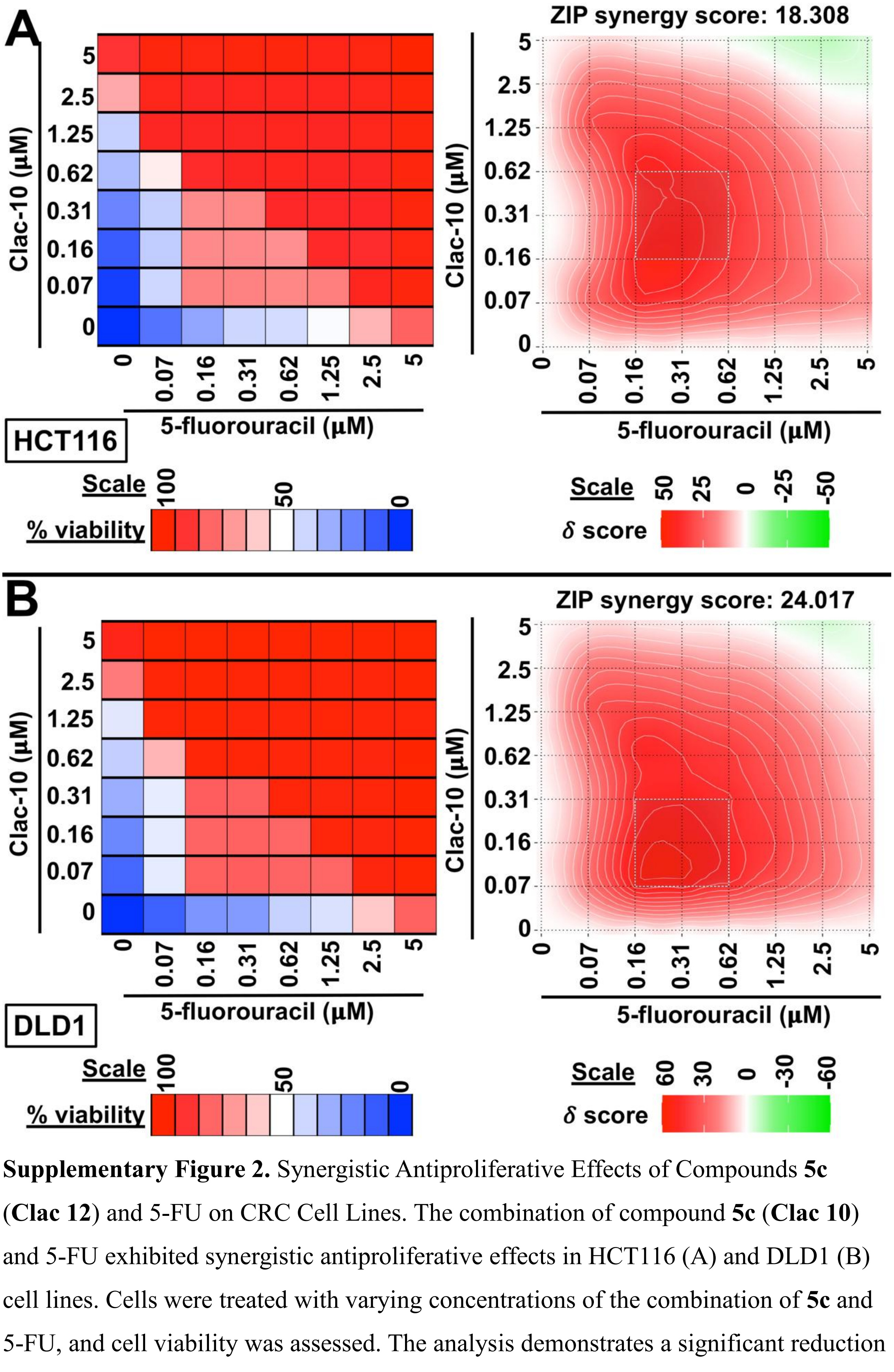

Supplementary Figure 2. Synergistic Antiproliferative Effects of Compounds **5c** (**Clac 12**) and 5-FU on CRC Cell Lines. The combination of compound **5c** (**Clac 10**) and 5-FU exhibited synergistic antiproliferative effects in HCT116 (A) and DLD1 (B) cell lines. Cells were treated with varying concentrations of the combination of **5c** and 5-FU, and cell viability was assessed. The analysis demonstrates a significant reduction in cell proliferation when both agents are used in combination compared to their individual effects. Synergistic effects were determined using Synergy Finder.

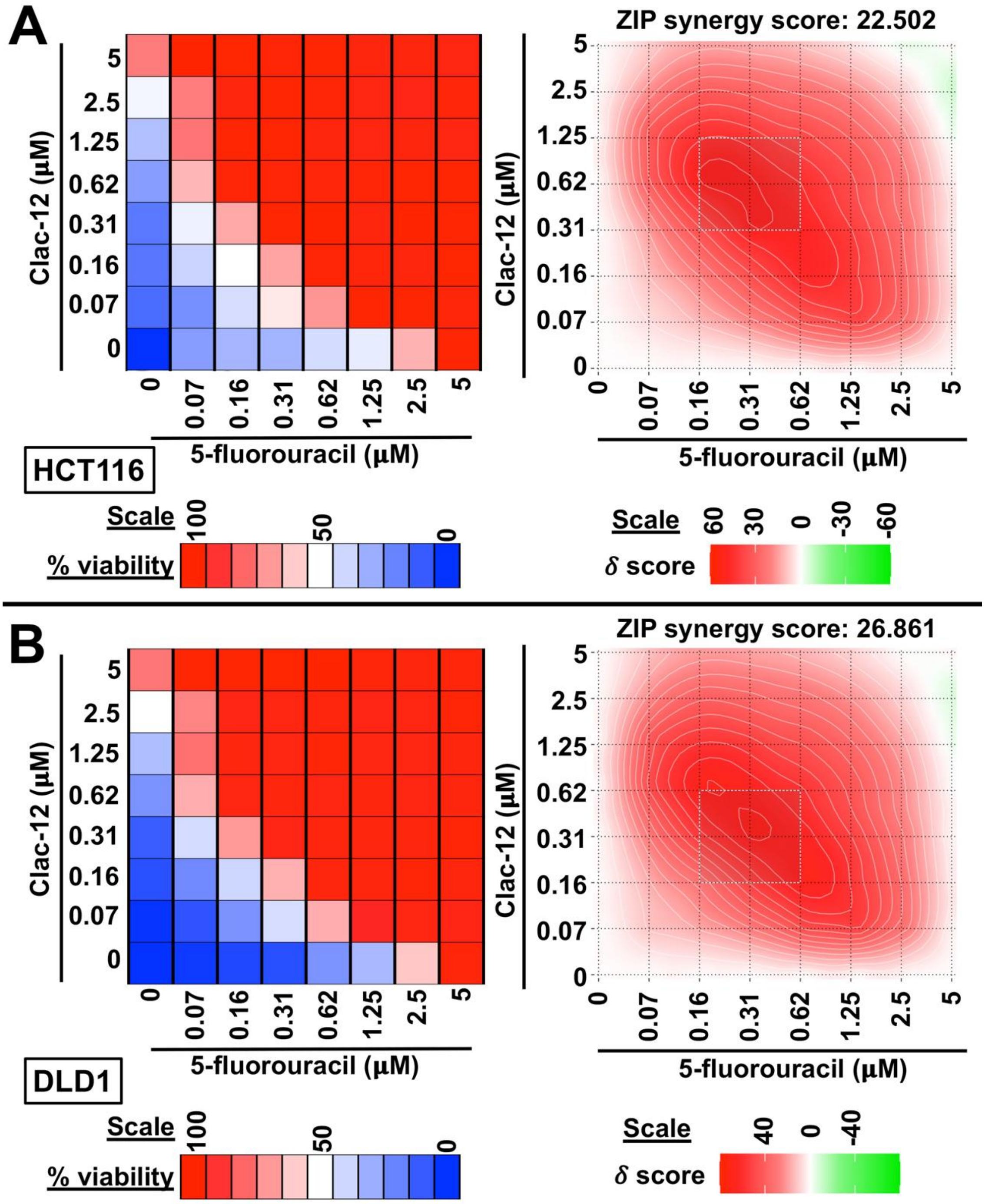

Supplementary Figure 3. Synergistic Antiproliferative Effects of Compounds **5e** (**Clac 12**) and 5-FU on CRC Cell Lines. The combination of compound **5e** (**Clac 12**) and 5-FU exhibited synergistic antiproliferative effects in HCT116 (A) and DLD1 (B) cell lines. Cells were treated with varying concentrations of the combination of **5e** and 5-FU, and cell viability was assessed. The analysis demonstrates a significant reduction in cell proliferation when both agents are used in combination compared to their individual effects. Synergistic effects were determined using Synergy Finder.

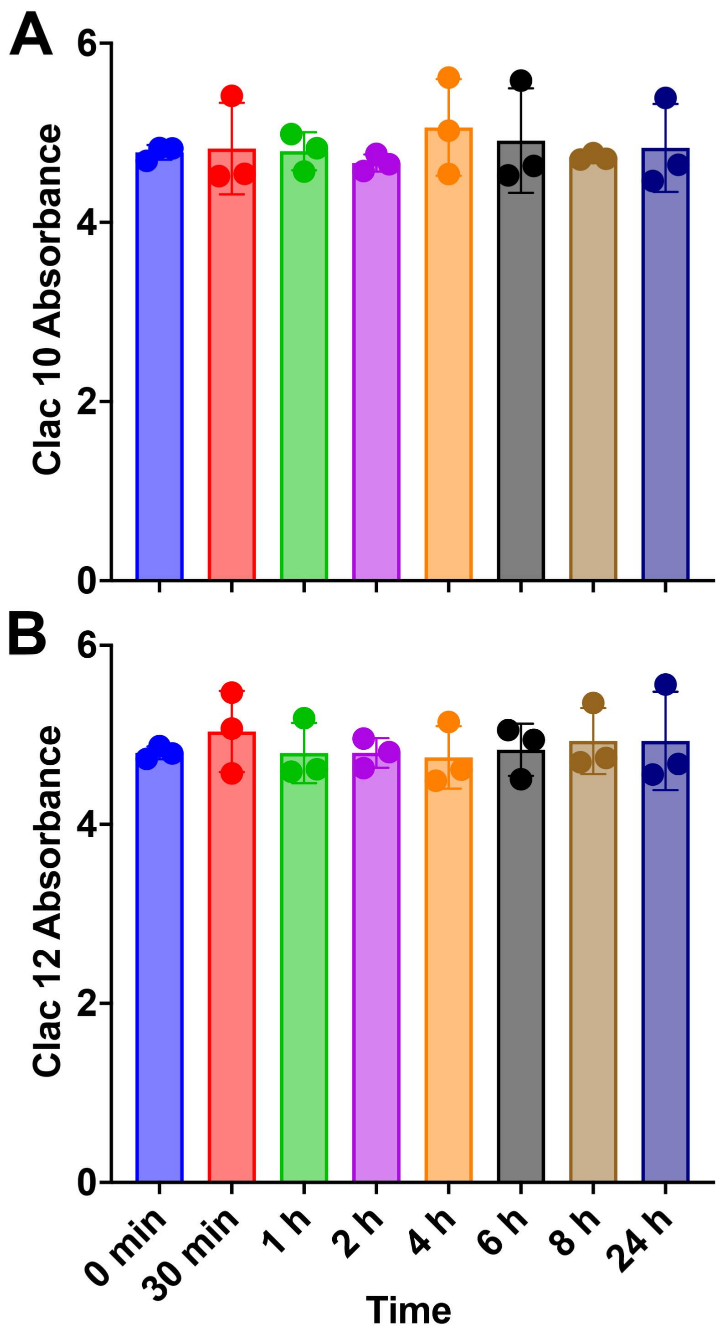

Supplementary Figure 4. Hydrolytic Stability of Compounds **5c** (**Clac 10**) and **5e** (**Clac 12**). UV-visible spectra were used to assess the hydrolytic stability of compounds **5c** and **5e** in PBS solution over a 24-hour period. The spectra indicated minimal hydrolysis, as demonstrated by a slight decrease in the intensity of the absorption bands from 0h to 24h. These results suggest that both compounds **5c** (**Clac 10**) and **5e** (**Clac 12**) are stable in aqueous conditions, highlighting their potential for further in vivo applications. Data are presented as mean±SD at λ_{max} wavelength.

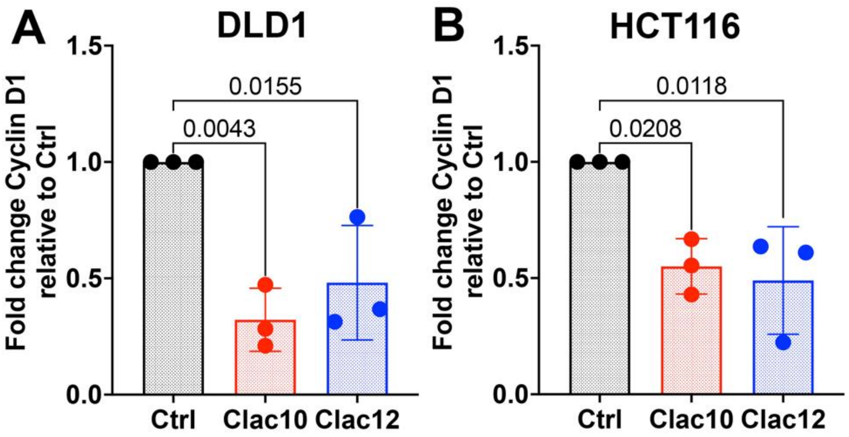

Supplementary Figure 5. Effect of Compounds **5c** (**Clac 10**) and **5e** (**Clac 12**) on Cyclin D1 Expression in HCT116 and DLD1 Cells. Lysates from HCT116 and DLD1 cells treated with IC50 concentrations of compounds **5c** and **5e** for 48 hours were analyzed by western blotting. The results show that both compounds significantly reduced cyclin D1 expression in both cell lines. Data are presented as mean ± standard deviation (SD). Statistical analysis was performed using an ordinary one-way ANOVA, with significance determined at $p < 0.05$.

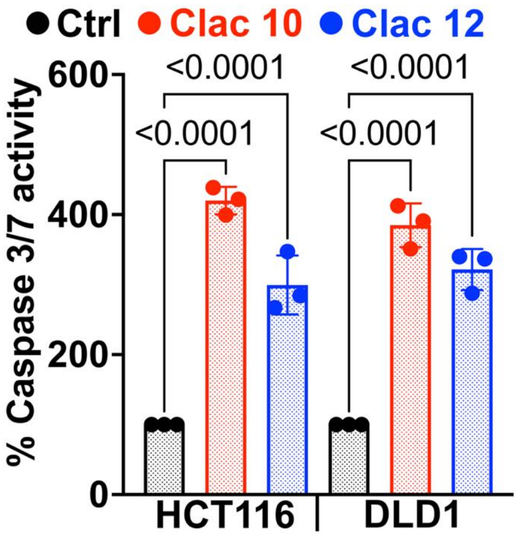

Supplementary Figure 6. Caspase 3/7 Activity in HCT116 and DLD1 Cells Treated with Compounds **5c** (**Clac 10**) and **5e** (**Clac 12**). The Caspase 3/7 activity was measured in HCT116 and DLD1 cells following treatment with compounds **5c** (**Clac 10**) and **5e** (**Clac 12**) at their respective IC_{50} concentrations for 48 hours. The results show a significant increase in Caspase 3/7 activity in both cell lines, indicating enhanced apoptosis. Data are represented as mean ± standard deviation. An ordinary one-way ANOVA was used for statistical comparison.

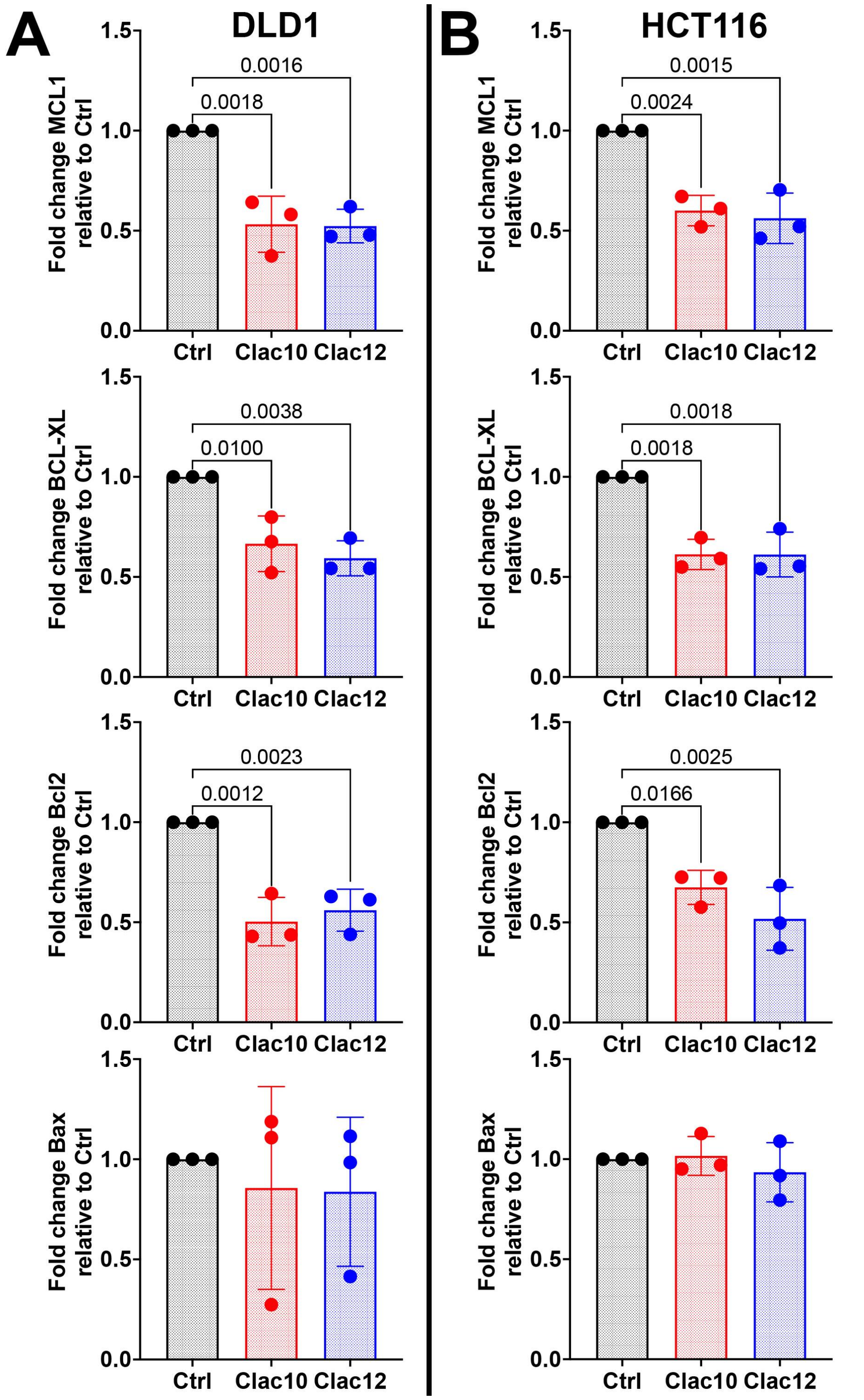

Supplementary Figure 7. Effect of **Clac-10 (5c)** and **Clac-12 (5e)** on Anti-apoptotic Marker Proteins in HCT116 and DLD1 Cells. Lysates from HCT116 and DLD1 cells treated with **Clac-10 (5c)** and **Clac-12 (5e)** showed reduced levels of the anti-apoptotic marker proteins BCL-XL, MCL-1, and BCL-2 compared to untreated controls. Data are presented as mean ± standard deviation (SD). Statistical analysis was performed using an ordinary one-way ANOVA, with significance determined at $p < 0.05$.

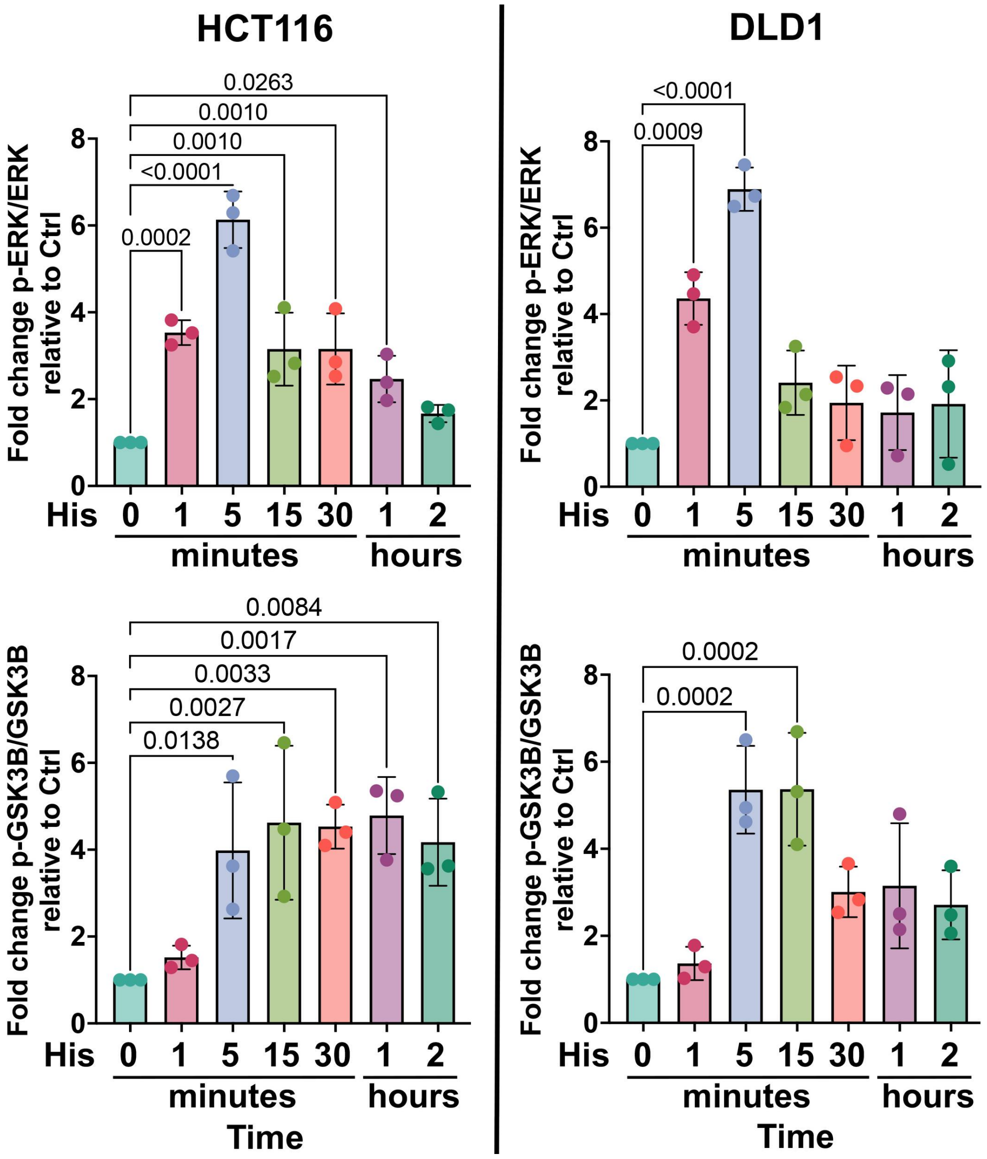

Supplementary Figure 8. Time-dependent Effect of Histamine on ERK1/2 and GSK3B Phosphorylation in CRC Cells. Western blot analysis of lysates from colorectal cancer (CRC) cells, previously synchronized in serum-free media for 24 hours, treated with 10 μM of histamine for up to 2 hours in serum-free media, showed a time-dependent increase in ERK1/2 (Thr202/Tyr204) and GSK3B (Ser-9) phosphorylation. The data show increased phosphorylation of ERK1/2 and GSK3B over the treatment period. Data are presented as mean ± standard deviation (SD). Statistical analysis was performed using an ordinary one-way ANOVA, with significance set at $p < 0.05$.

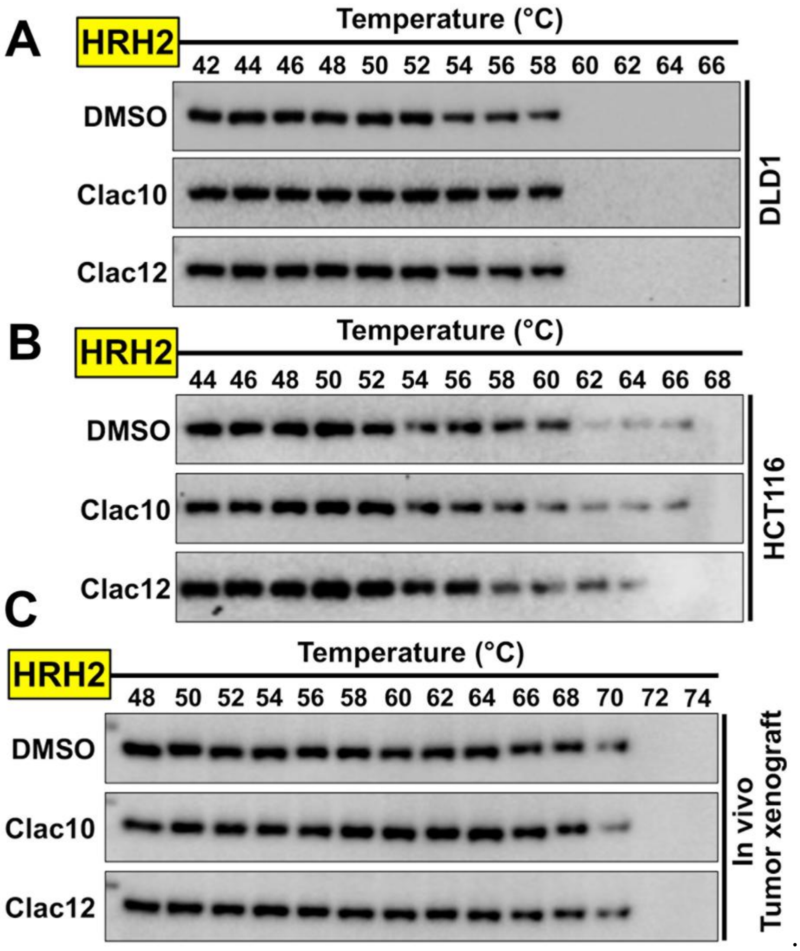

Supplementary Figure 9. The cellular thermal shift assay (CETSA) shows that **5c (Clac10)** and **5e (Clac12)** did not stabilize the HRH2 protein, indicating no binding with HRH2. The compounds were incubated with cell lysates from HCT116 and DLD1 cell lines (A-B) or HCT116-tumor xenograft lysates (C) for four hours, followed by thermal denaturation and evaluation using western blot.

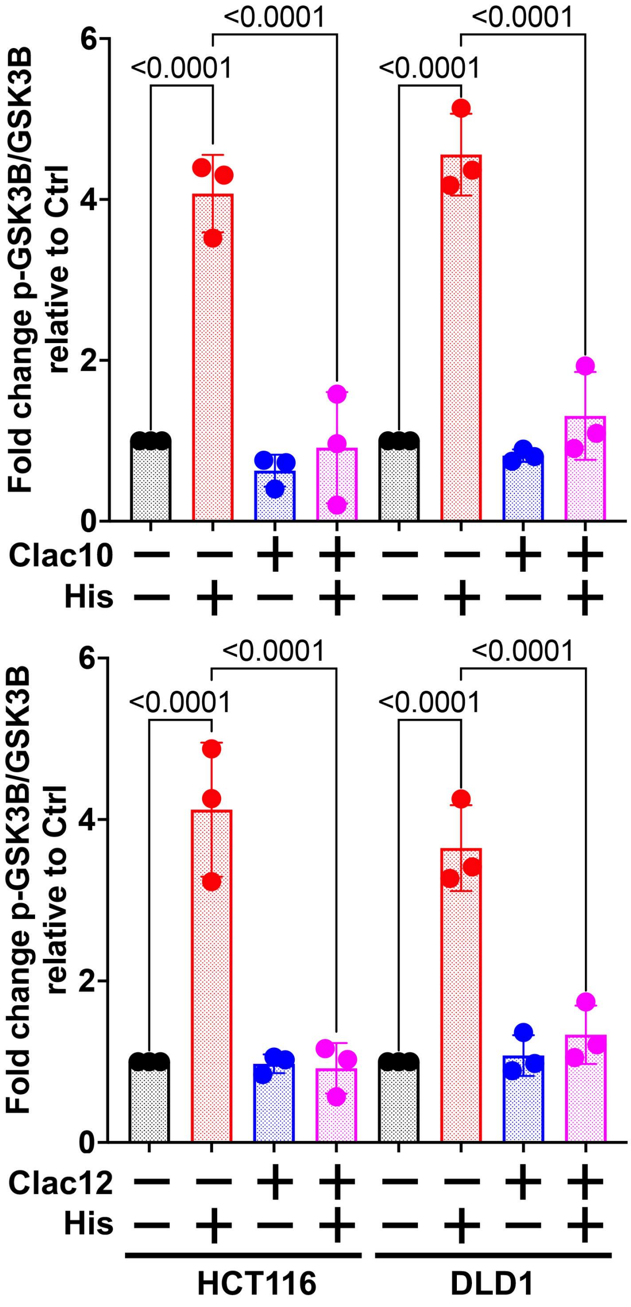

Supplementary Figure 10. Inhibition of Histamine-Induced GSK3B Phosphorylation by Preincubation with **5c (Clac10)** and **5e (Clac12)** in CRC Cells. CRC cells were preincubated with **5c (Clac10)** or **5e (Clac12)** for 4 hours in serum-free media, then treated with 10 μM histamine for 15 min. Western blot analysis showed that preincubation with both 5c and 5e inhibited histamine-induced phosphorylation of GSK3B at Ser-9. Data are presented as mean ± standard deviation (SD). Statistical analysis was performed using an ordinary one-way ANOVA, with significance set at $p < 0.05$.

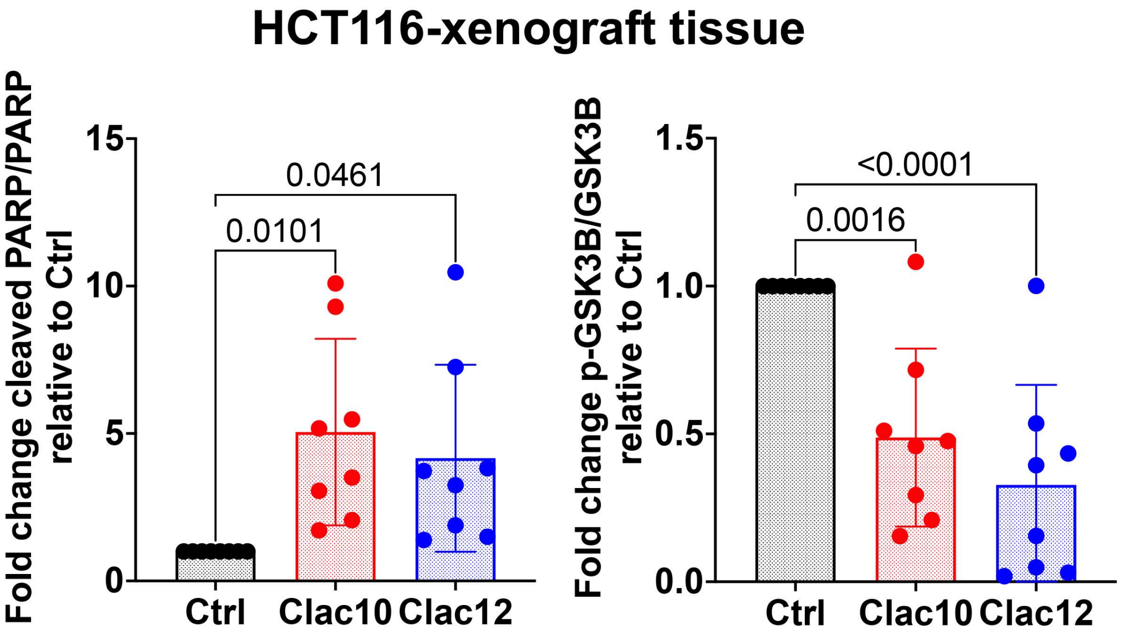

Supplementary Figure 11. Effects of **5c (Clac10)** and **5e (Clac12)** on Apoptosis and Histamine-Signaling in mouse tumor xenograft tissues. Western blot analyses of tissue lysates from mice treated with **5c (Clac10)** and **5e (Clac12)** show significantly higher levels of cleaved PARP, indicating increased apoptosis, and reduced expression of phosphorylated GSK3B (p-GSK3B), indicating inhibition of histamine-signaling. Data are presented as mean ± standard deviation (SD). Statistical analysis was performed using an ordinary one-way ANOVA, with significance set at $p < 0.05$.

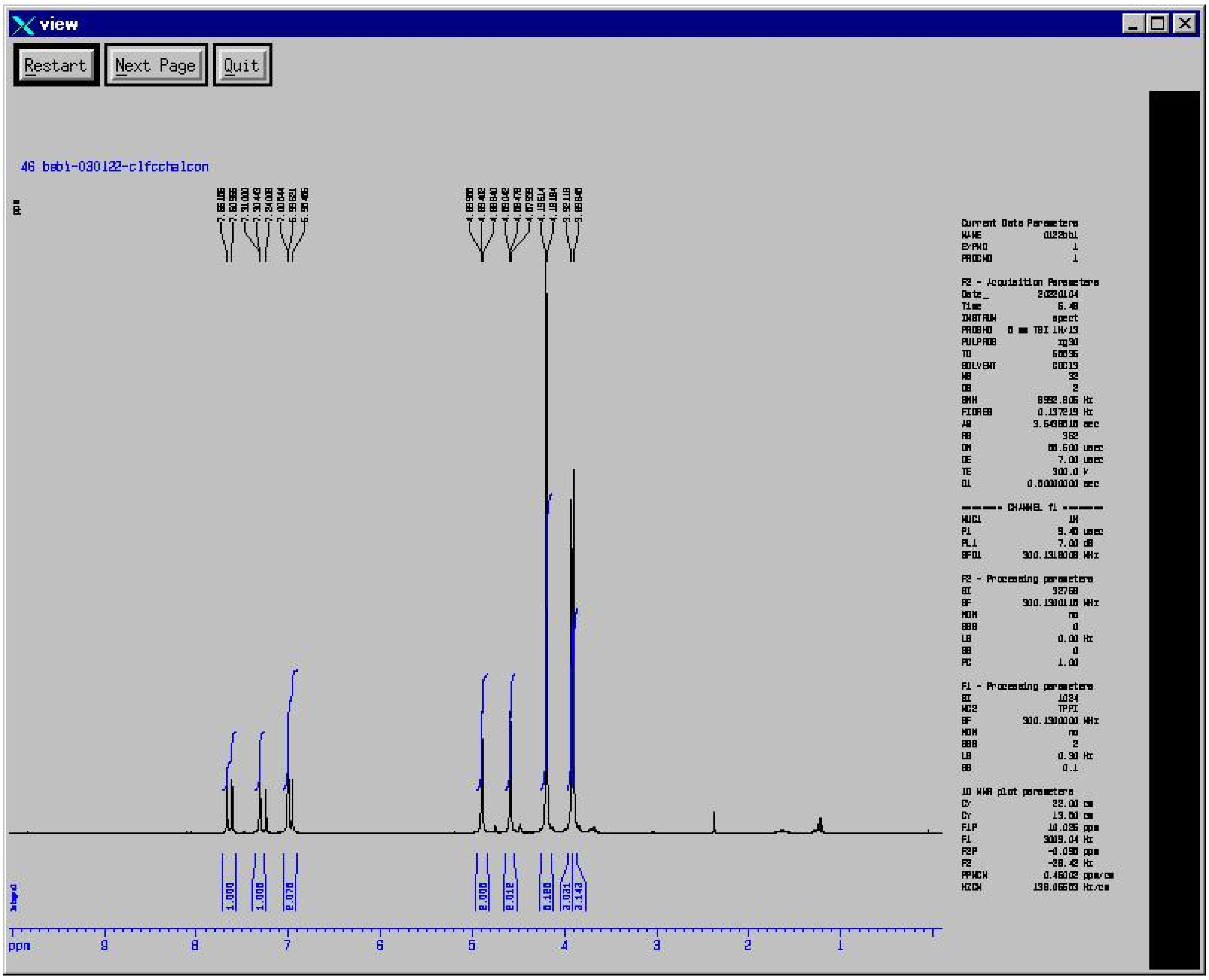

Supplementary Figure 12. ^{1}H NMR spectrum of **2d**.

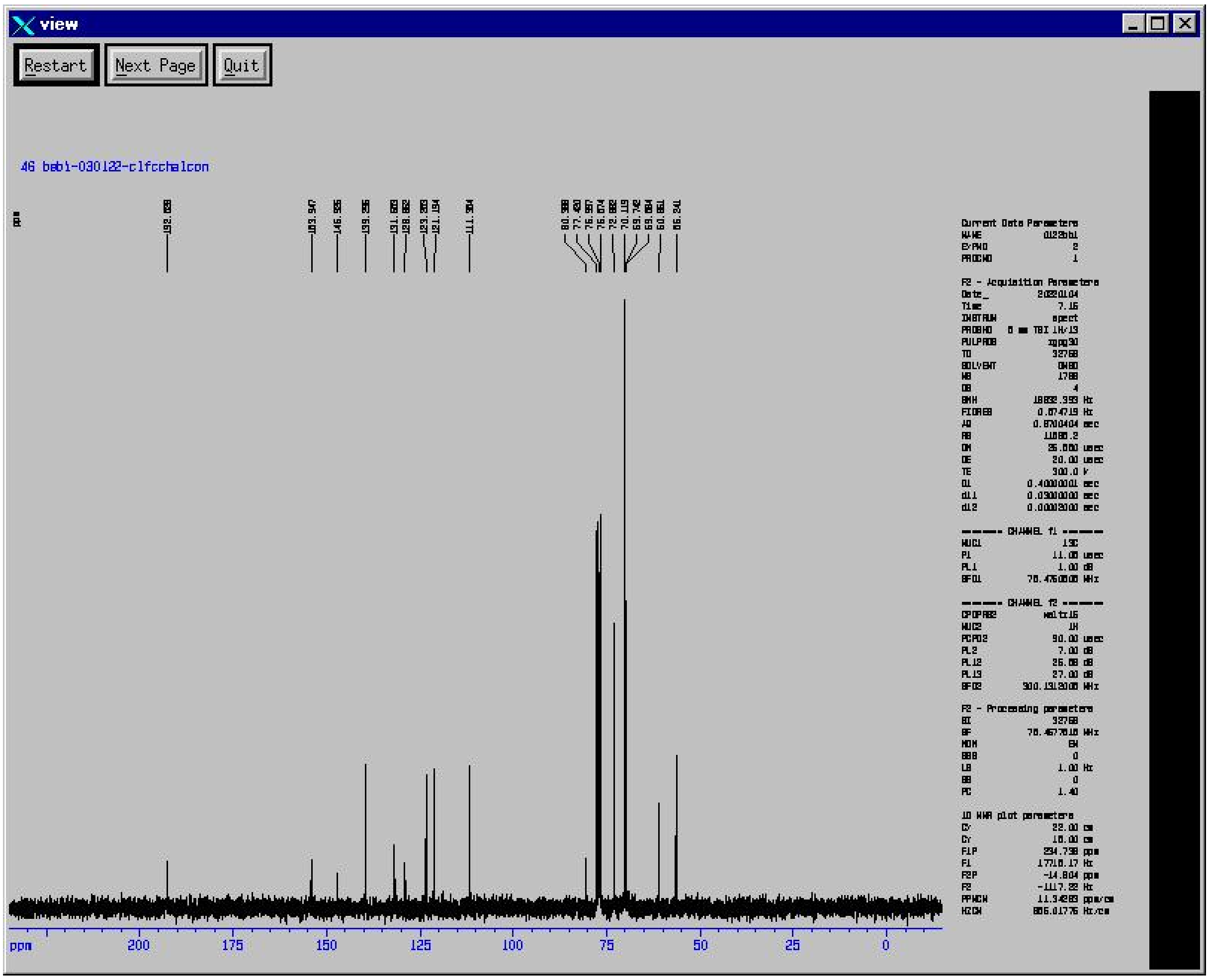

Supplementary Figure 13. ^{13}C NMR spectrum of **2d**.

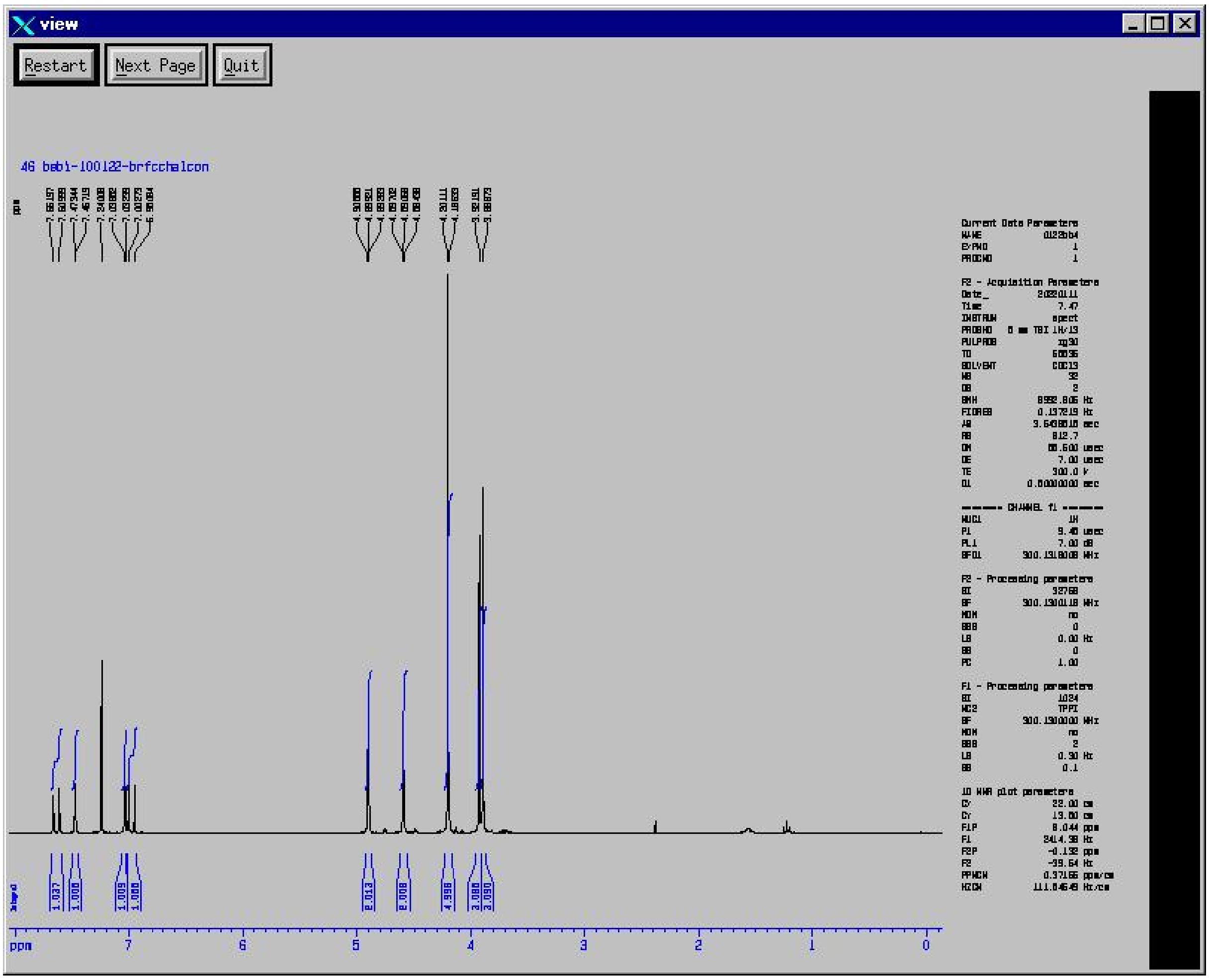

Supplementary Figure 14. ^{1}H NMR spectrum of **2e**.

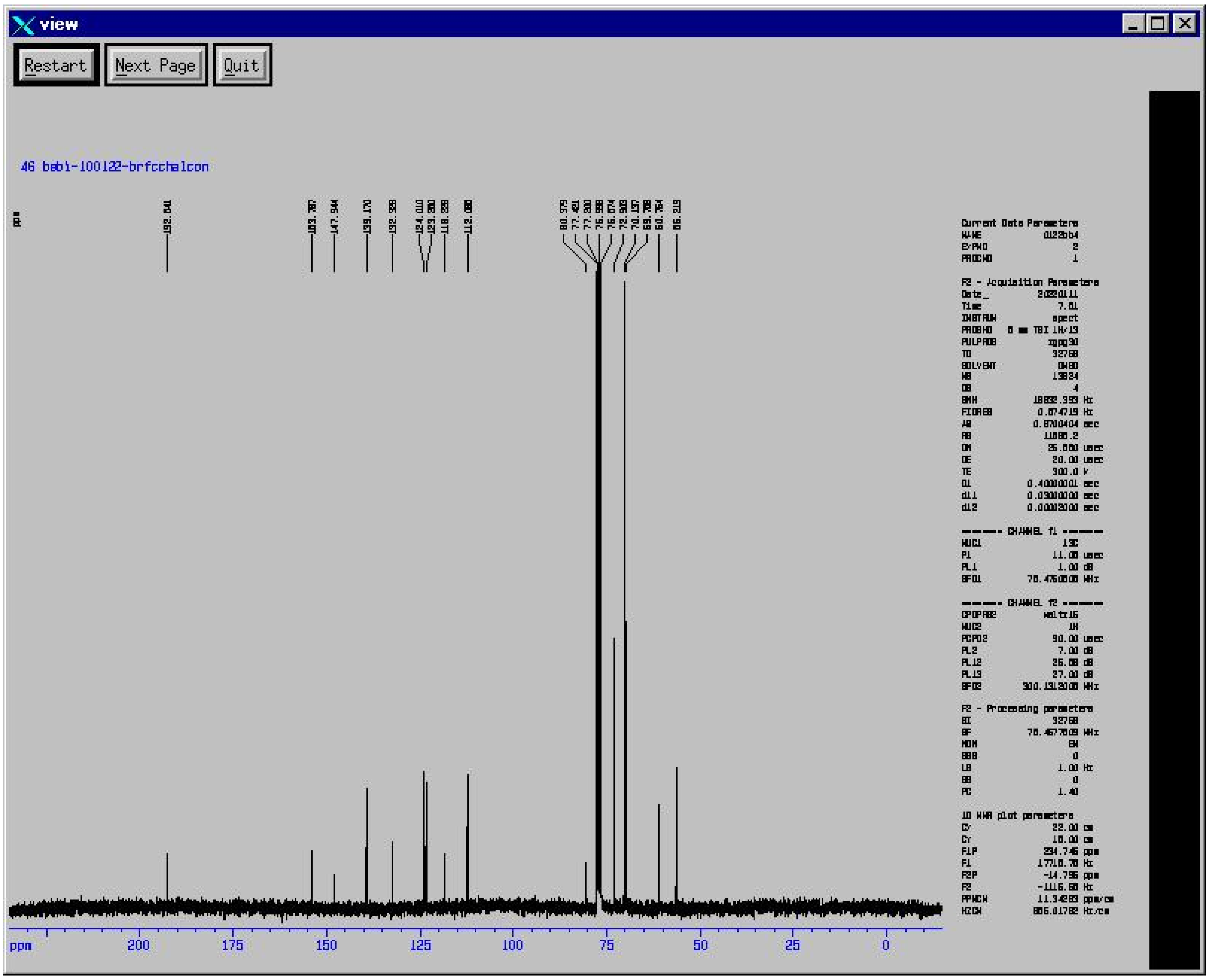

Supplementary Figure 15. ^{13}C NMR spectrum of **2e**.

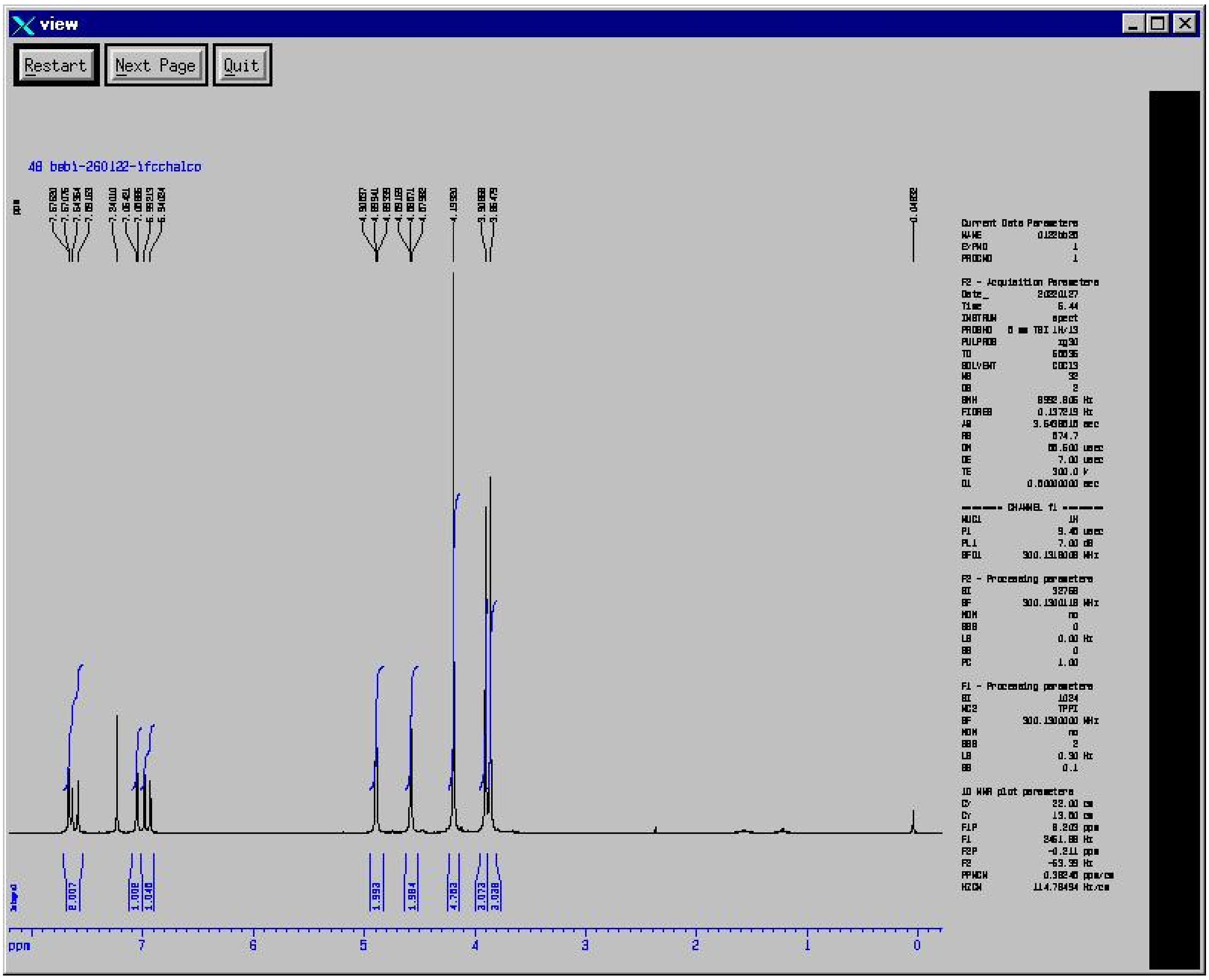

Supplementary Figure 16. ^{1}H NMR spectrum of **2f**.

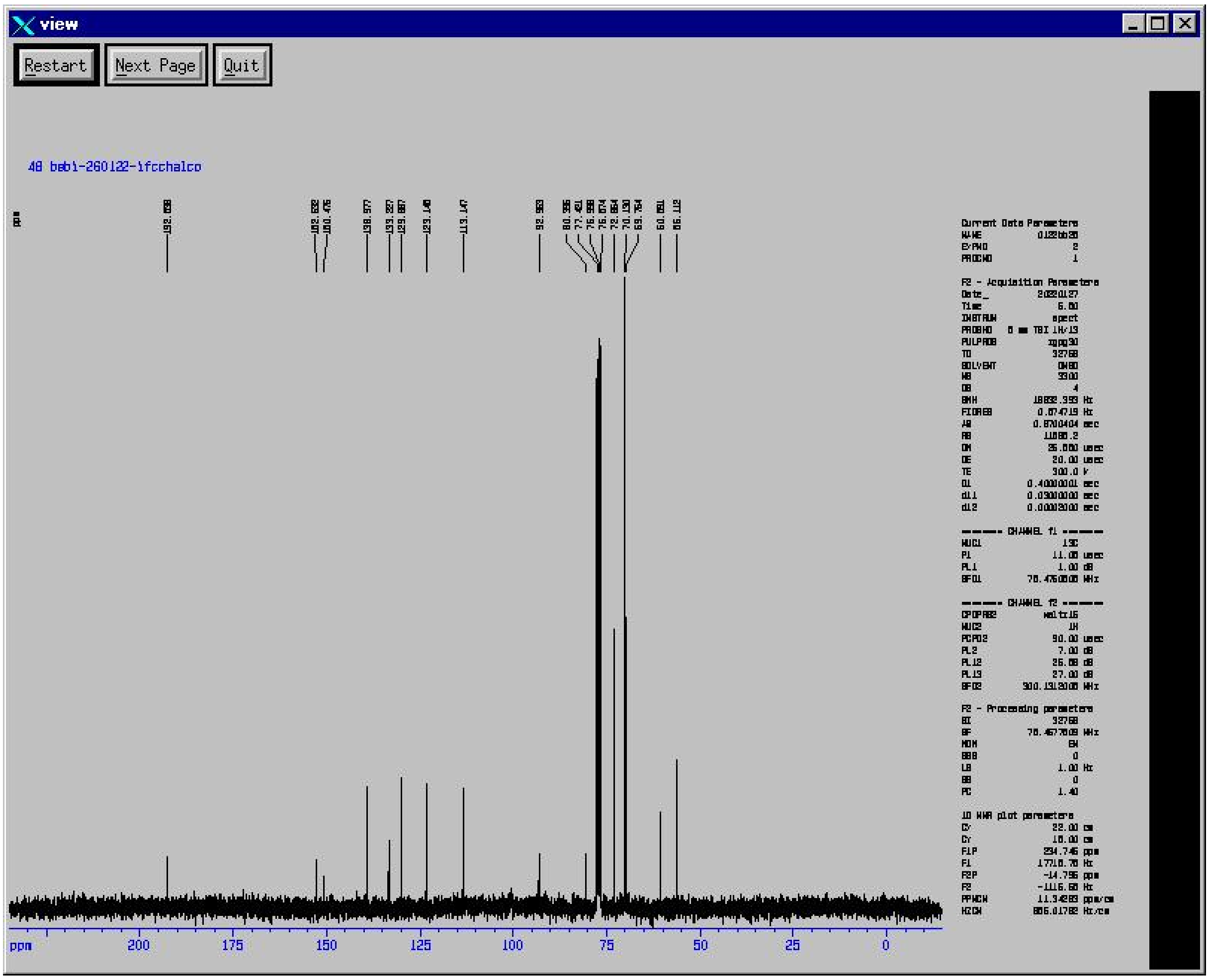

Supplementary Figure 17. ^{13}C NMR spectrum of **2f**.

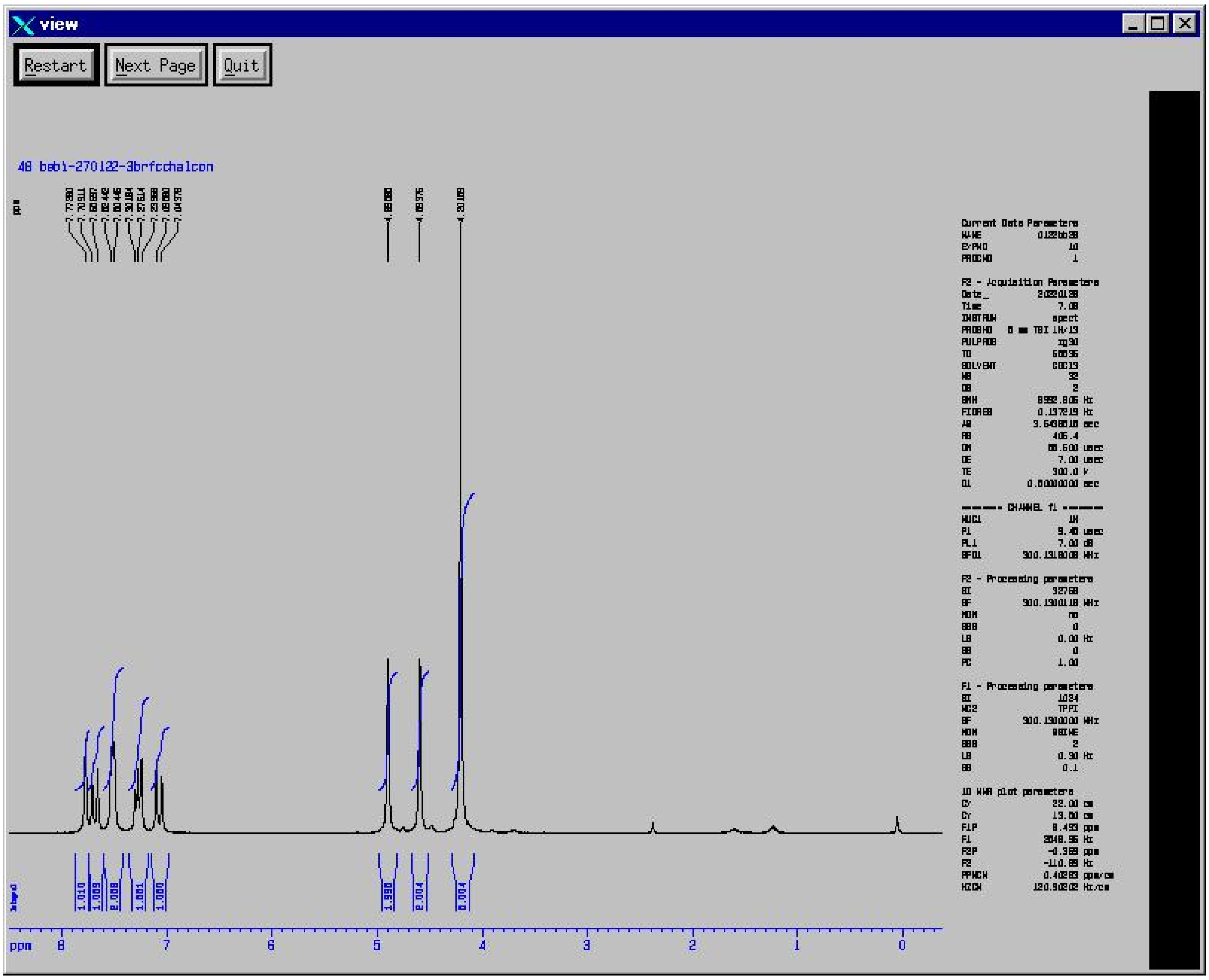

Supplementary Figure 18. ^{1}H NMR spectrum of **2g**.

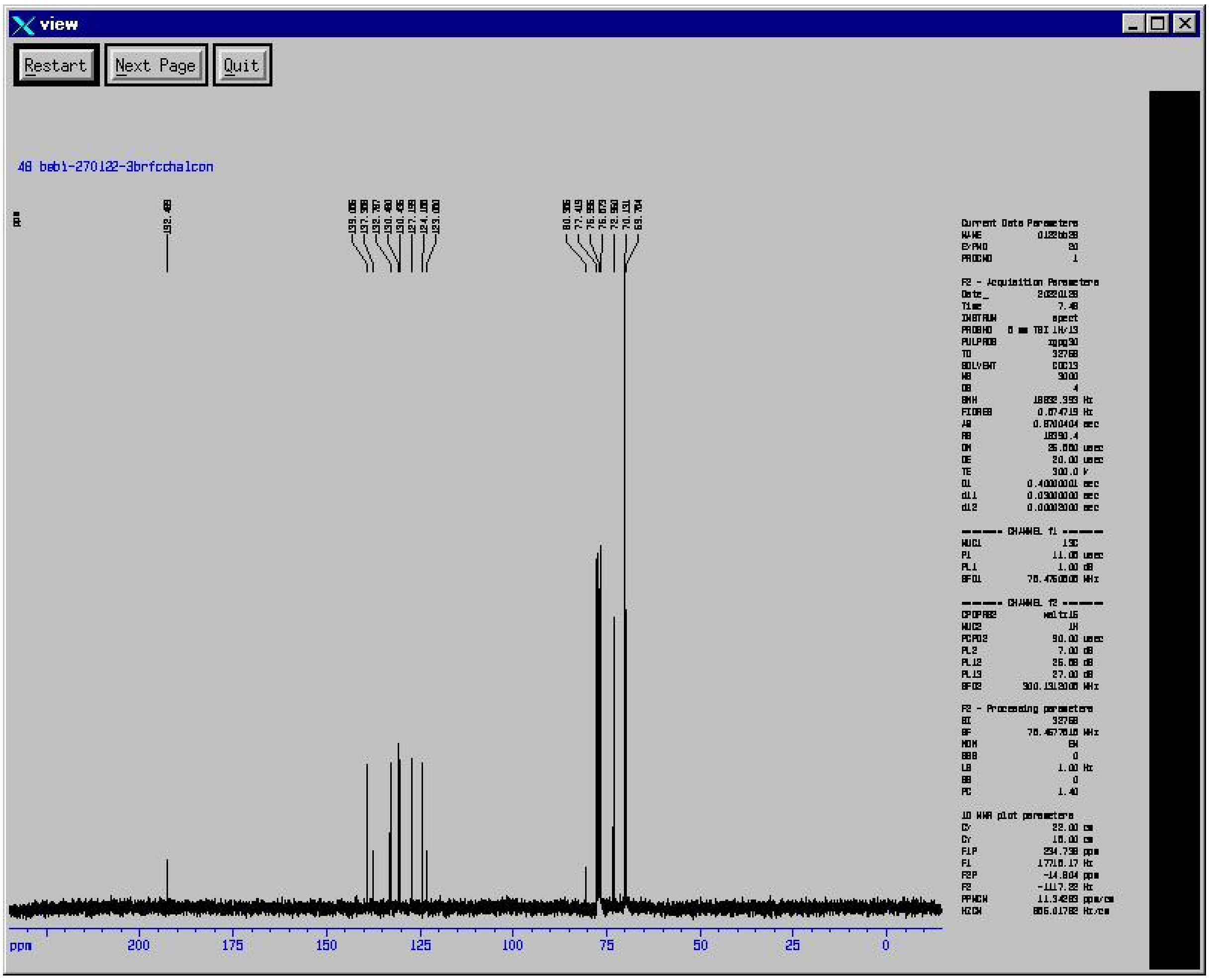

Supplementary Figure 19. ^{13}C NMR spectrum of **2g**.

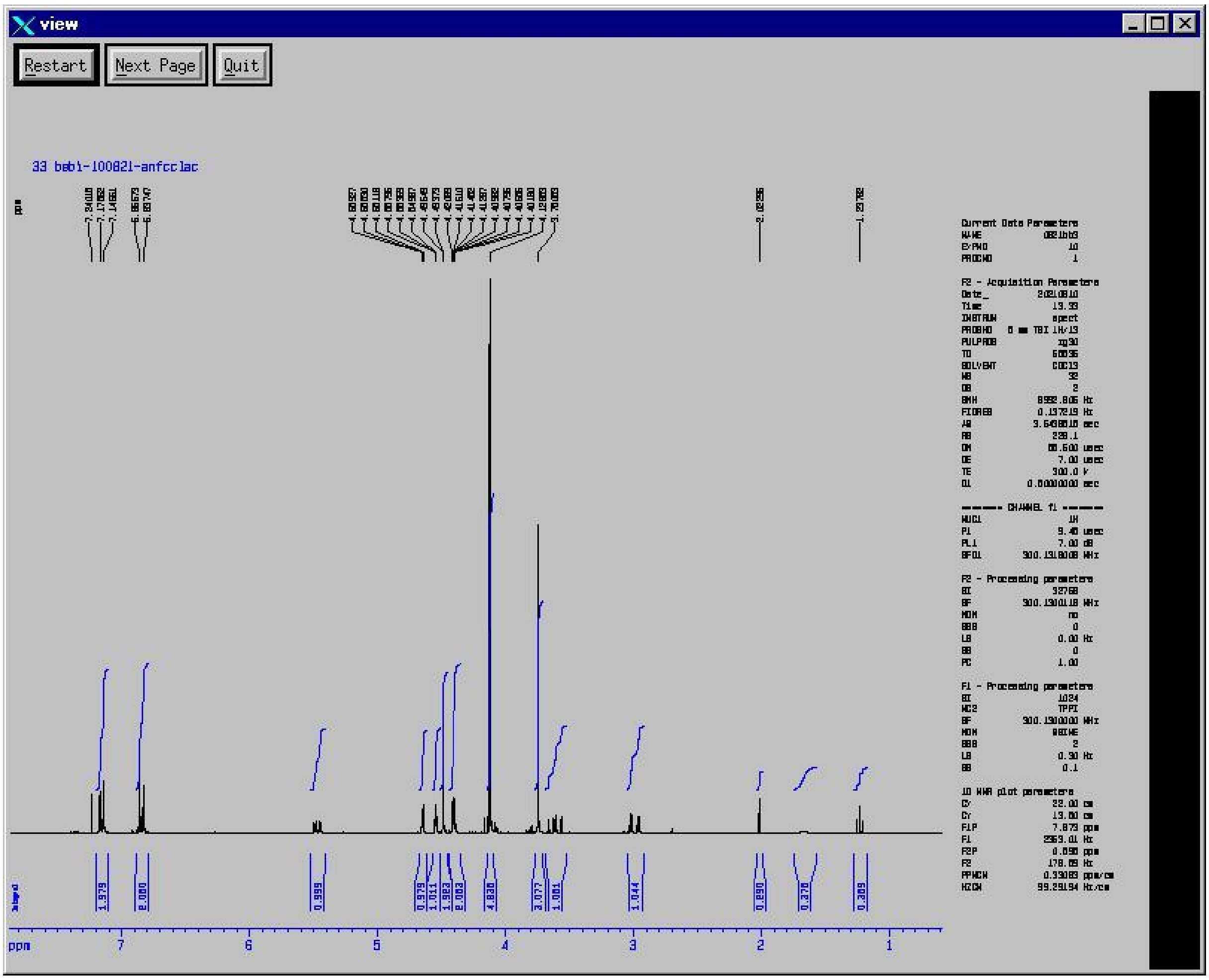

Supplementary Figure 20. ^{1}H NMR spectrum of **4a**.

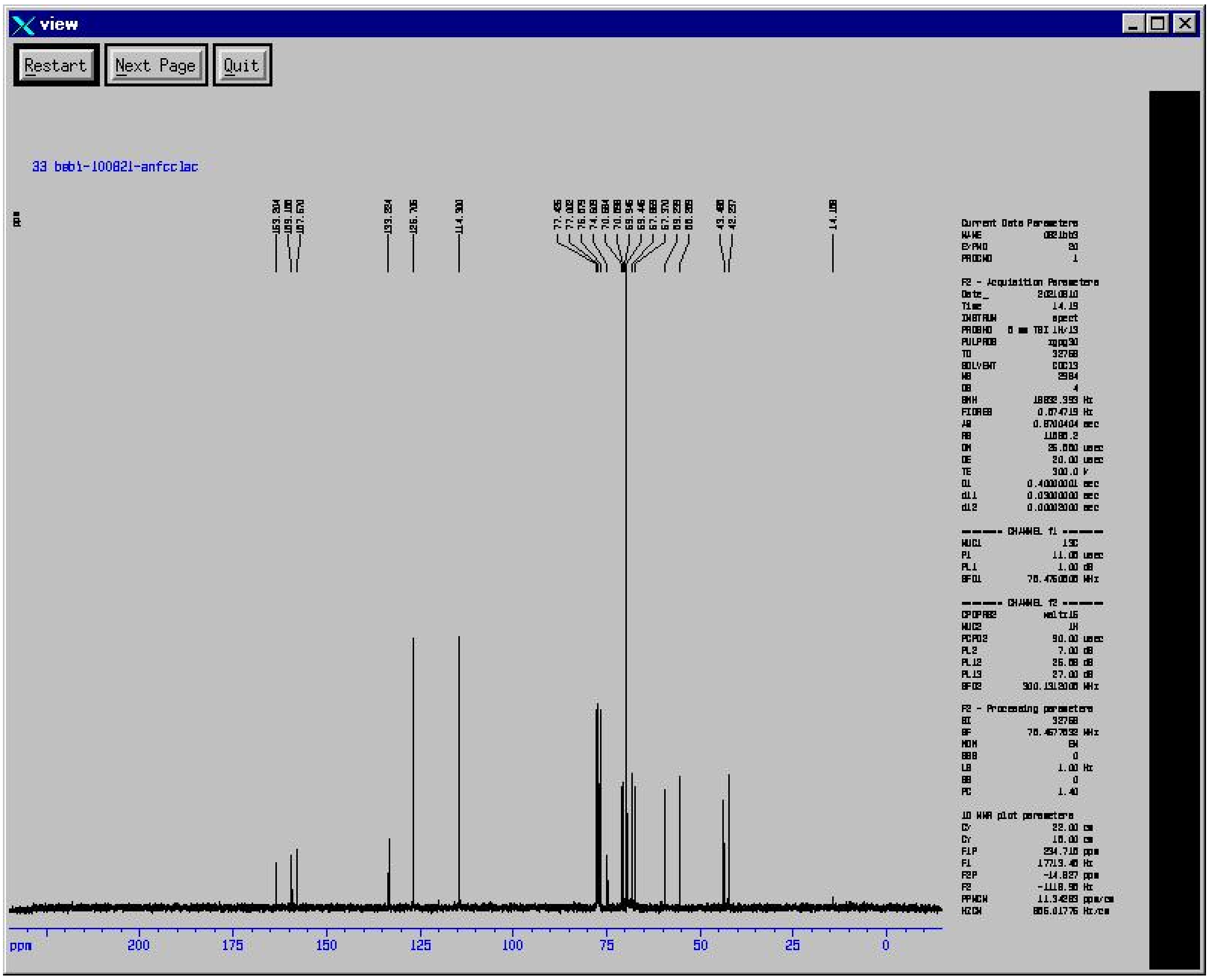

Supplementary Figure 21. ^{13}C NMR spectrum of **4a**.

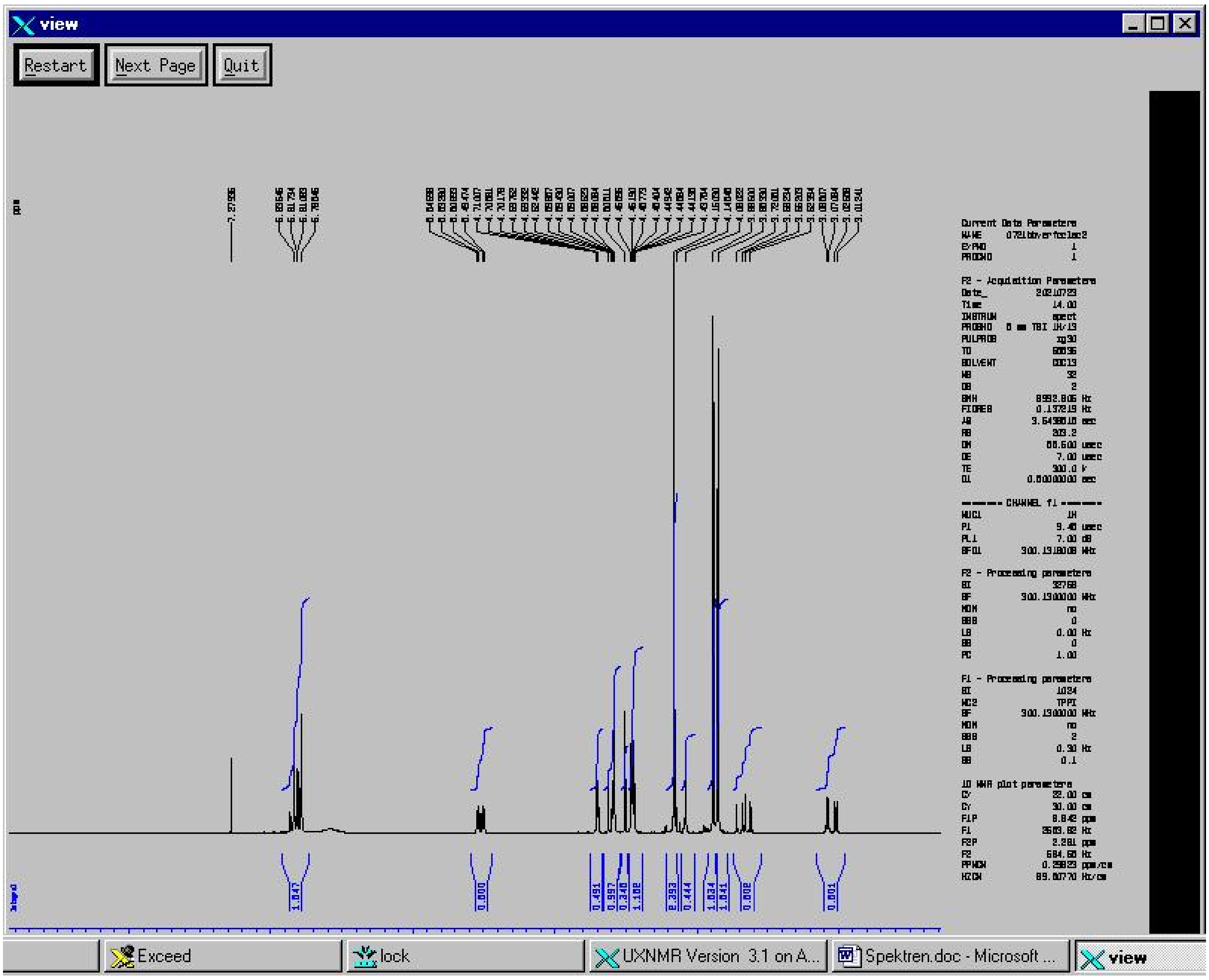

Supplementary Figure 22. ^{1}H NMR spectrum of **4b**.

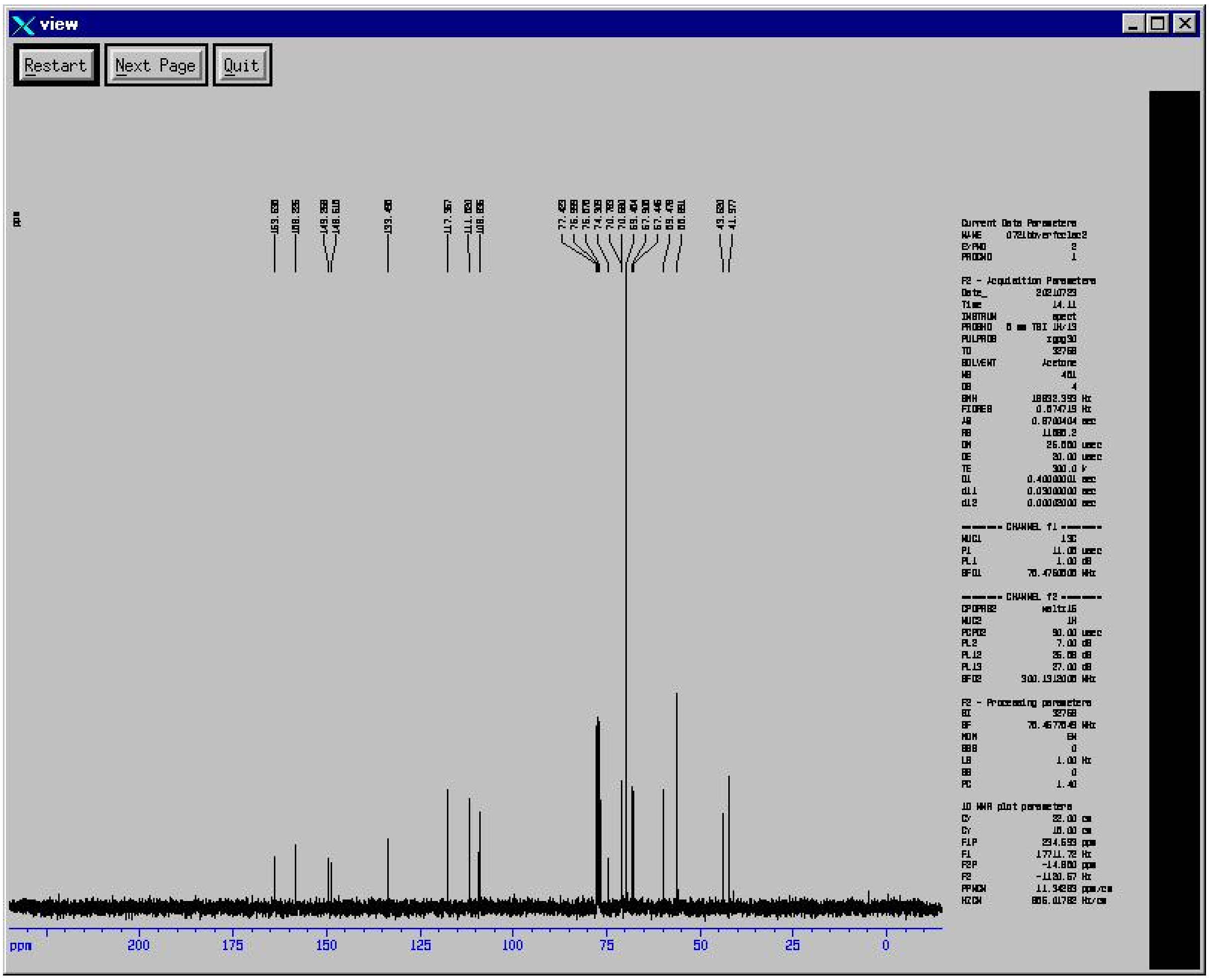

Supplementary Figure 23. ^{13}C NMR spectrum of **4b**.

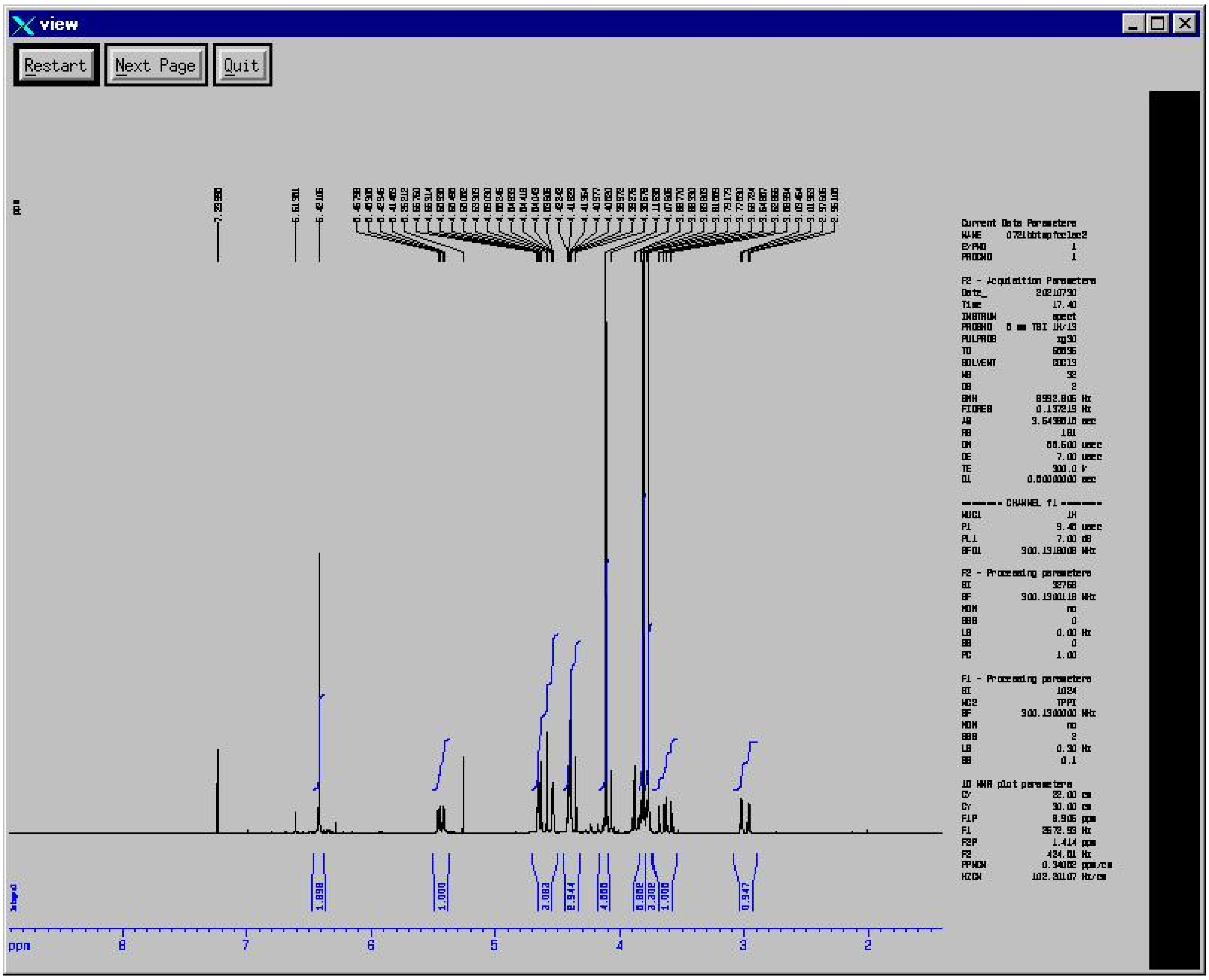

Supplementary Figure 24. ^{1}H NMR spectrum of **4c**.

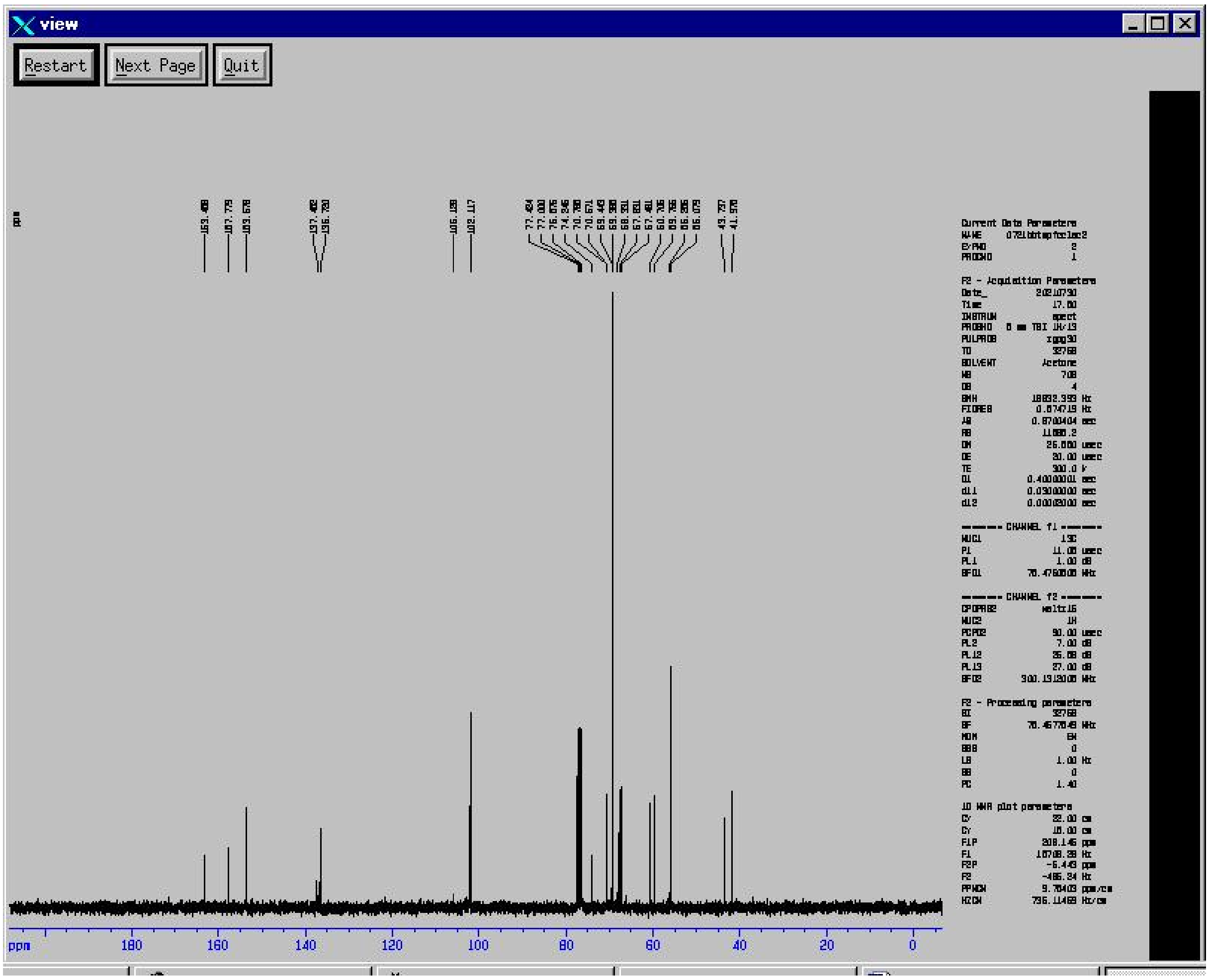

Supplementary Figure 25. ^{13}C NMR spectrum of **4c**.

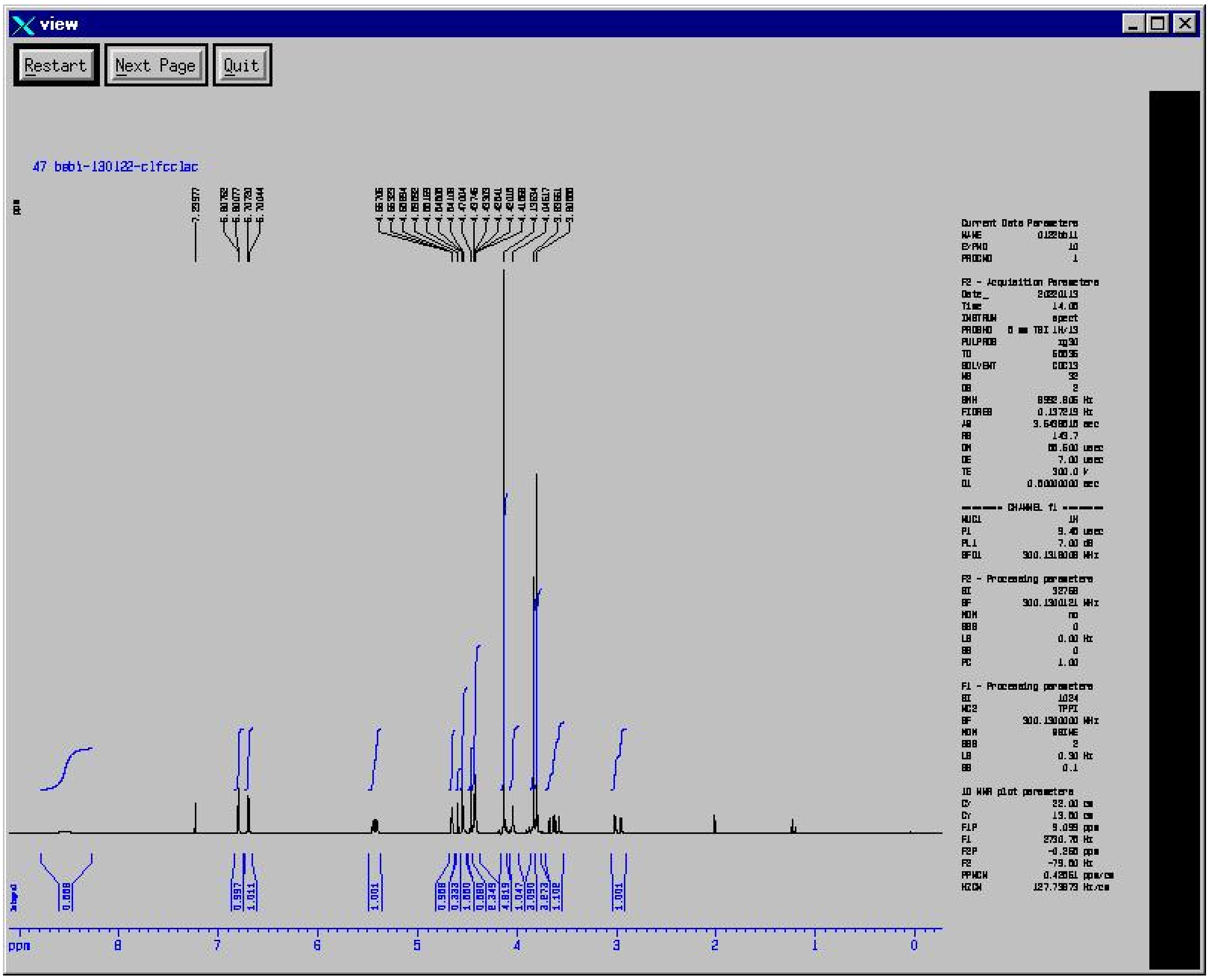

Supplementary Figure 26. ^{1}H NMR spectrum of **4d**.

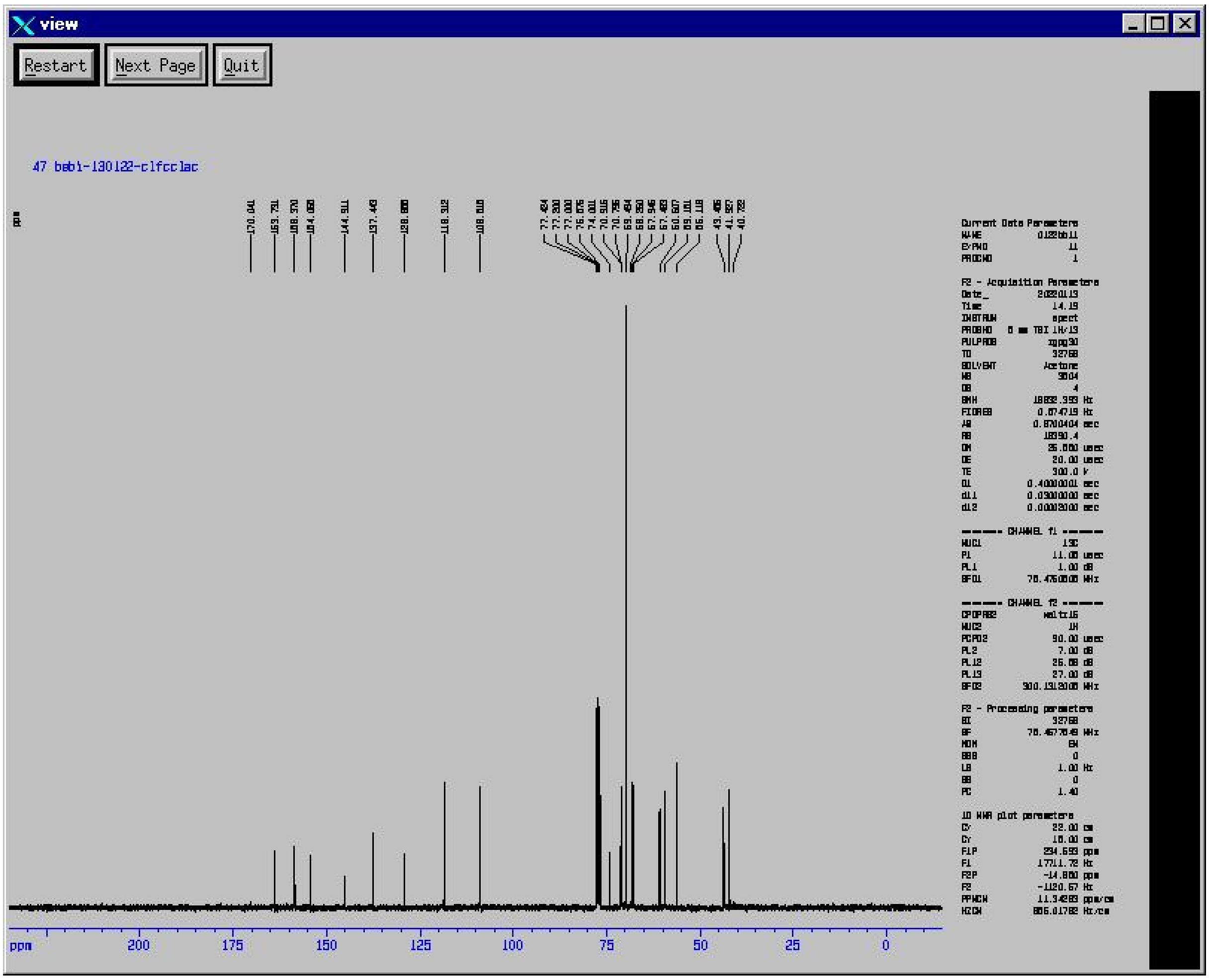

Supplementary Figure 27. ^{13}C NMR spectrum of **4d**.

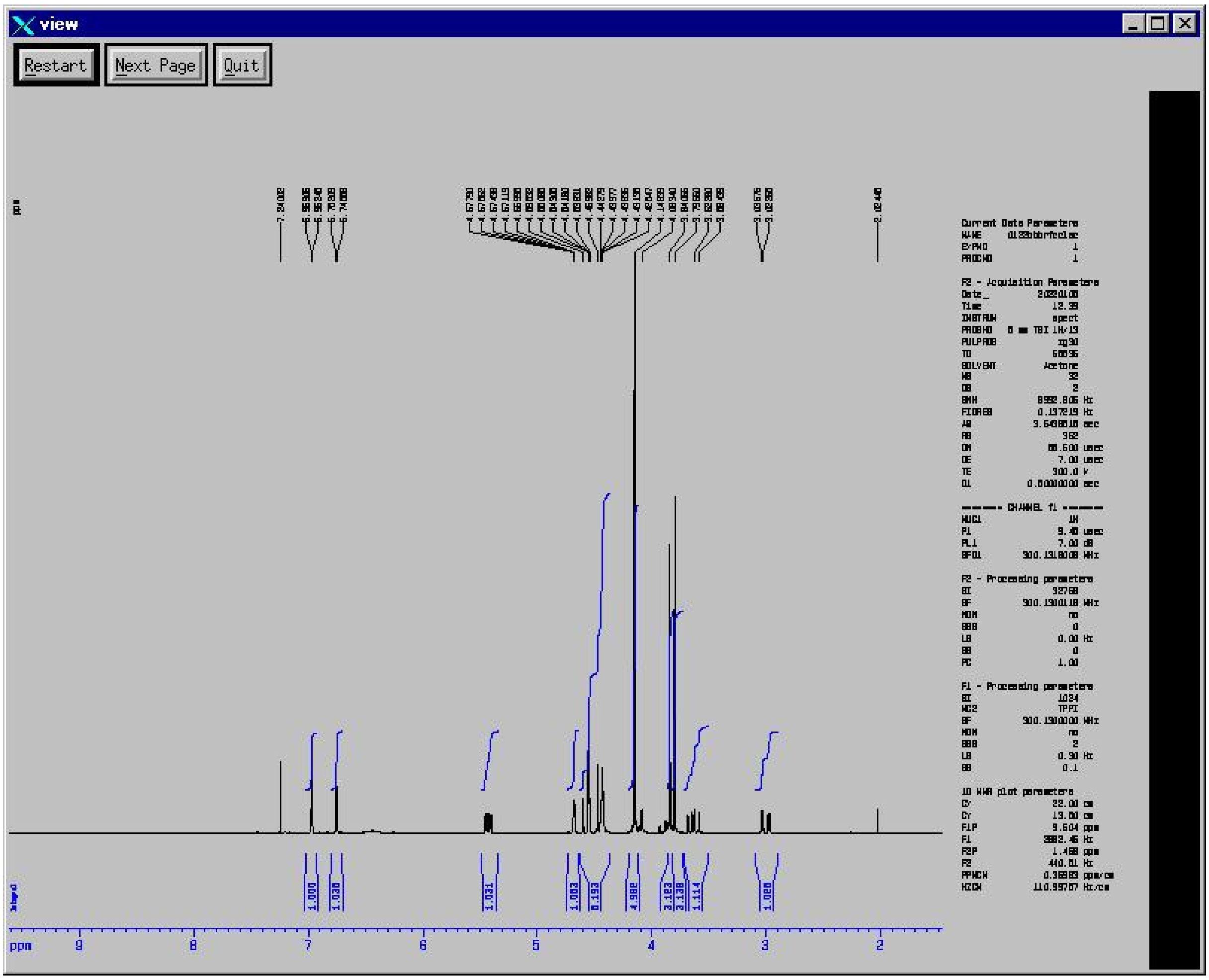

Supplementary Figure 28. ^{1}H NMR spectrum of **4e**.

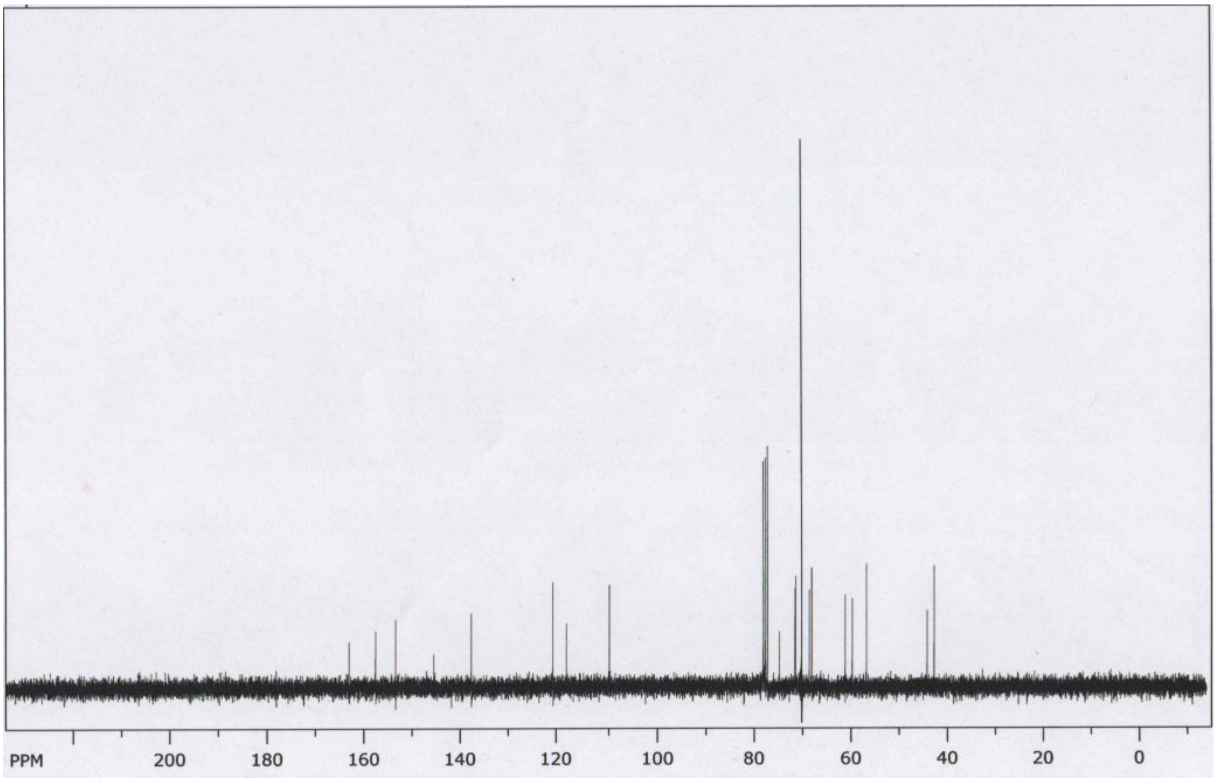

Supplementary Figure 29. 13C NMR spectrum of **4e**.

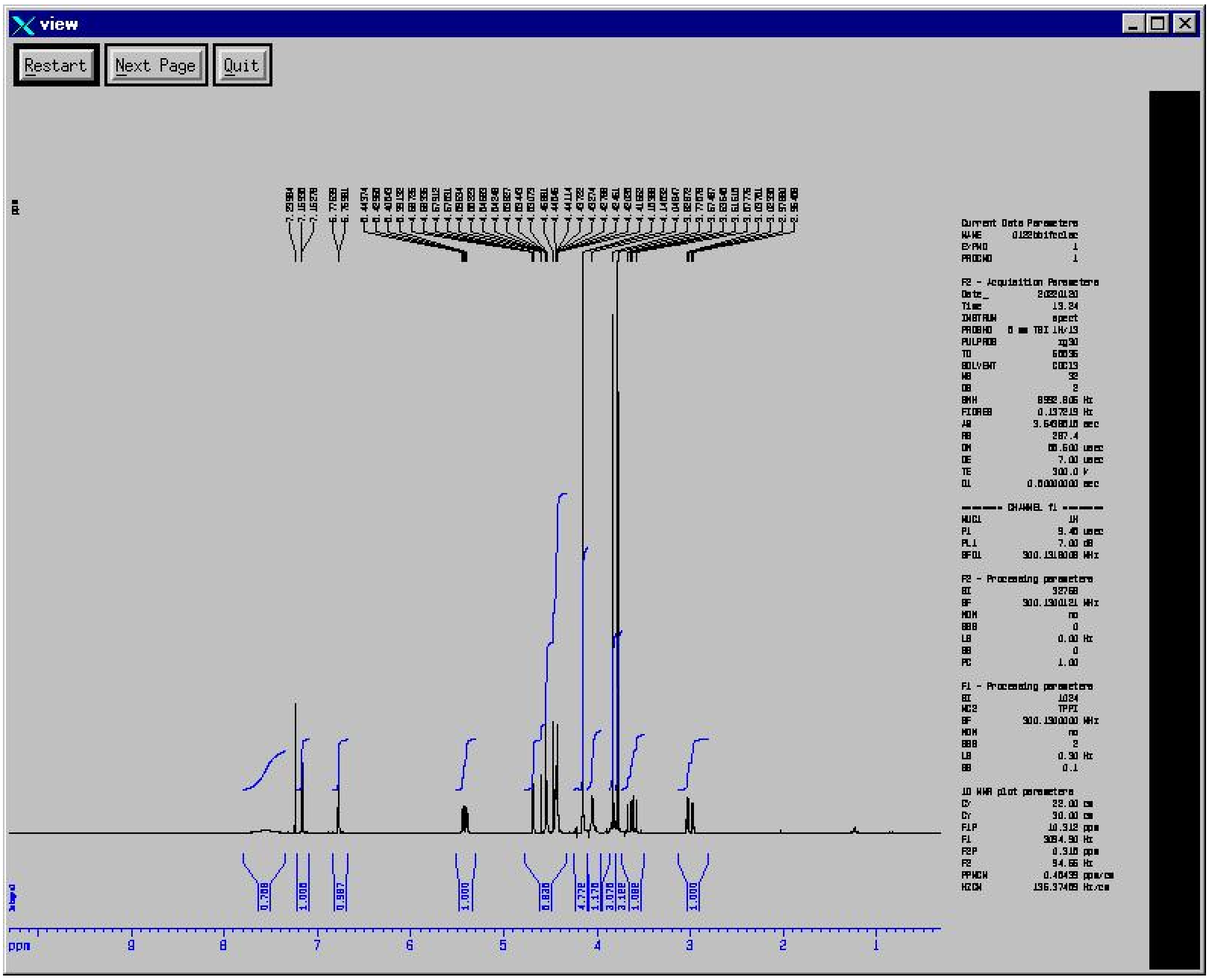

Supplementary Figure 30. ^{1}H NMR spectrum of **4f**.

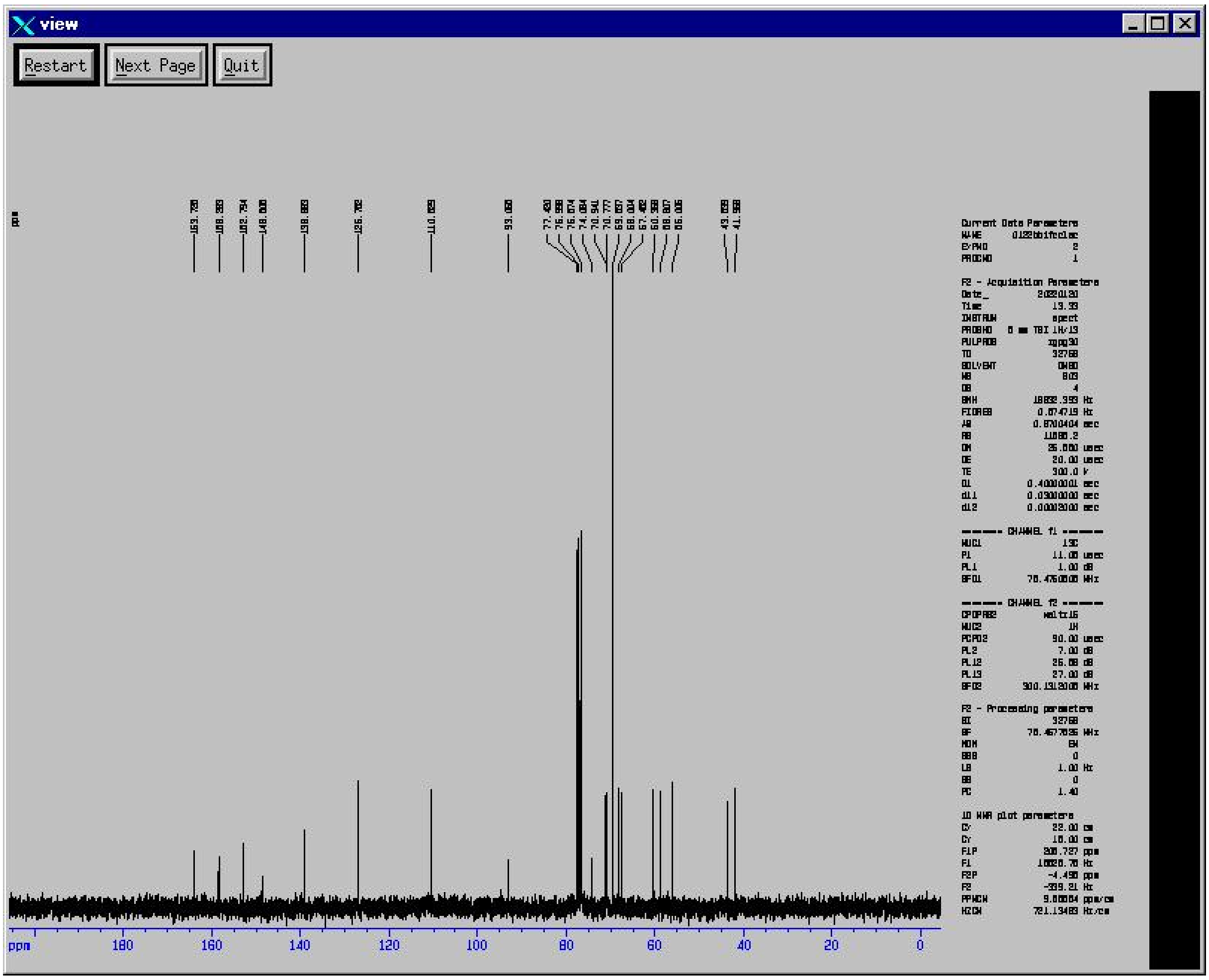

Supplementary Figure 31. ^{13}C NMR spectrum of **4f**.

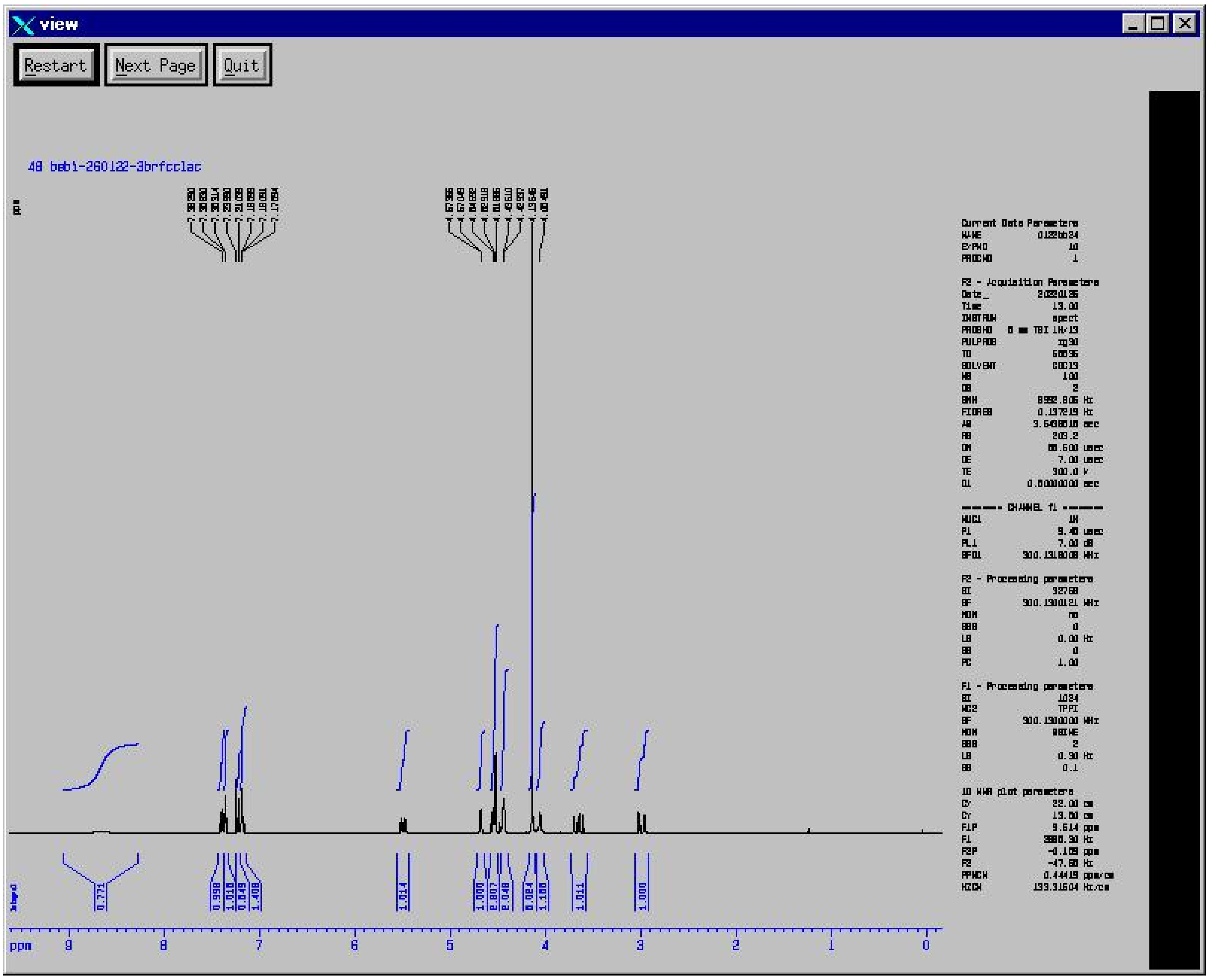

Supplementary Figure 32. ^{1}H NMR spectrum of **4g**.

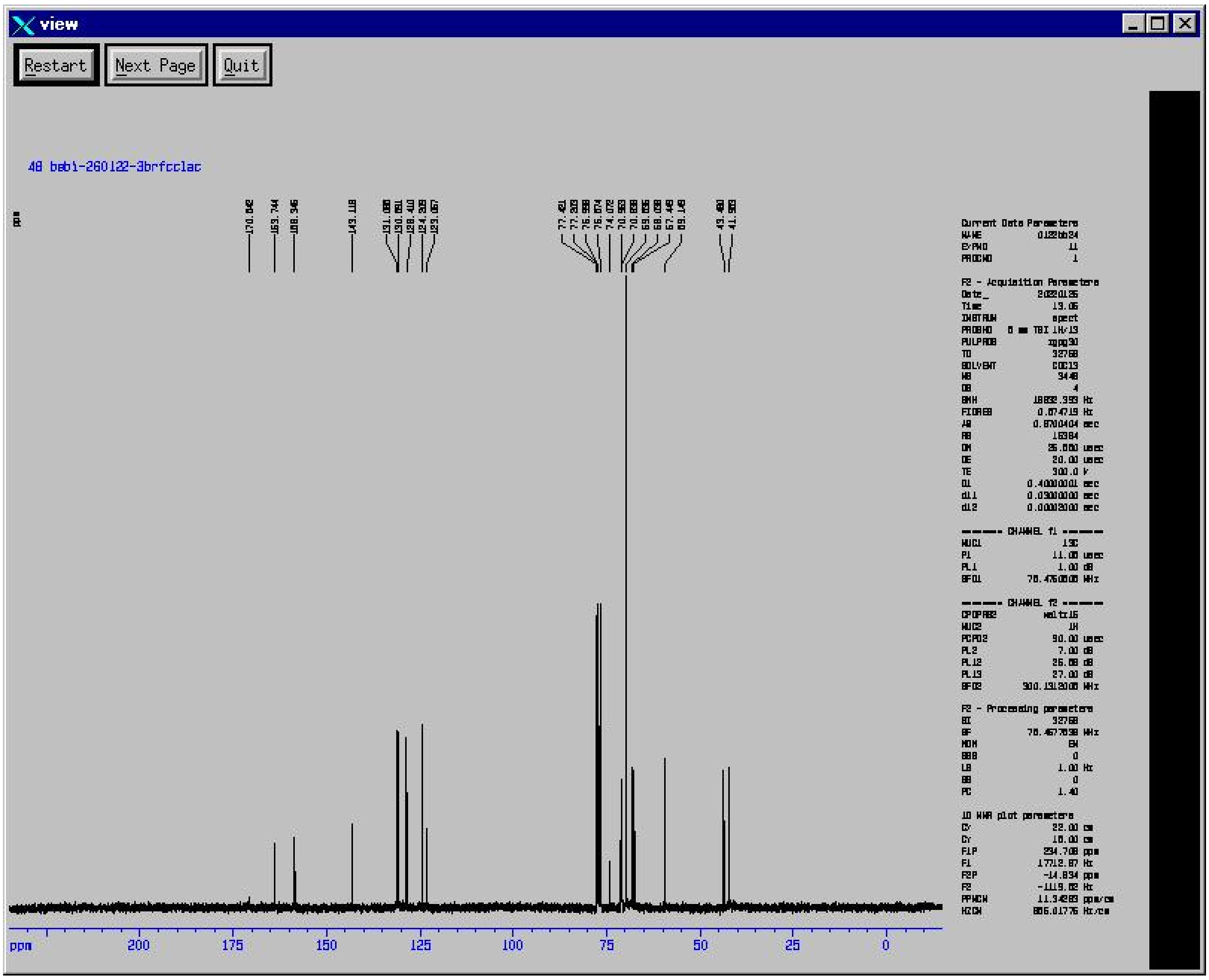

Supplementary Figure 33. ^{13}C NMR spectrum of **4g**.

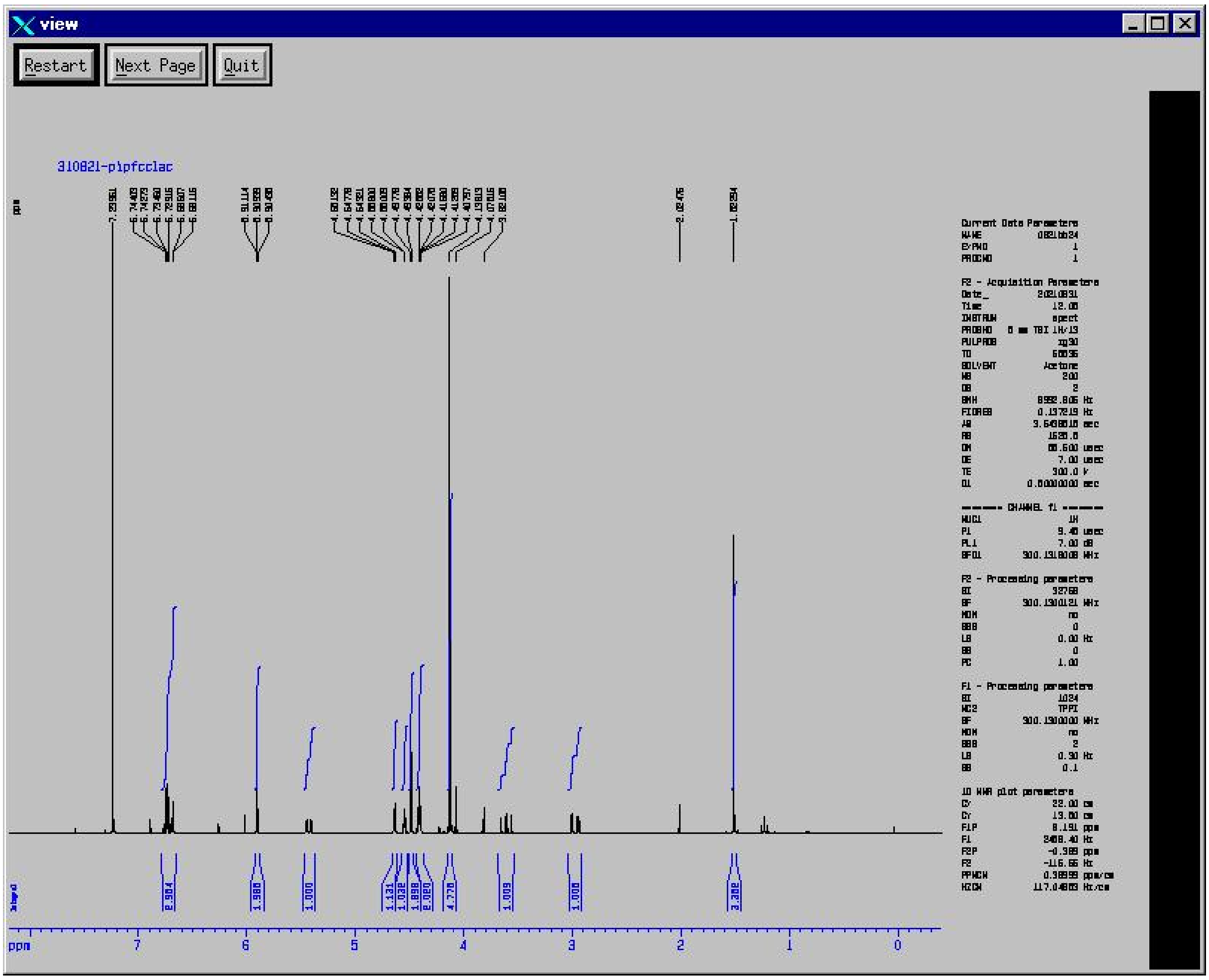

Supplementary Figure 34. ^{1}H NMR spectrum of **4h**.

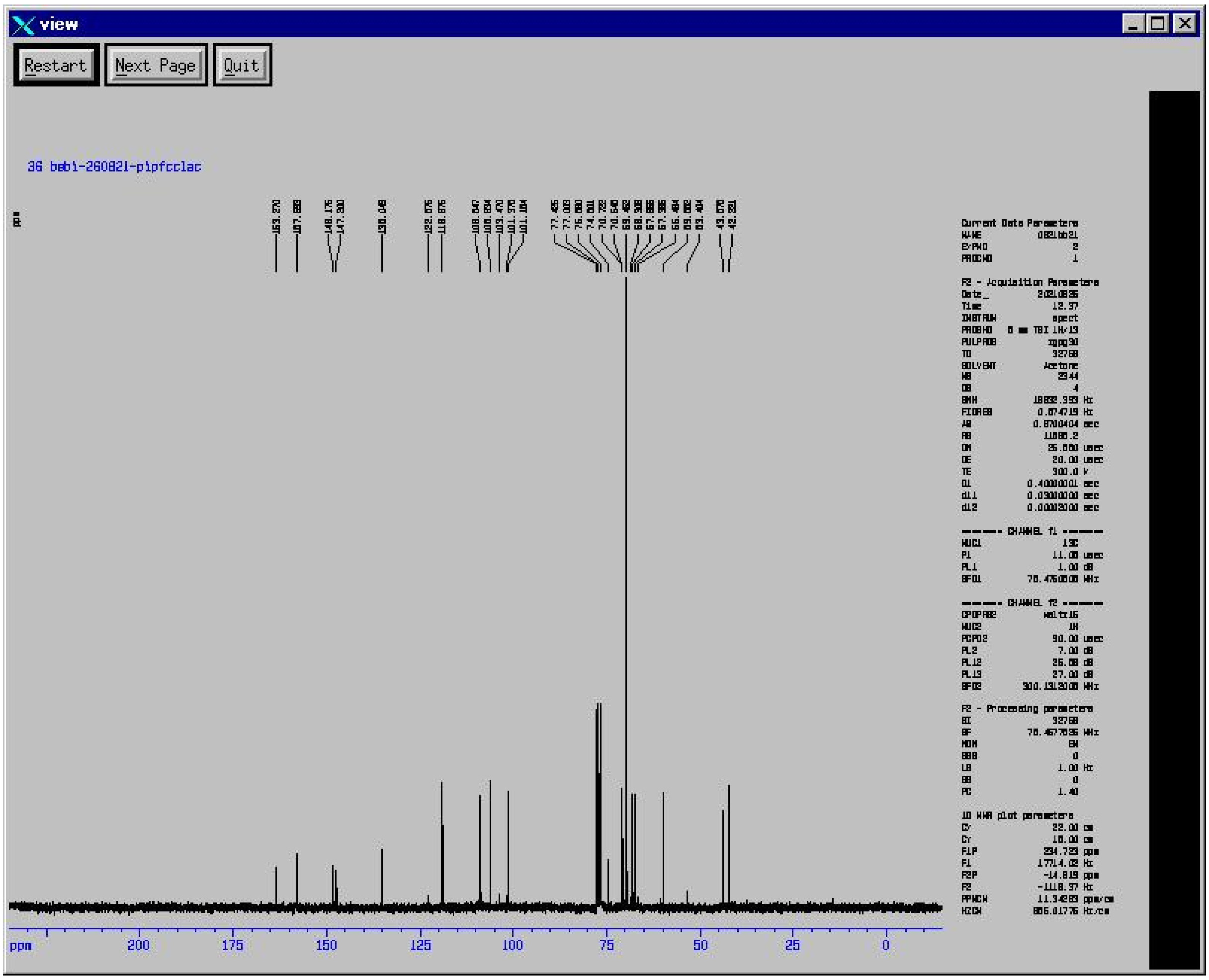

Supplementary Figure 35. ^{13}C NMR spectrum of **4h**.

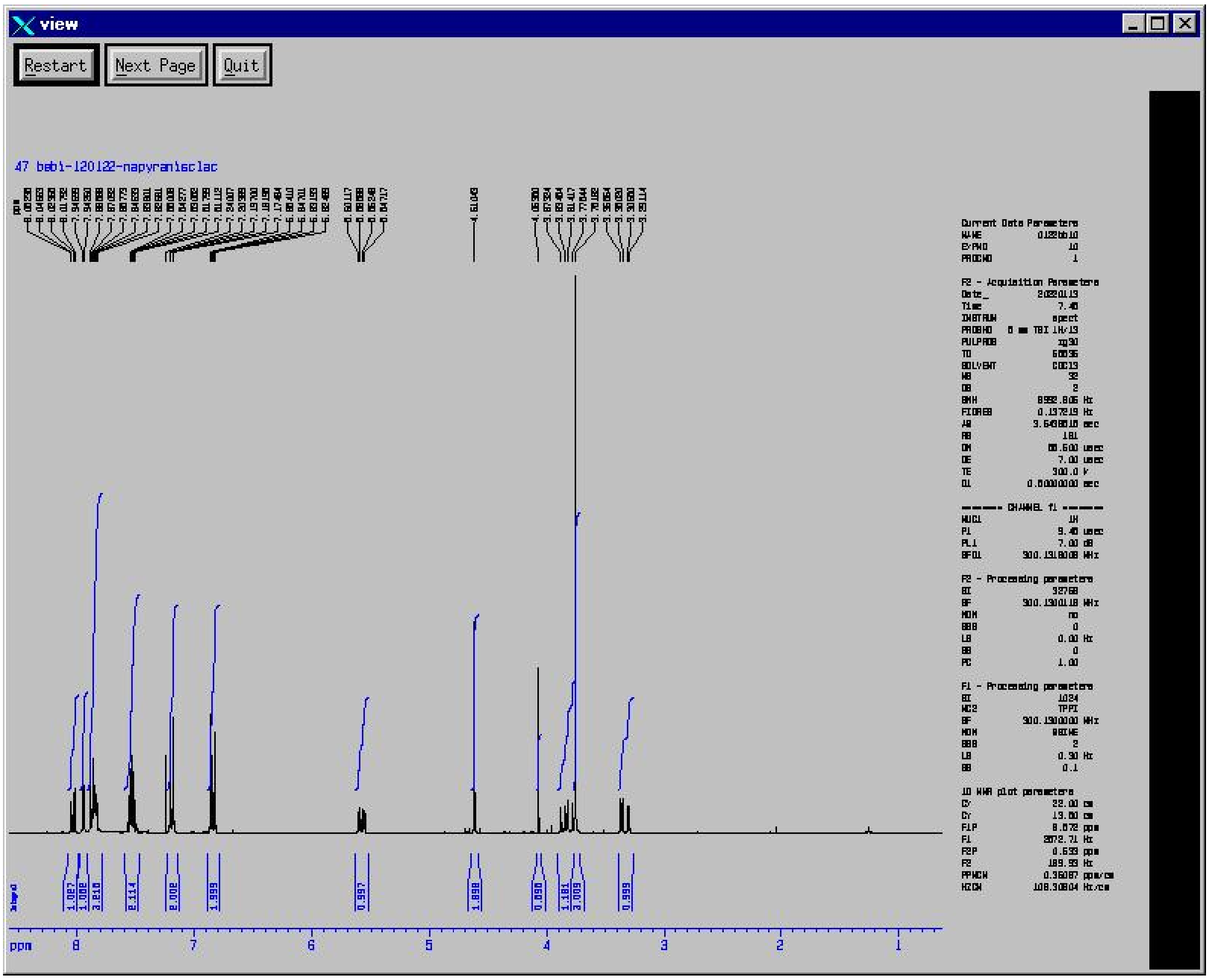

Supplementary Figure 36. ^{1}H NMR spectrum of **5a**.

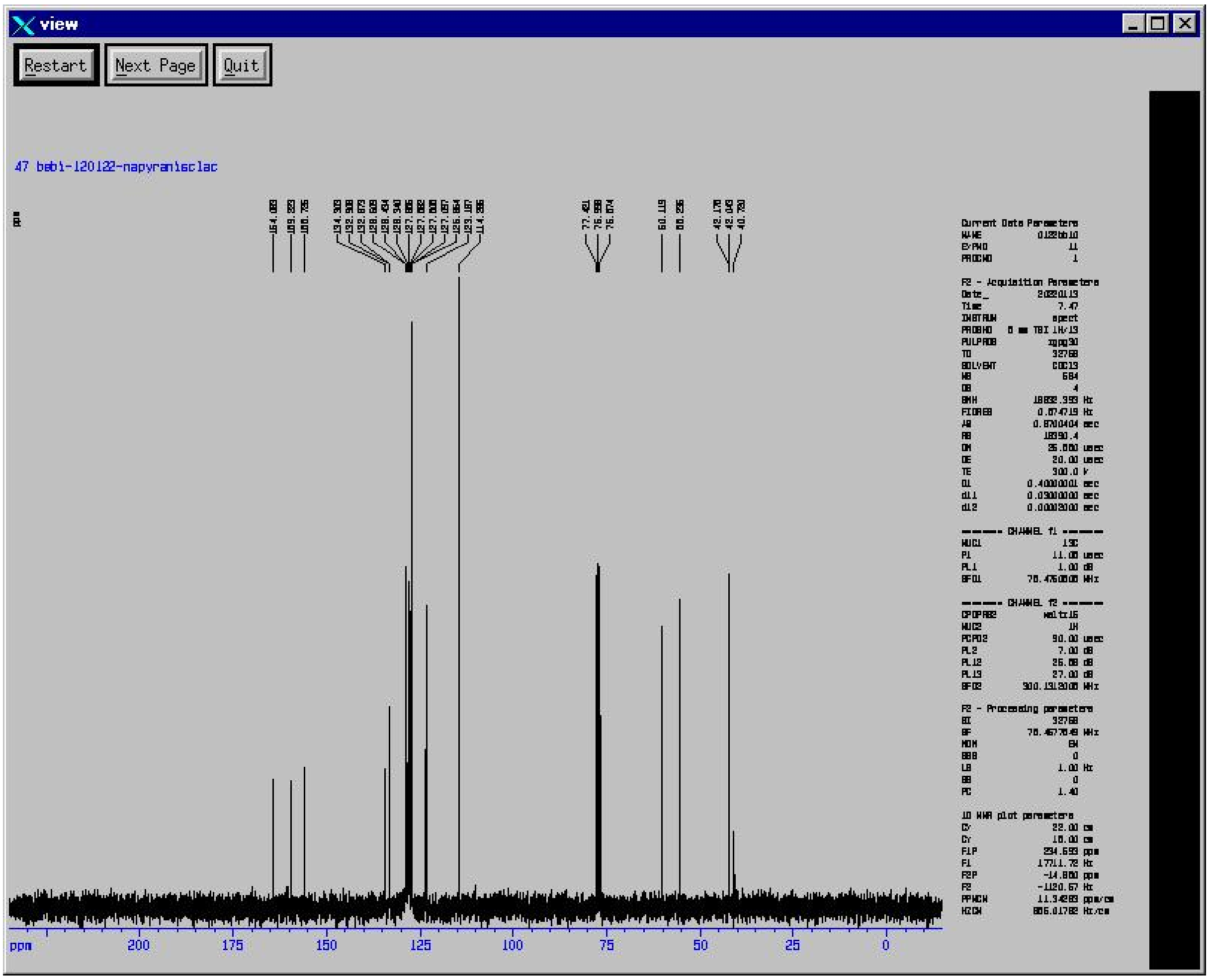

Supplementary Figure 37. ^{13}C NMR spectrum of **5a**.

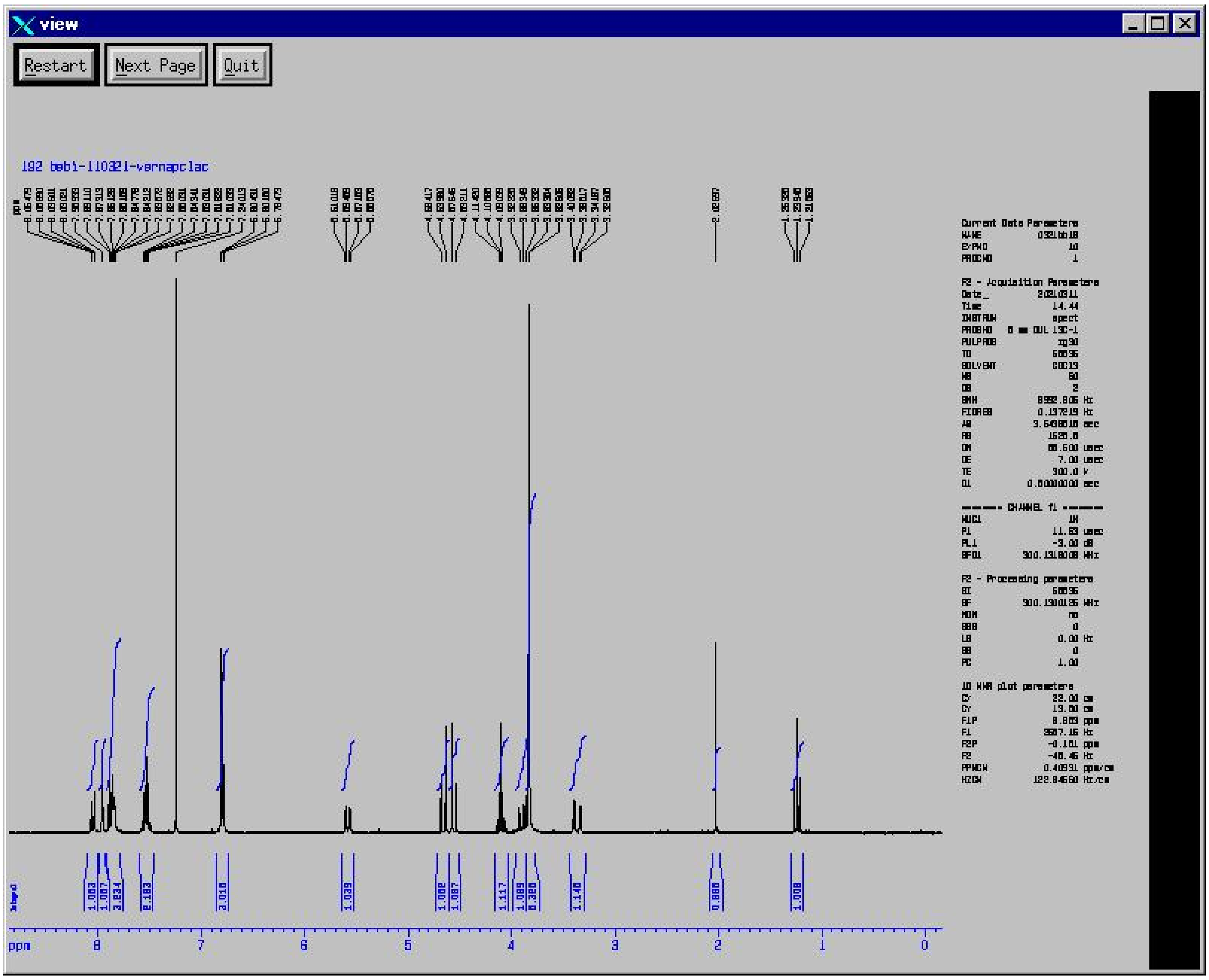

Supplementary Figure 38. ^{1}H NMR spectrum of **5b**.

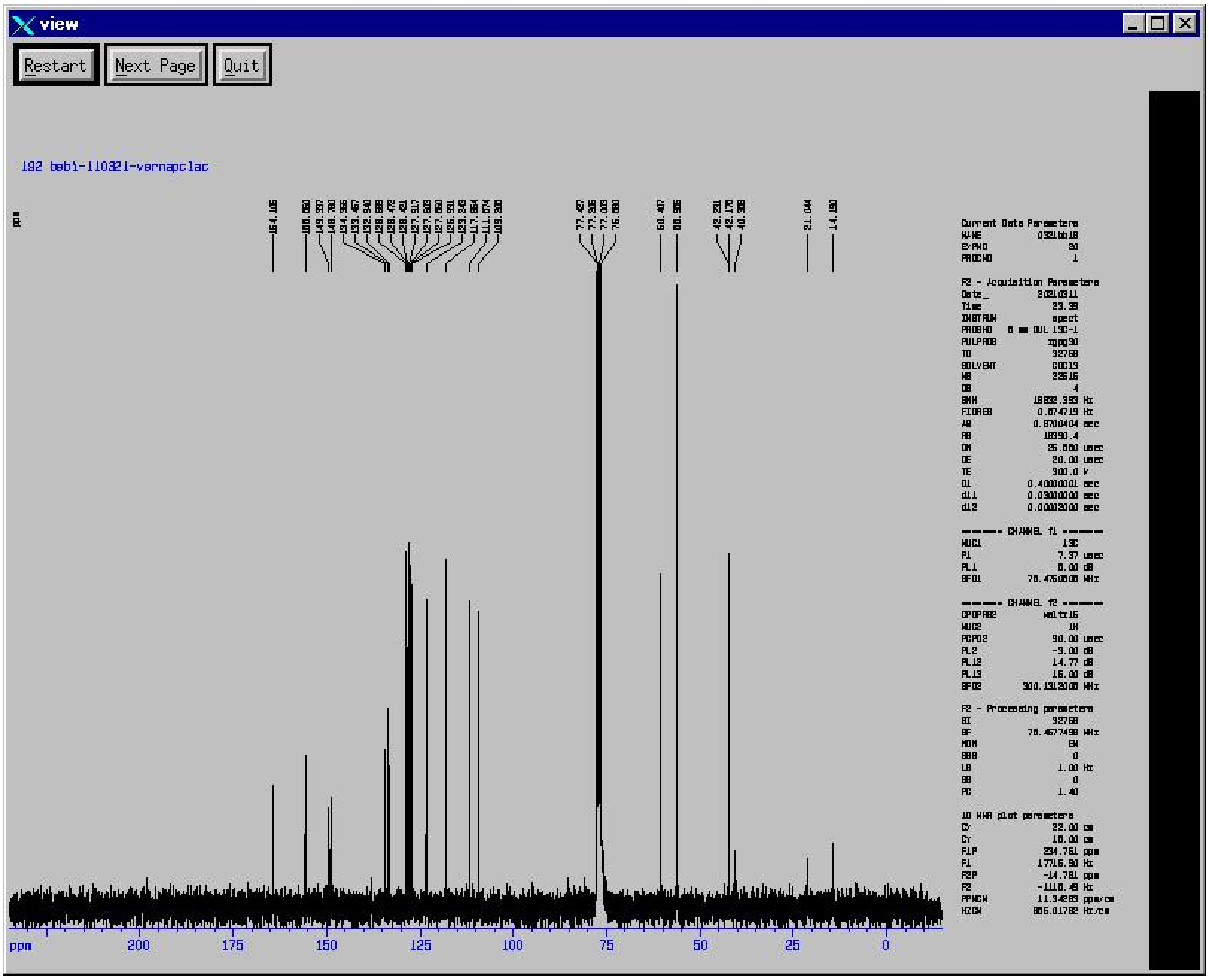

Supplementary Figure 39. ^{13}C NMR spectrum of **5b**.

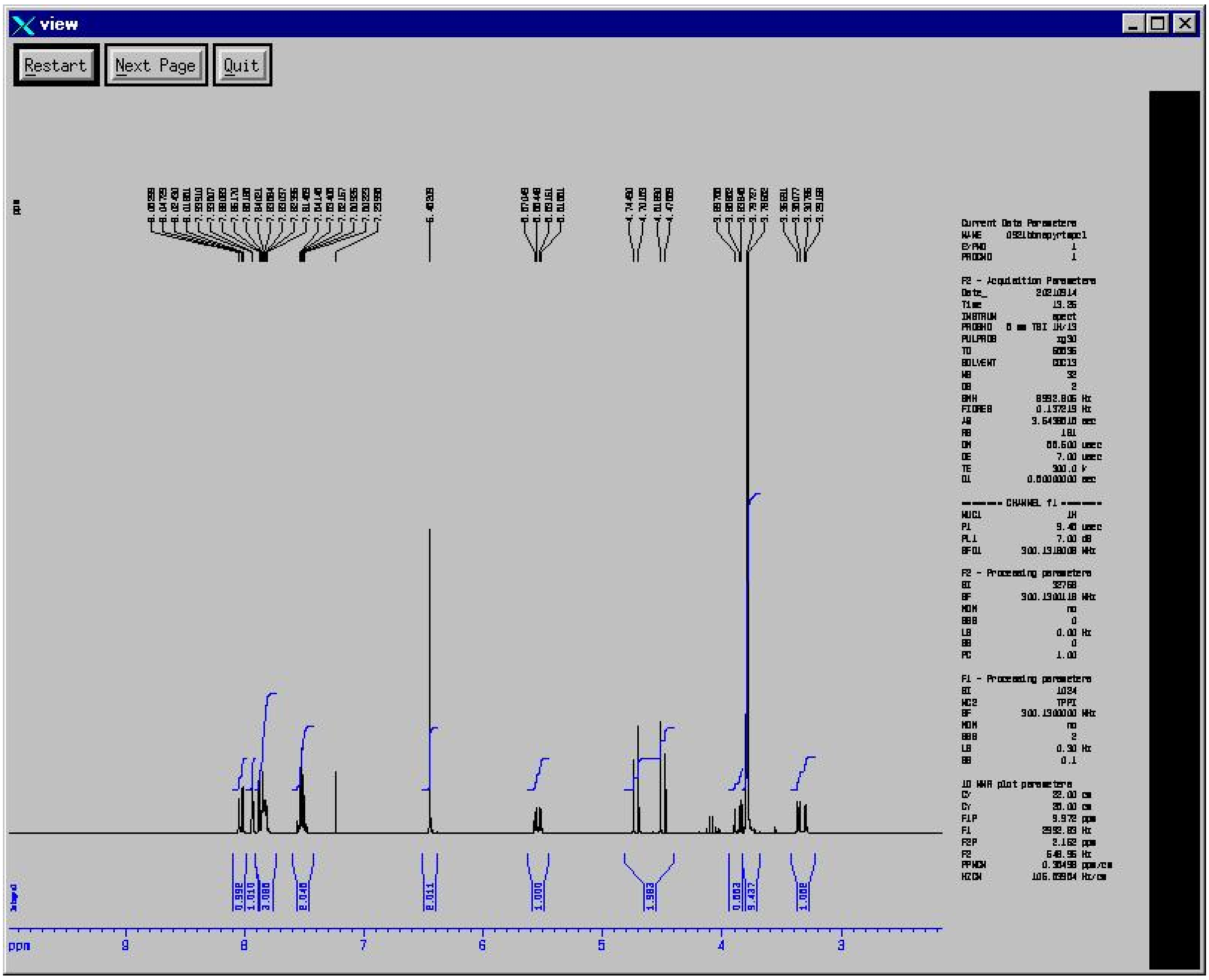

Supplementary Figure 40. ^{1}H NMR spectrum of **5c**.

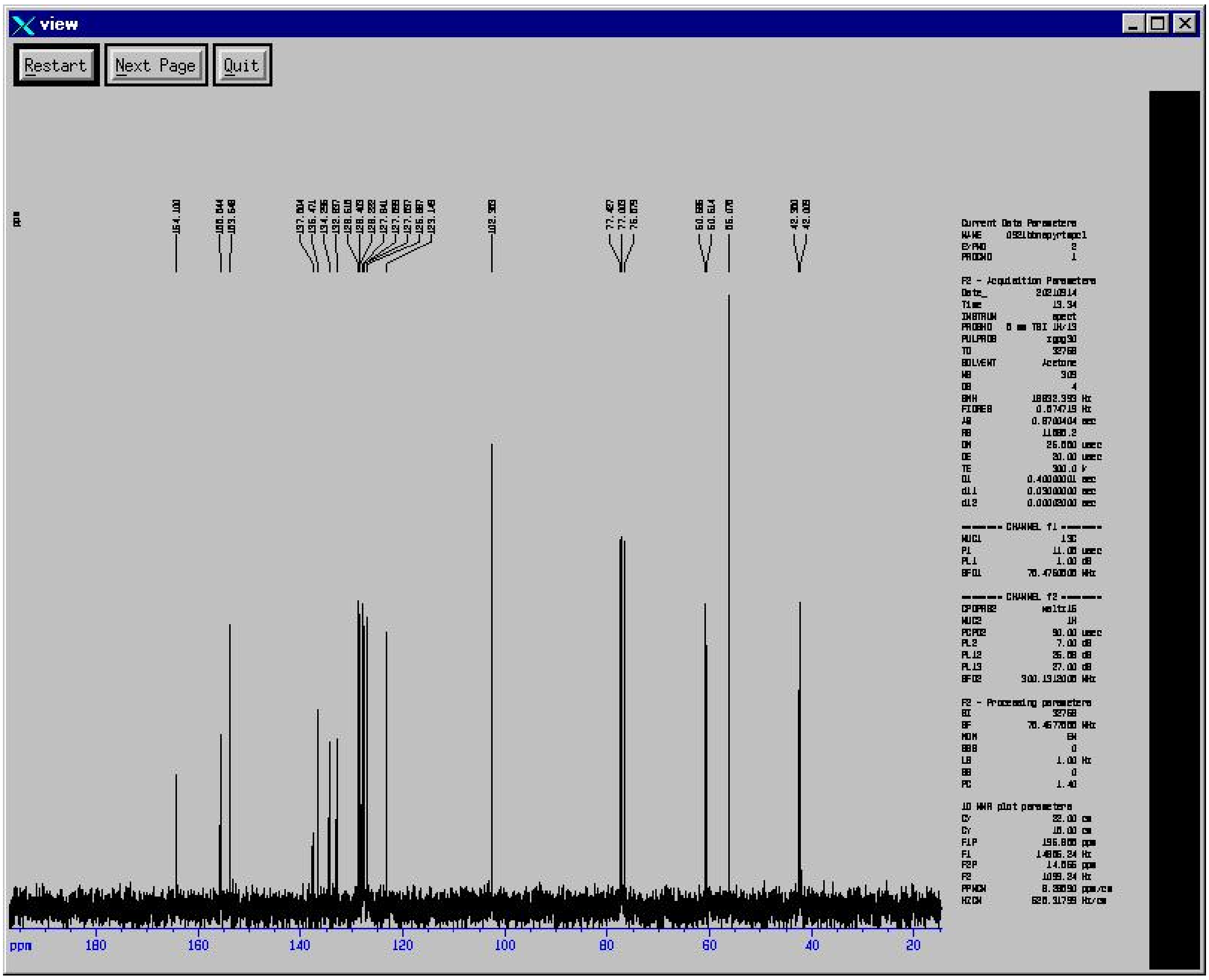

Supplementary Figure 41. ^{13}C NMR spectrum of **5c**.

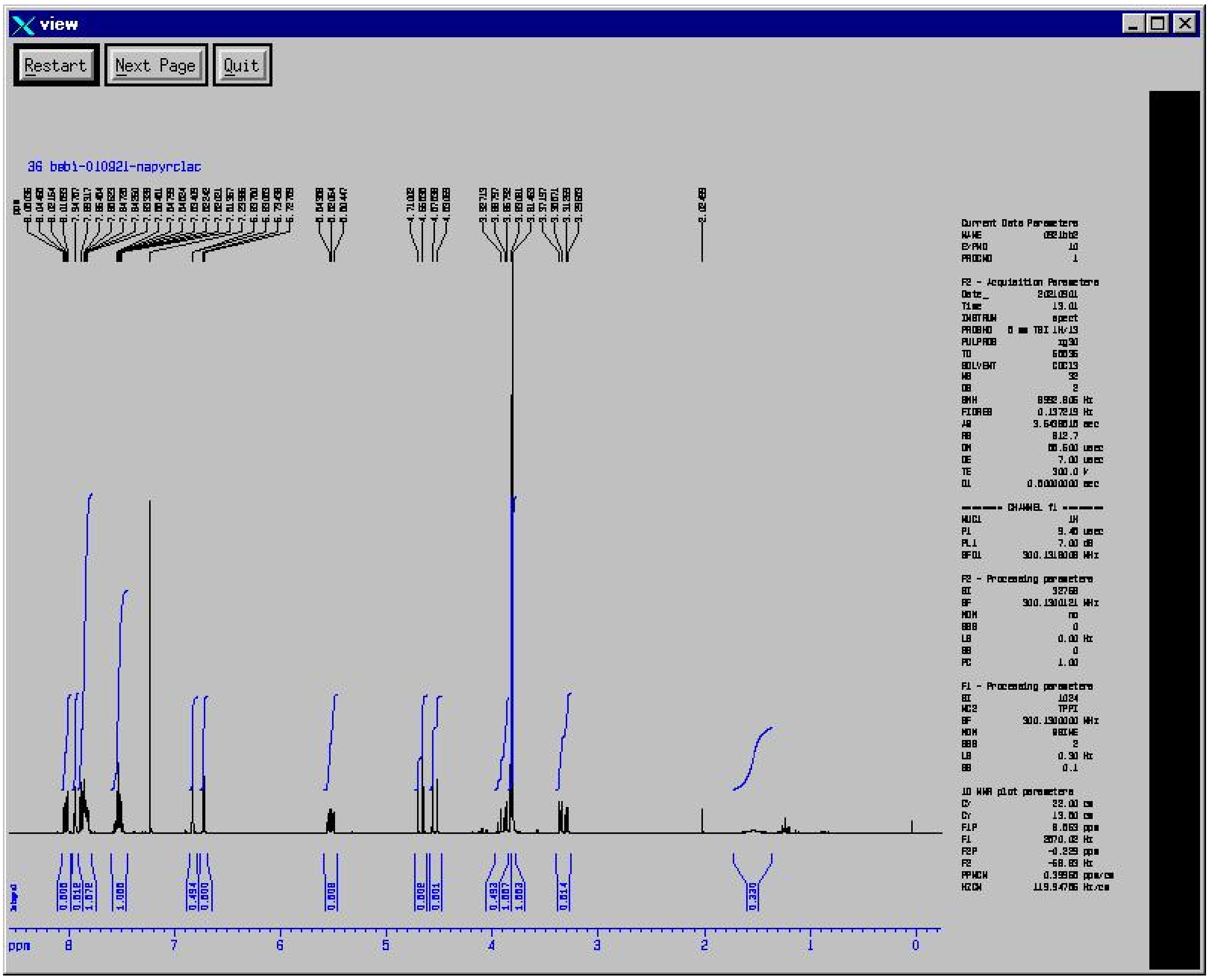

Supplementary Figure 42. ^{1}H NMR spectrum of **5d**.

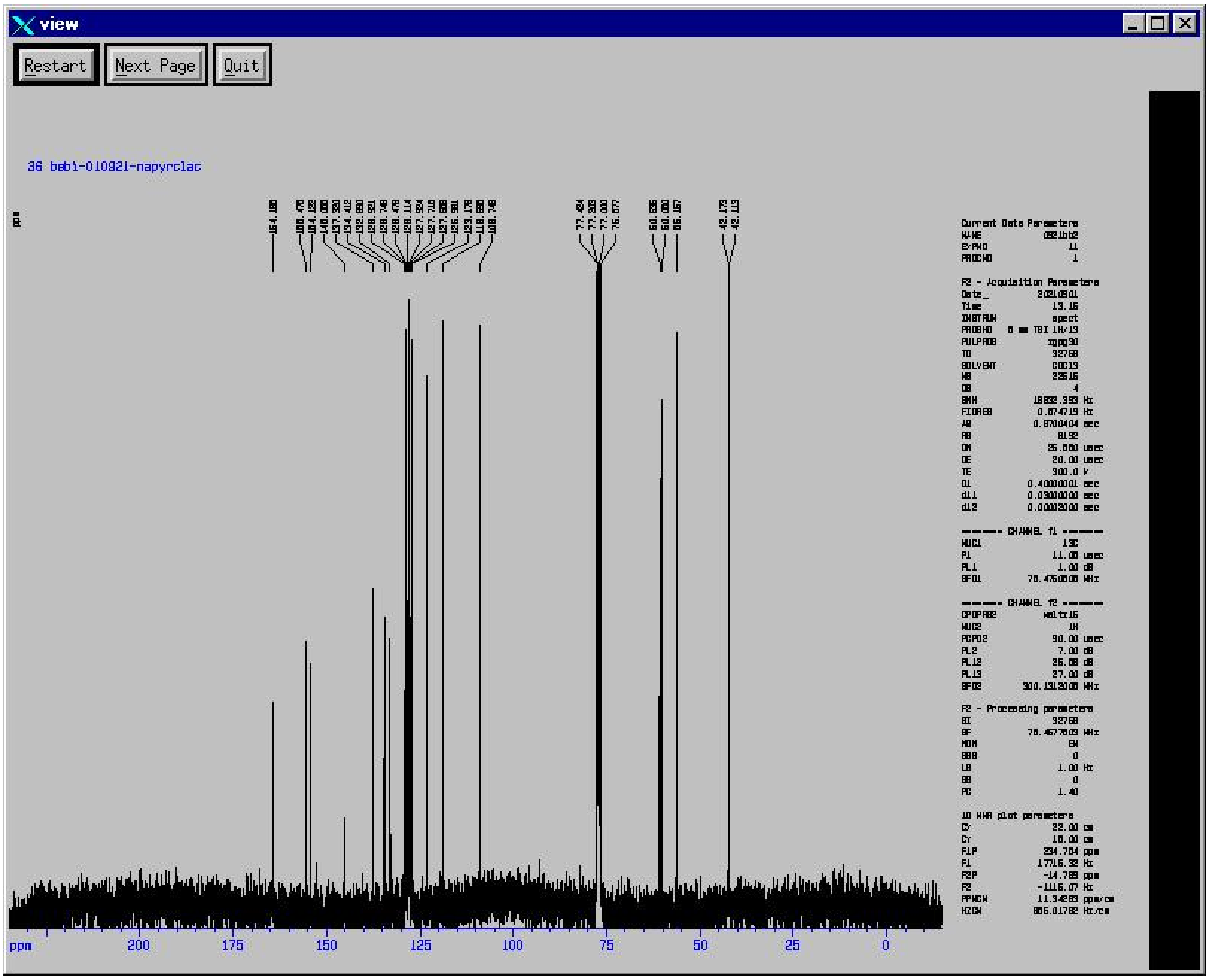

Supplementary Figure 43. ^{13}C NMR spectrum of **5d**.

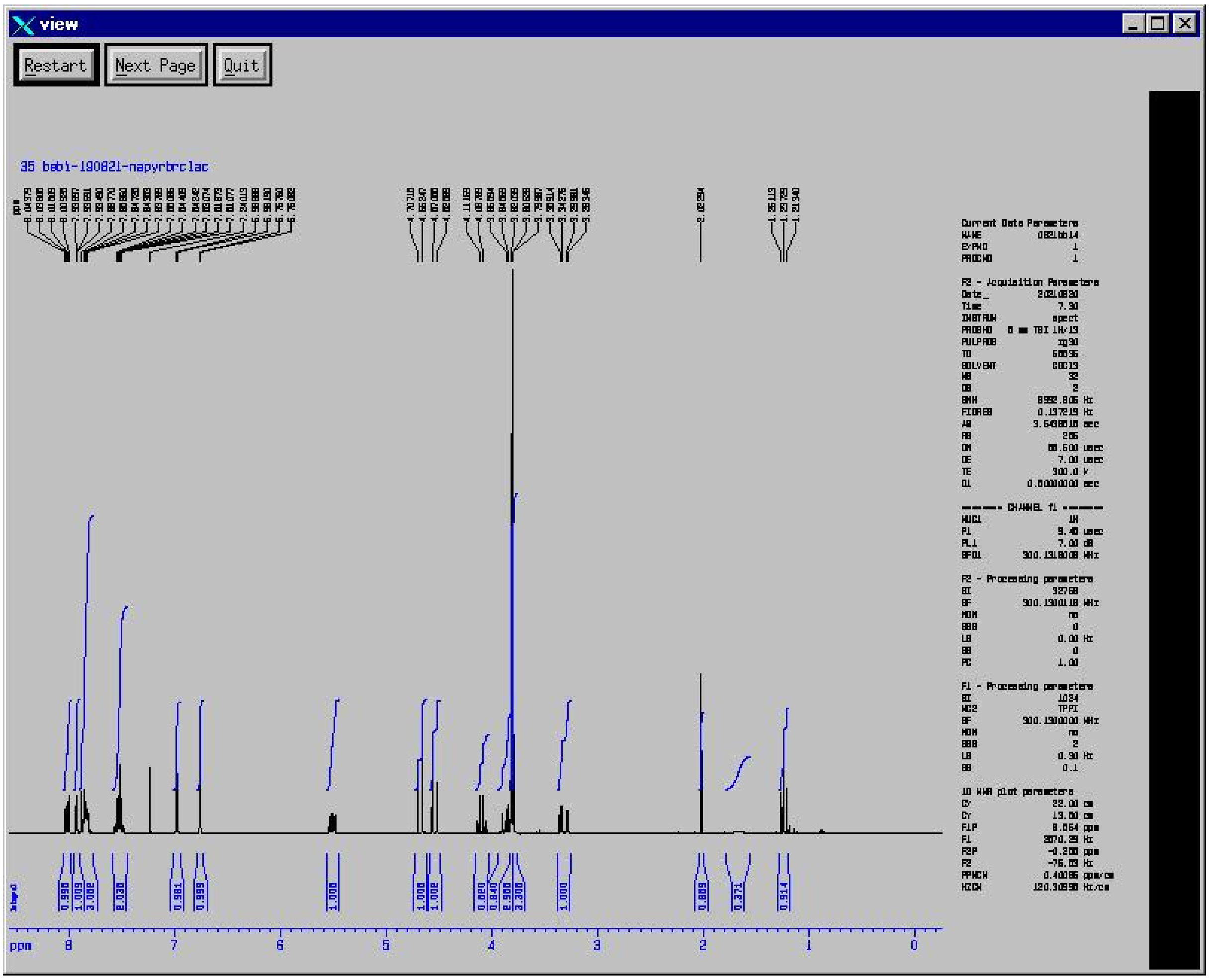

Supplementary Figure 44. ^{1}H NMR spectrum of **5e**.

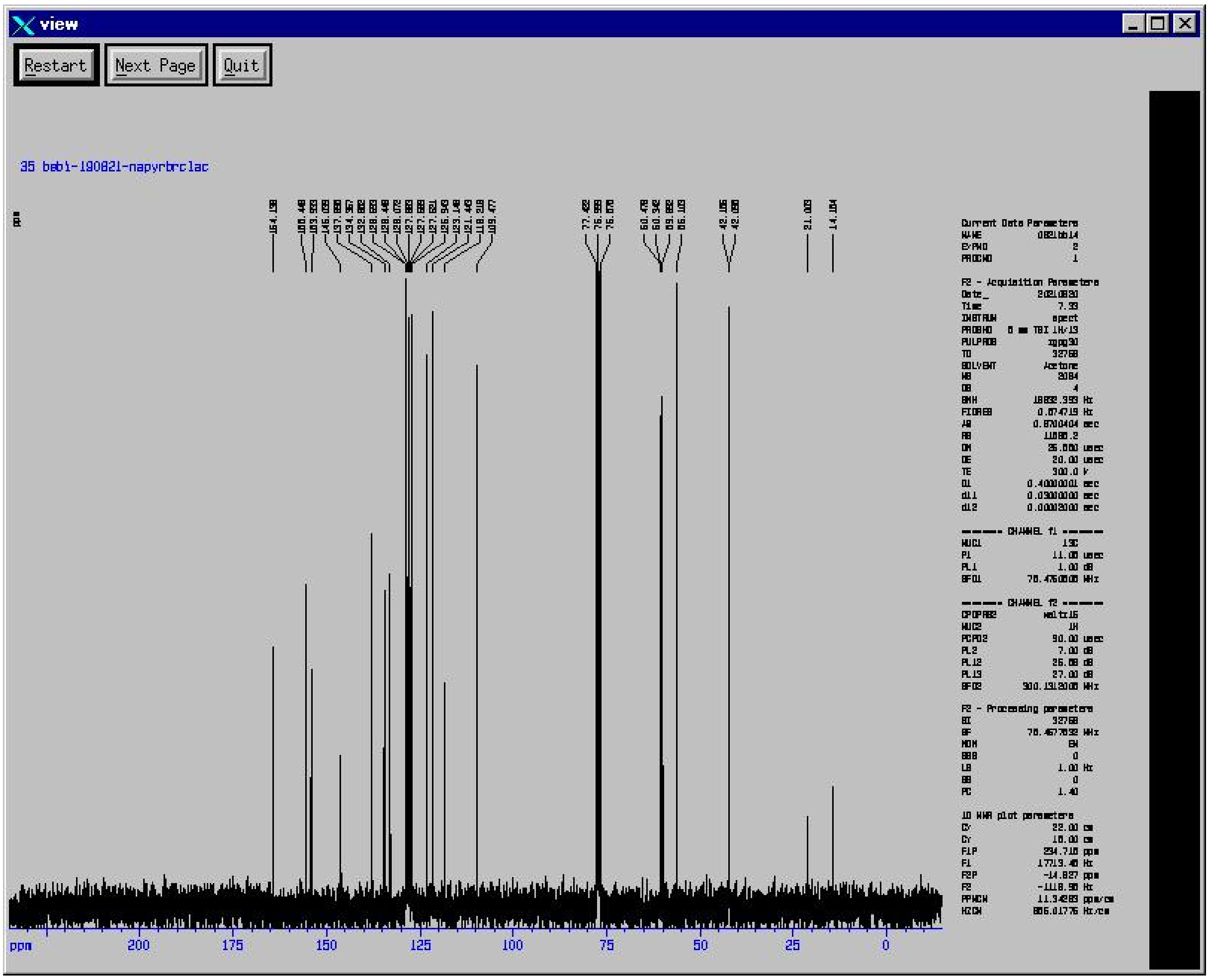

Supplementary Figure 45. ^{13}C NMR spectrum of **5e**.

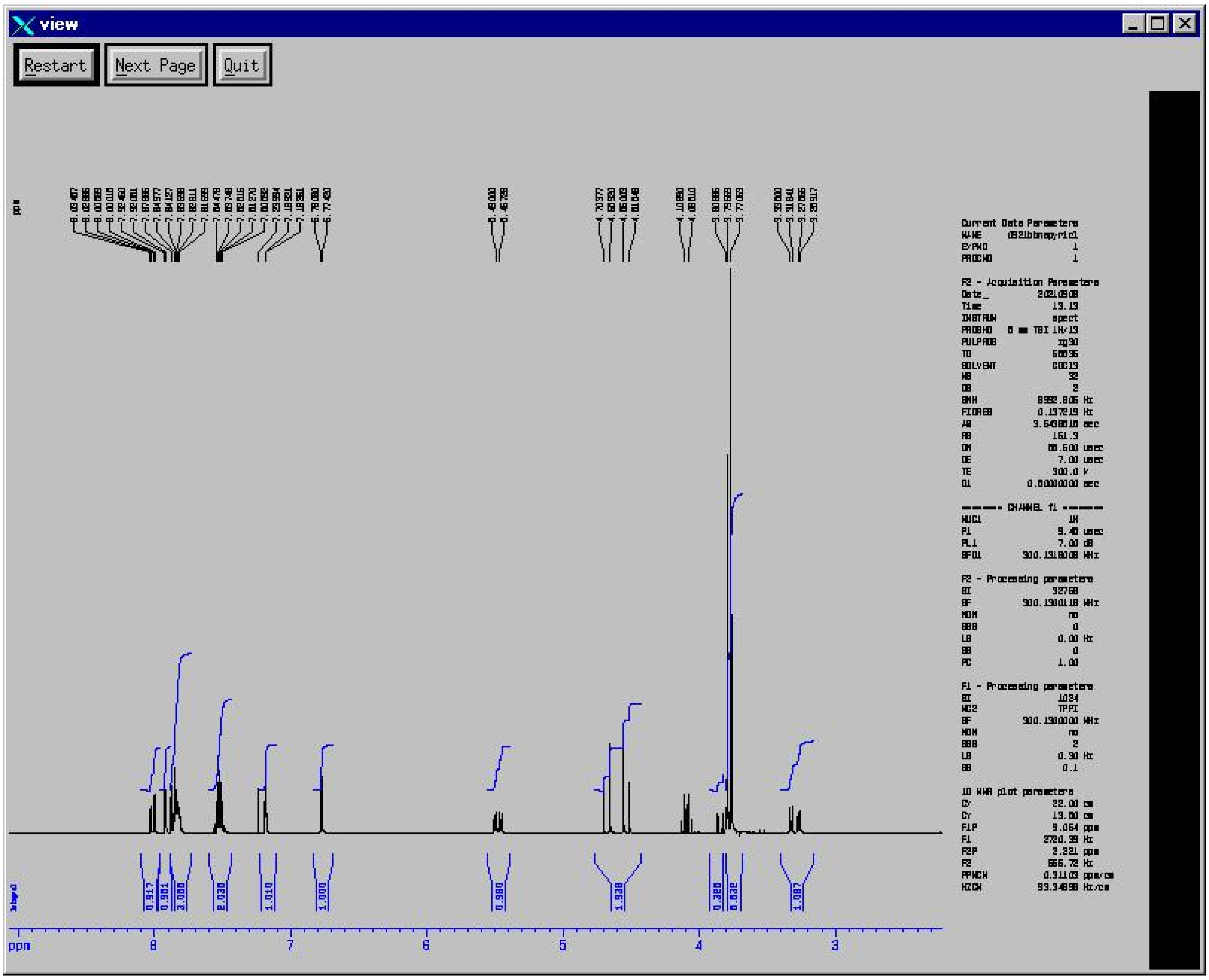

Supplementary Figure 46. ^{1}H NMR spectrum of **5f**.

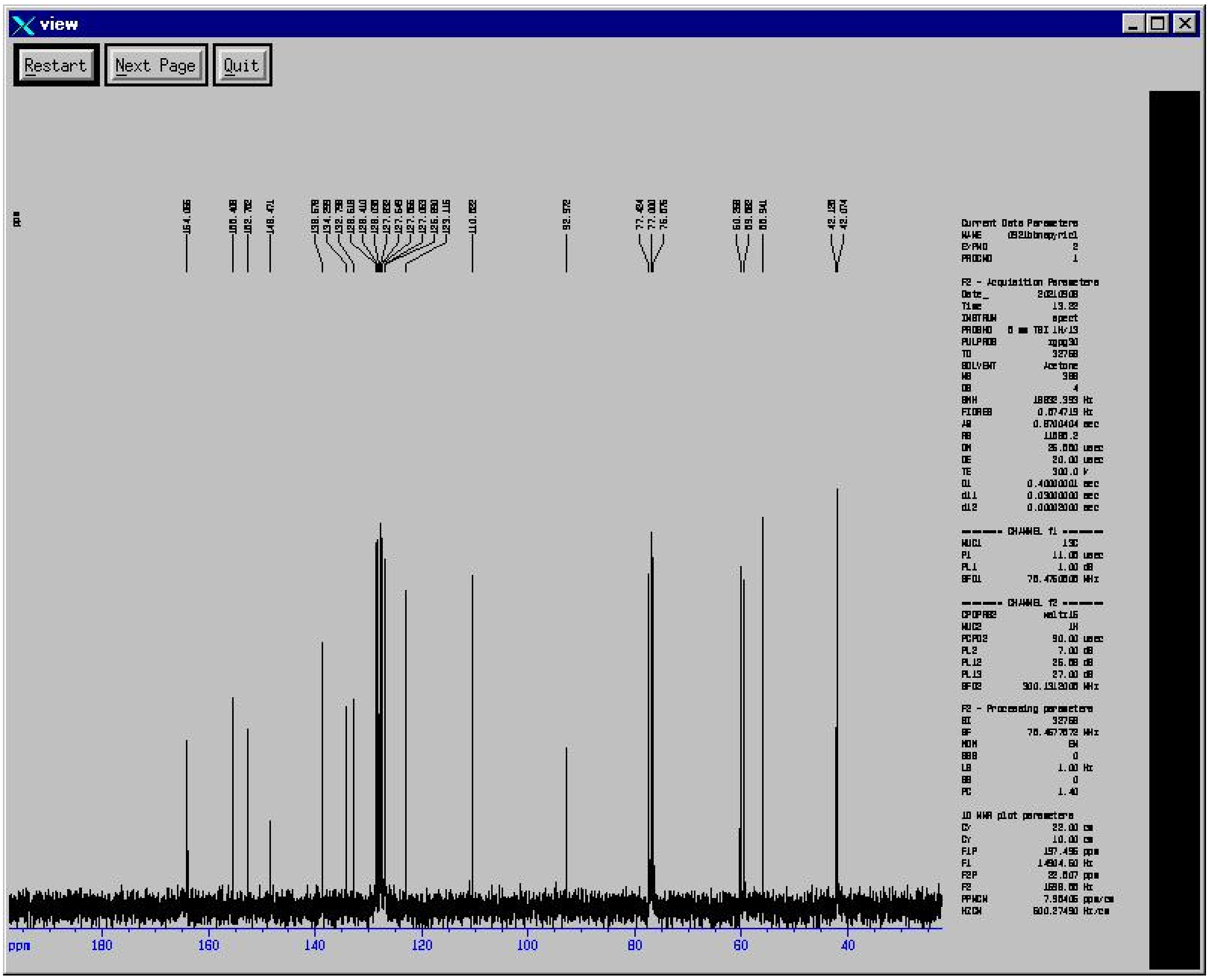

Supplementary Figure 47. ^{13}C NMR spectrum of **5f**.

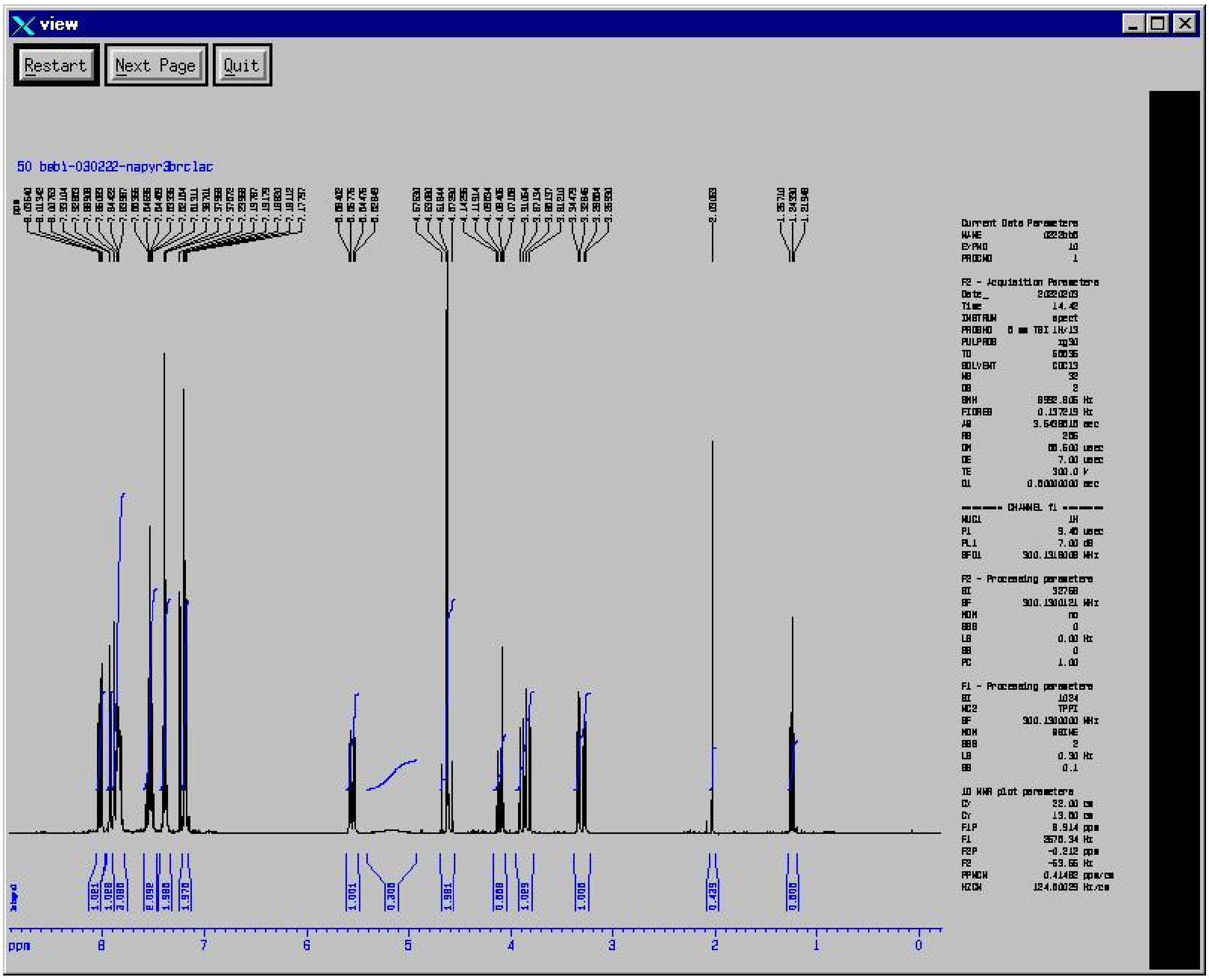

Supplementary Figure 48. ^{1}H NMR spectrum of **5g**.

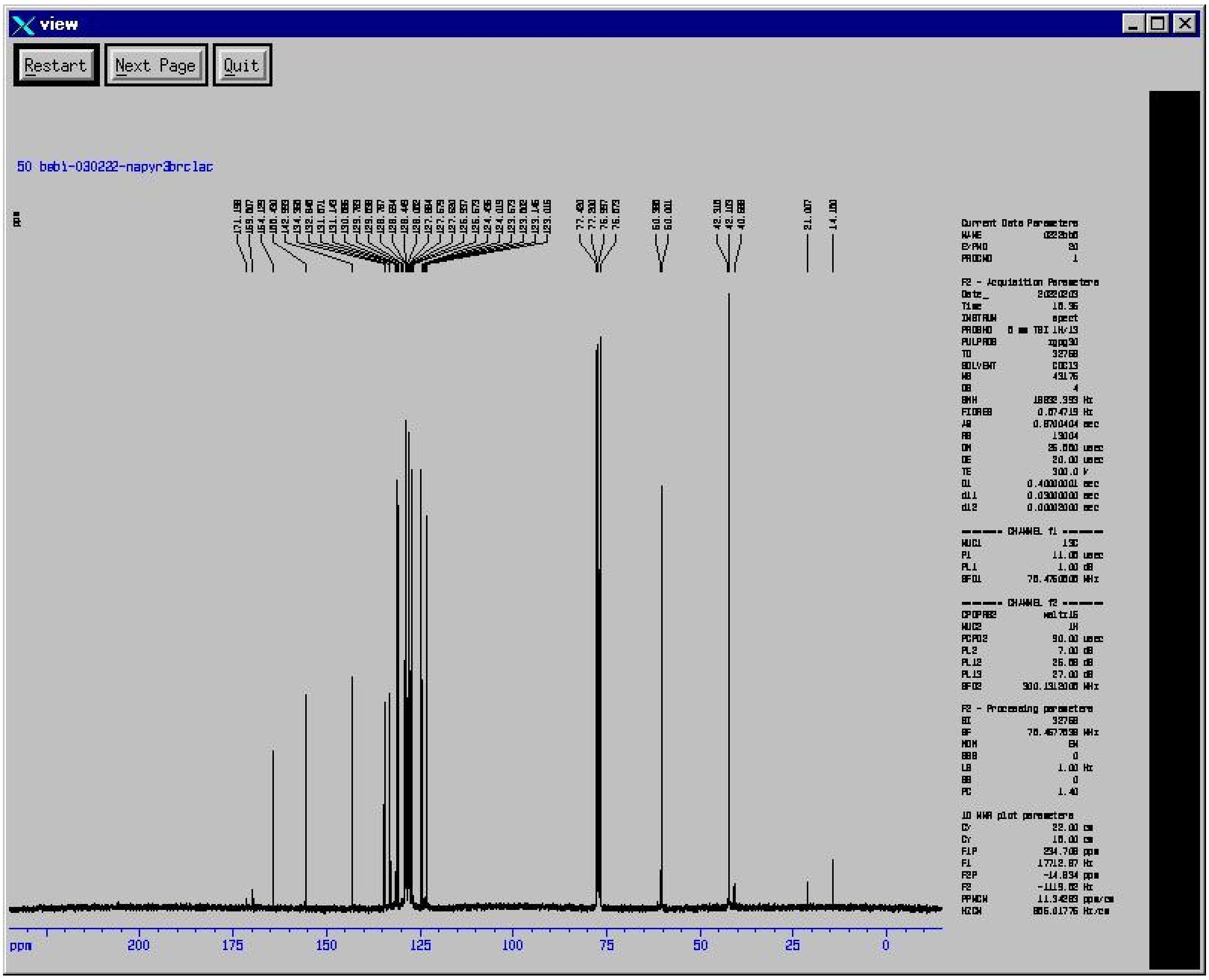

Supplementary Figure 49. ^{13}C NMR spectrum of **5g**.
